# Exome sequencing identifies novel mutation signatures of UV radiation and trichostatin A in primary human keratinocytes

**DOI:** 10.1038/s41598-020-61807-4

**Published:** 2020-03-18

**Authors:** Yao Shen, Wootae Ha, Wangyong Zeng, Dawn Queen, Liang Liu

**Affiliations:** 1 Department of Systems Biology, Columbia University, New York, New York, USA; 2 The Hormel Institute, University of Minnesota, Austin, MN USA; 3 Department of Dermatology, Columbia University, New York, USA; 4 Columbia University Vagelos College of Physicians and Surgeons, New York, NY 10032 USA

**Keywords:** Genomic instability, DNA damage and repair

## Abstract

Canonical ultraviolet (UV) mutation type and spectra are traditionally defined by direct sequencing-based approaches to map mutations in a limited number of representative DNA elements. To obtain an unbiased view of genome wide UV mutation features, we performed whole exome-sequencing (WES) to profile single nucleotide substitutions in UVB-irradiated primary human keratinocytes. Cross comparison of UV mutation profiles under different UVB radiation conditions revealed that T > C transition was highly prevalent in addition to C > T transition. We also identified 5′-A**C**G-3′ as a common sequence motif of C > T transition. Furthermore, our analyses uncovered several recurring UV mutations following acute UVB radiation affecting multiple genes including *HRNR, TRIOBP, KCNJ12,* and *KMT2C*, which are frequently mutated in skin cancers, indicating their potential role as founding mutations in UV-induced skin tumorigenesis. Pretreatment with trichostatin A, a pan-histone deacetylase inhibitor that renders chromatin decondensation, significantly decreased the number of mutations in UVB-irradiated keratinocytes. Unexpectedly, we found trichostatin A to be a mutagen that caused DNA damage and mutagenesis at least partly through increased reactive oxidation. In summary, our study reveals new UV mutation features following acute UVB radiation and identifies novel UV mutation hotspots that may potentially represent founding driver mutations in skin cancer development.

## Introduction

Today, the most common cancer affecting Caucasians is skin cancer, with a rising incidence globally^1^. Both genetic risk factors, like skin phototype and family history, as well as environmental factors, including ultraviolet radiation (UVR), chronic arsenic exposure, use of photosensitizing drugs, and immunosuppressed status, all contribute to increasing an individual’s risk for developing skin cancer^2–7^. UV radiation has the ability to exert potent carcinogenic effects, and cumulative solar exposure is increasingly being recognized as a primary risk factor for skin cancer development^8^. UVR reaches the skin in the form of two main wavelengths, UVB (290–320 nm) and UVA (320–300 nm). UVB only represents a small fraction (~5%) of total solar UVR, but is responsible for the majority of the deleterious effects on the epidermal keratinocytes, including sunburns and skin cancer. Through a lifetime of UVB exposure, genetic and epigenetic mutations accumulate, disrupting the function of key cancer genes in sun-exposed skin areas and promoting tumor initiation and progression.

While recent studies suggest that UVR enhances skin carcinogenesis through multiple processes, including immunosuppression and inflammation^2–4^, further research is needed to elucidate how UVR exerts its genotoxic and mutagenic effects, leading to the development of skin cancer. The mutation spectra in key skin cancer genes, such as TP53, have been analyzed in early studies and demonstrate the presence of canonical UV mutations in human NMSCs^9–12^. Upon exposure, UVB rays damage the DNA of keratinocytes in two major ways: the creation of cyclobutane pyrimidine dimers (CPDs) and pyrimidine 6–4 pyrimidone photoproducts (6–4PPs)^13–15^. If not repaired properly and quickly, the mutated DNA cause downstream errors in the DNA repair or the DNA replication processes. CPDs have greater mutagenic effects, as they occur more commonly and are more challenging to repair than 6–4PPs^16^. Furthermore, UVA can also exert deleterious effects directly through the introduction of single-strand DNA breaks and CPDs, or by the creation of reactive oxygen species and DNA-protein crosslinks that indirectly damage the DNA^17,18^.

A “mutation log” describes mutations that occur when cells are treated with a known mutagen. The log delineates the types and associated characteristics of the resulting mutations, including spatial distribution, occurrence of flanking bases, or presence of 5-methylcytosine^14^. UV-induced mutations include deletions, insertions, and base substitutions (transitions and transversions). Before next generation sequencing technology was developed, studies of UV mutagenesis were largely limited to a few chosen genes or transgenes that allow clonal expansion^19–24^. While these targeted methods are sensitive and have generated crucial fundamental knowledge of UV mutation characteristics, these mutational profiles may not recapitulate mutagenesis at chromosomal loci across the genome due to their very limited coverage. To obtain a comprehensive view of the global UV mutation landscape at endogenous genetic loci, we performed whole exome-sequencing (WES) studies to profile UVB-induced exome-wide mutation patterns when various doses of UVB were applied to primary human keratinocytes. To assess the impact of the chromatin environment on UV mutagenesis, we pretreated keratinocytes prior to UVB radiation with a pan-histone deacetylase inhibitor (HDACi), trichostatin A (TSA), to increase the accessibility of DNA repair machinery to genomic DNA via chromatin decondensation^25^. Subsequent bioinformatics and statistical analyses of the WES data identified novel mutagenic features of UVB radiation, as well as genes that consistently harbor recurring mutations following acute UVB exposure and are also present in human skin tumors.

## Results

### Kinetic features and distribution of UVB-induced SNVs

**Figure 1:**
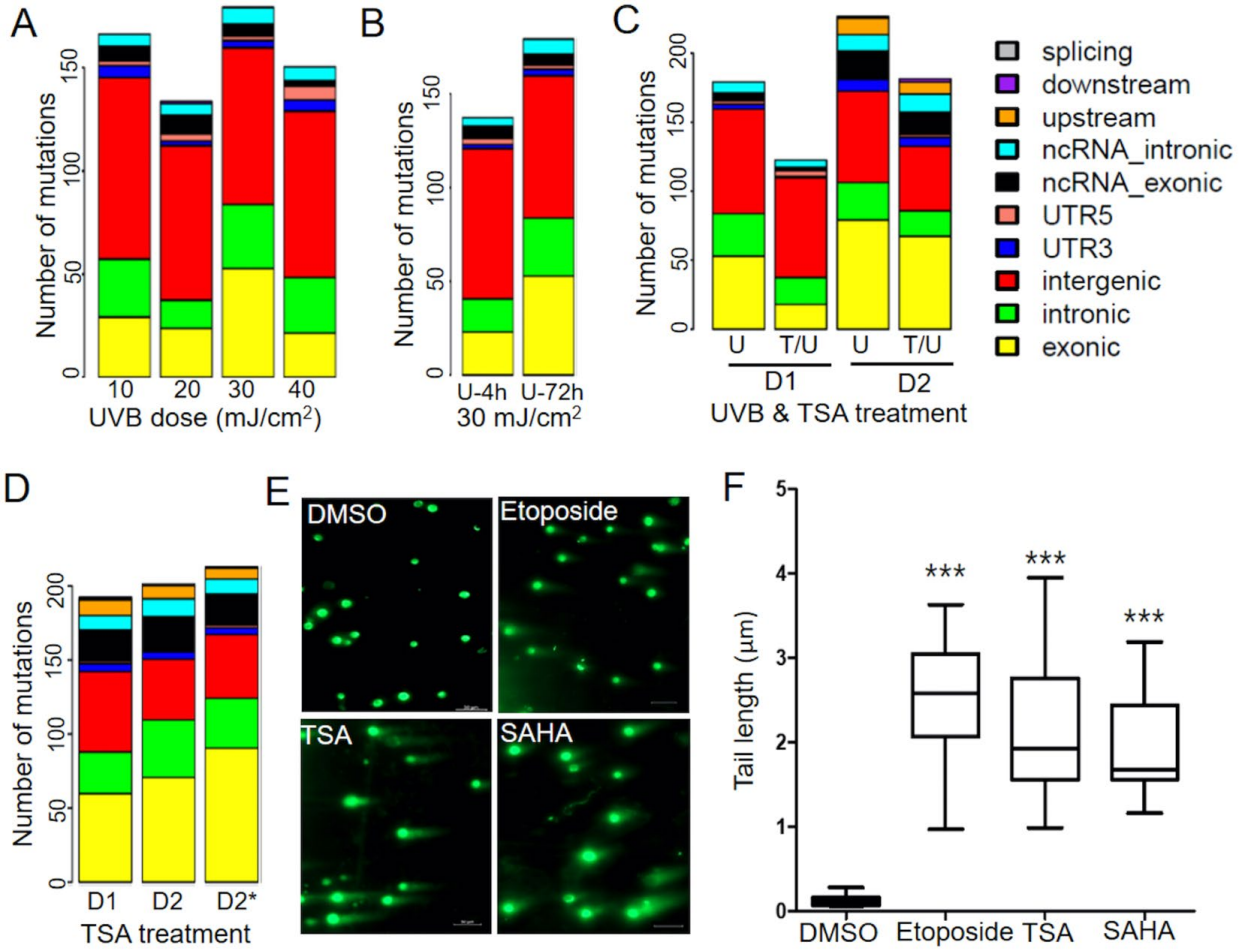
Kinetic features and genomic distribution of UVB- and TSA-induced SNVs. (**A**) The number of UV-induced SNVs and their genomic distributions following exposure to various doses of UVB radiation. (**B**) Time-dependent changes in SNV number and genomic distribution at 4 and 72 h following exposure to 30 mJ/cm^2^ UVB. (**C**) Changes in SNV number and genomic distribution following exposure to 30 mJ/cm^2^ UVB with or without TSA pretreatment. U: UVB radiation only; T/U: TSA pretreatment followed with UVB radiation; D1 and D2 refer to keratinocytes from two different donors. (**D**) The number of SNVs and their genomic distributions following TSA treatment alone. D2*: cells were treated with TSA twice within four days. (**E**) TSA and SAHA induced DNA breaks as measured by comet assay. Etoposide treatment was included as a positive control. (**F**) Quantification of the comet tail lengths using image J following treatment with DMSO, etoposide, TSA, and SAHA, respectively. Representative comets from four independent experiments were included in the analysis (n = 18; ***p < 0.001).

Conventional studies of the UV mutation log employ direct sequencing of selected DNA sequences, such as the *Aprt* gene, in cultured cells following UV radiation to characterize UV mutation types and spectra. To obtain an unbiased view of UV-induced mutation profiles at endogenous genetic loci across the genome, we performed WES studies on primary human keratinocytes that were irradiated with various doses of UVB (10, 20, 30 and 40 mJ/cm^2^). Sequencing reads from UVB-irradiated cells were mapped and compared to sequencing reads derived from non-irradiated control cells to eliminate SNVs due to polymorphisms or background mutations. The resulting SNVs from different UVB doses were analyzed to assess the kinetic features of UVB-induced mutation types and their genomic distributions. As summarized in Fig. 1, we found that SNVs were enriched in intergenic regions more often than in gene bodies (both exons and introns) across all UVB doses, probably due to a lack of transcription-coupled DNA repair at these untranscribed regions^26^. Furthermore, while there was an increase in SNV number from 4 to 72 h following exposure to identical UVB radiation doses (30 mJ/cm^2^), we observed no clear trend of dose-dependent changes in SNV numbers (Fig. 1A,B). In contrast to the large number of mutations reported in human skin tumors^27–29^, the number of UV-induced SNVs was relatively small, which is attributable to the difference between acute UV radiation and repetitive UV exposure causing accumulation of mutations over time in addition to non-UV mutations during tumor progression.

Chromatin conformation and remodeling play a major role in regulating DNA accessibility for UV-induced DNA damage repair in chromatin^30–32^. To examine the impact of the chromatin environment on UV mutagenesis, we performed similar WES analyses using keratinocytes pretreated with trichostatin A (TSA), a classic histone deacetylase inhibitor (HDACi), that is widely used in epigenetic gene regulation studies. TSA shares structural similarity with suberoylanilide hydroxamic acid (SAHA), an FDA-approved epigenetic drug for clinical treatment of cutaneous T-cell lymphoma through its HDACi activity. We posited that TSA treatment would decondense chromatin, facilitating the access of DNA repair machinery to repair UV-induced DNA lesions to reduce UV mutagenesis. Consistent with our hypothesis, we found that TSA pretreatment followed by UVB led to pronounced decreases in the total number of SNVs in keratinocytes from two different donors (D1 and D2, Fig. 1C). Unexpectedly, however, we observed that TSA alone caused substantial SNVs in non-irradiated cells from both donors (Fig. 1D), suggesting that TSA itself may be a mutagen, which has not been reported previously. To test if repetitive TSA treatment could substantially increase SNVs, we treated D2 cells with TSA twice within four days. WES revealed only a moderate increase (<10%) in the total number of SNVs with minimal effects on SNV distribution within the genome (D2* in Fig. 1D). While UV induced mutations occurred in intergenic regions, TSA-induced mutations occurred more frequently within gene bodies (exons and intron, Fig. 1D). To test whether TSA induced mutations through DNA damage, we performed DNA comet assays to measure DNA strand breaks in primary keratinocytes treated with TSA, SAHA, or vehicle control (DMSO). We found that TSA and SAHA both caused substantial DNA damage (Fig. 1E,F), which was accompanied by increased cellular oxidative stress (Supplementary Fig. 1).

### UVB- and TSA-induced mutation spectra and signatures

**Figure 2:**
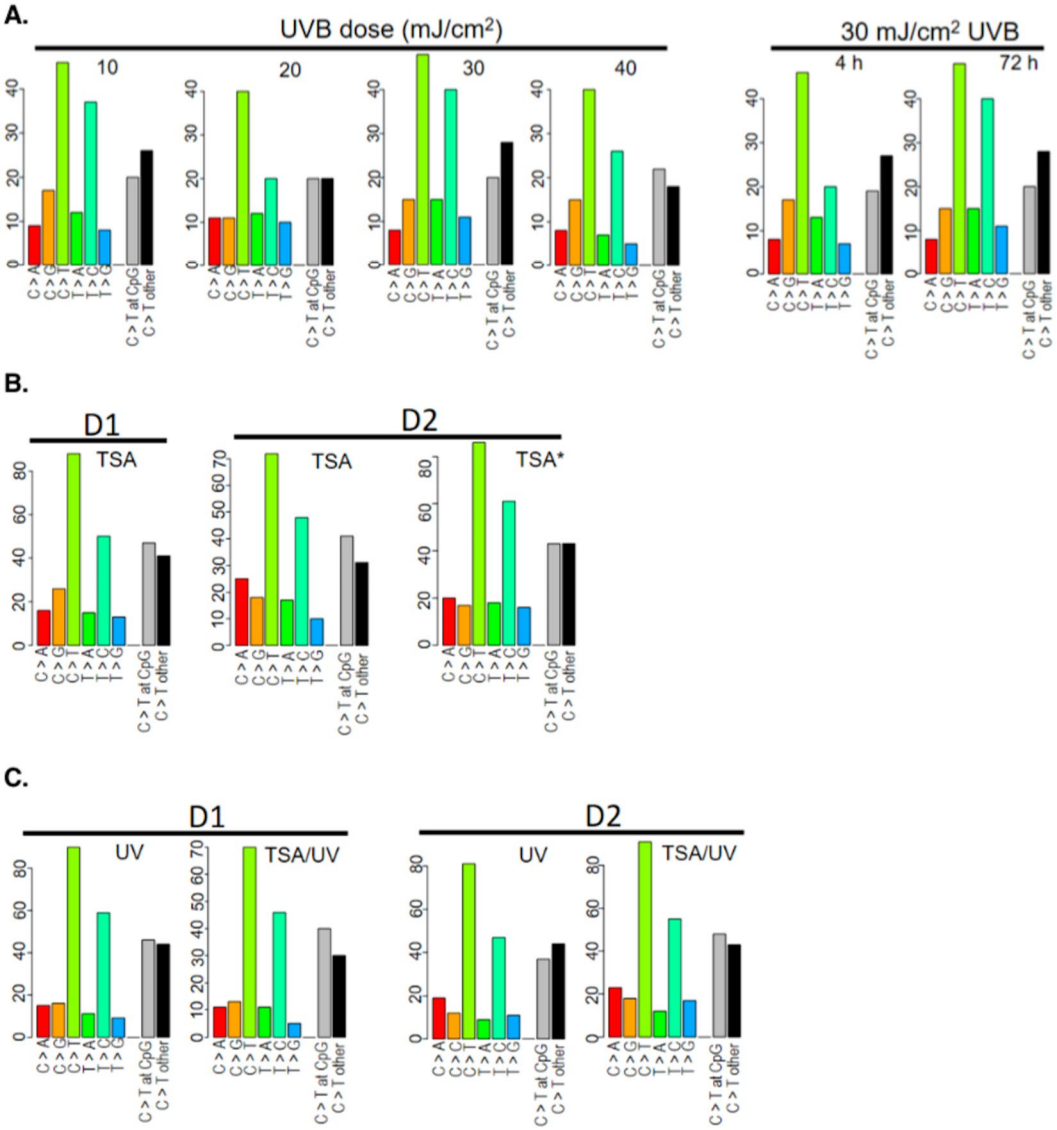
UVB- and TSA-induced mutation spectra and signatures. (**A**) Left panel: graphic illustration of mutation types and spectra following exposure to different doses of UVB radiation. Right panel: mutation types and spectra at 4 and 72 h following exposure to 30 mJ/cm^2^ UVB radiation. (**B**) TSA-induced mutation types in keratinocytes from two different donors (D1 and D2). TSA*: D2 cells were treated with TSA twice within four days. (**C**) Changes in mutation types and spectra following exposure to 30 mJ/cm^2^ UVB with or without TSA pretreatment in D1 and D2 cells.

Next, we analyzed mutation types following TSA treatment with or without UVB radiation. We classified SNVs into six mutation types of single-base substitutions (C > A/G > T, C > G/G > C, C > T/G > A, T > A/A > T, T > C/A > G, and T > G/A > C) as previously reported^33^. Consistent with the canonical UV mutation signature observed in skin tumor tissues, we found C > T transition to be the most frequent UVB-induced mutation type (Fig. 2A), which is also congruent with the known role of UV light in inducing pyrimidine dimers whose erroneous repair leads to C > T transition at CpC or TpC dinucleotides. Of note, the frequency of C > T transition in human keratinocytes following acute UVB exposure (~30%) was much lower than that reported in human skin tumors (70–90%). Additionally, we found T > C transition to be the second most frequent mutation type (~25%) following acute UVB exposure, which has not been described in previous studies (Fig. 2A). When comparing mutation types between 4 h and 72 h following exposure to 30 mJ/cm^2^ of UVB, we found an increase only in T > C transition that accounted for the increase in total SNVs, while the other mutation types remained relatively stable (Fig. 2A).

CpG dinucleotides are known to be preferred sites of UV mutation due to CPD formation, particularly at methylated CpG sites within cancer genes such as *TP53*. These C and 5-methylcytosine (5mC) residues can also spontaneously deaminate to uracil and thymine, respectively, which, if not repaired, will result in C > T/G > A transitions^34,35^. Indeed, we found that approximately 50% of UV-induced C > T transitions occurred within CpG sequences (Fig. 2A), although the methylation status of these CpG sites is unknown. Of note, TSA-induced mutations bear a strong similarity with UVB-induced mutations, with predominant C > T transitions followed by T > C substitutions (Fig. 2B). Consistent with their similar mutagenic profiles, TSA pretreatment followed by UVB radiation produced a mutation profile with similar mutation types and spectra (Fig. 2C). Surprisingly, although both UVB and TSA seem to be mutagenic, TSA pretreatment followed by UVB radiation did not exert an additive effect, but instead led to a modest decrease in the total number of SNVs compared to UVB alone (Fig. 2C). It is conceivable that TSA treatment decondenses chromatin to facilitate the repair of UVB-induced DNA damage, while UVB radiation may in turn activate the DNA damage response to enhance repair of TSA-induced DNA damage, thereby reducing the total number of mutations.

In addition to mutations derived from UV-induced DNA damage, endogenous mutations can also arise spontaneously as a result of UV-induced dysregulation of DNA repair enzymes. The APOBEC family of proteins, for example, represents a group of cytidine deaminases involved in endogenous mutagenesis and cancer clonal evolution^36,37^. Mutation signatures associated with dysregulated APOBEC proteins in human cancers include C > T and C > G substitutions^38^. In our study, C > G substitution accounts for the third most frequent UVB-induced mutation type (Fig. 2A). APOBEC3C is the most abundantly expressed APOBEC family member in both melanocytes and keratinocytes (Supplementary Fig. 2). Following acute UVB exposure, APOBEC3C exhibited various degrees of upregulation, which might have contributed indirectly to the high C > G mutation frequency.

### Sequence context of UVB-induced SNVs

**Figure 3:**
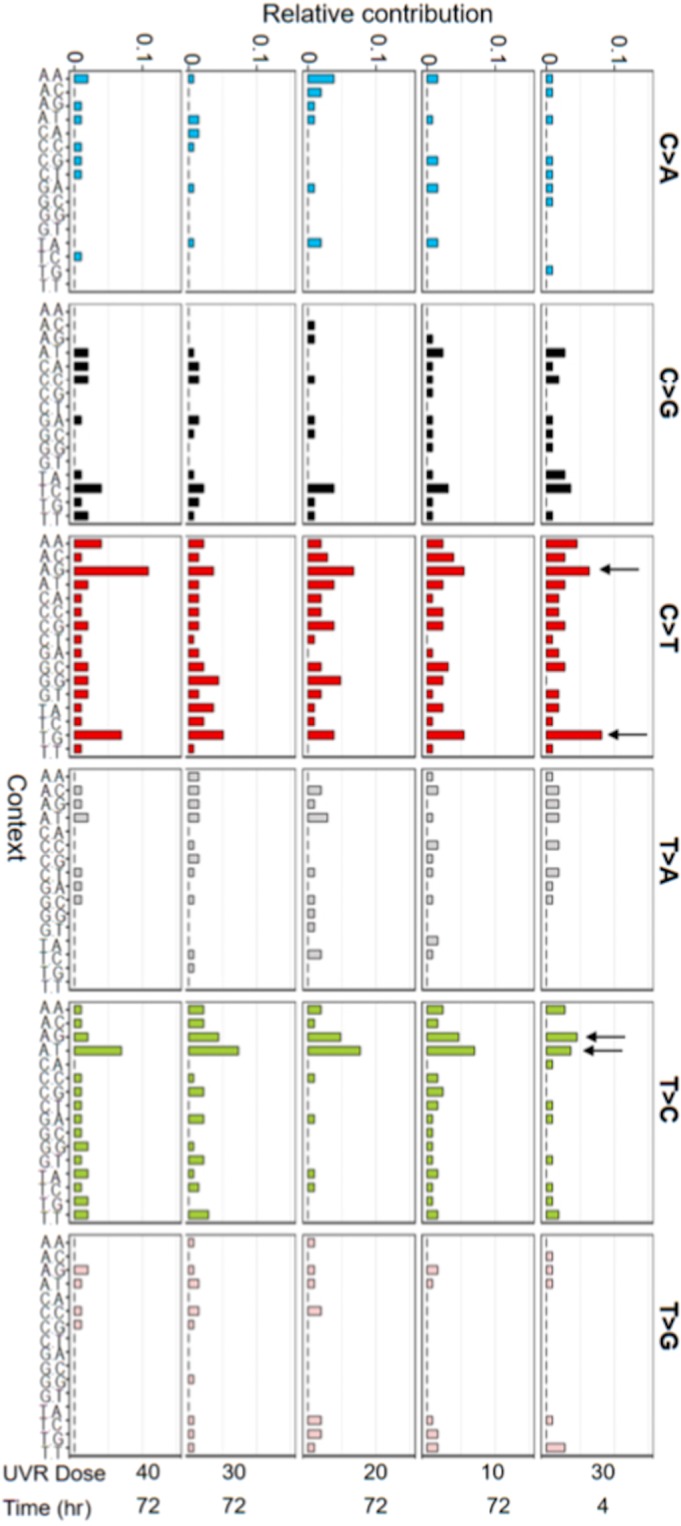
Overview of UV-induced mutation types and their respective sequence contexts following different doses of UVB radiation. Arrows indicate preferred sequence motifs for C > T and T > C transitions, two dominant UVB mutations.

Different mutational processes cause signature mutations in characteristic sequence contexts^34,39^. The canonical UV-induced C > T transition occurs predominantly within a 5′-(C/T)**C**N-3′ base context based on cell culture studies and sequencing data from human skin tumors^39^. After analyzing the sequence context of C > T mutations following UVB radiation (Fig. 3**)**, we observed two predominant sequence contexts for C > T mutation, including a 5′-T**C**G-3′ motif, which is consistent with the canonical 5′-(C/T)**C**N-3′ motif, and a distinct 5′-A**C**G-3′ motif. Interestingly, both of these motifs contain a CpG site, a major target of UV mutagenesis in the mammalian genome^40,41^. Given that 5mCpG is especially affected by the longer UV wavelength-induced (UVB and UVA) C > T transition^40^, it is unsurprising that the 5′-A**C**G-3′ motif was not discovered in previous studies that used only the short wavelength UVC radiation^39^. For UVB-induced T > C transition, we found 5′-A**T**T-3′ and 5′-A**T**G-3′ to be preferred sequence motifs. Taken together, these findings revealed novel features of UV mutagenesis that were not reported in previous studies.

### Acute UV-induced mutation hot spots in skin tumor tissues

**Figure 4:**
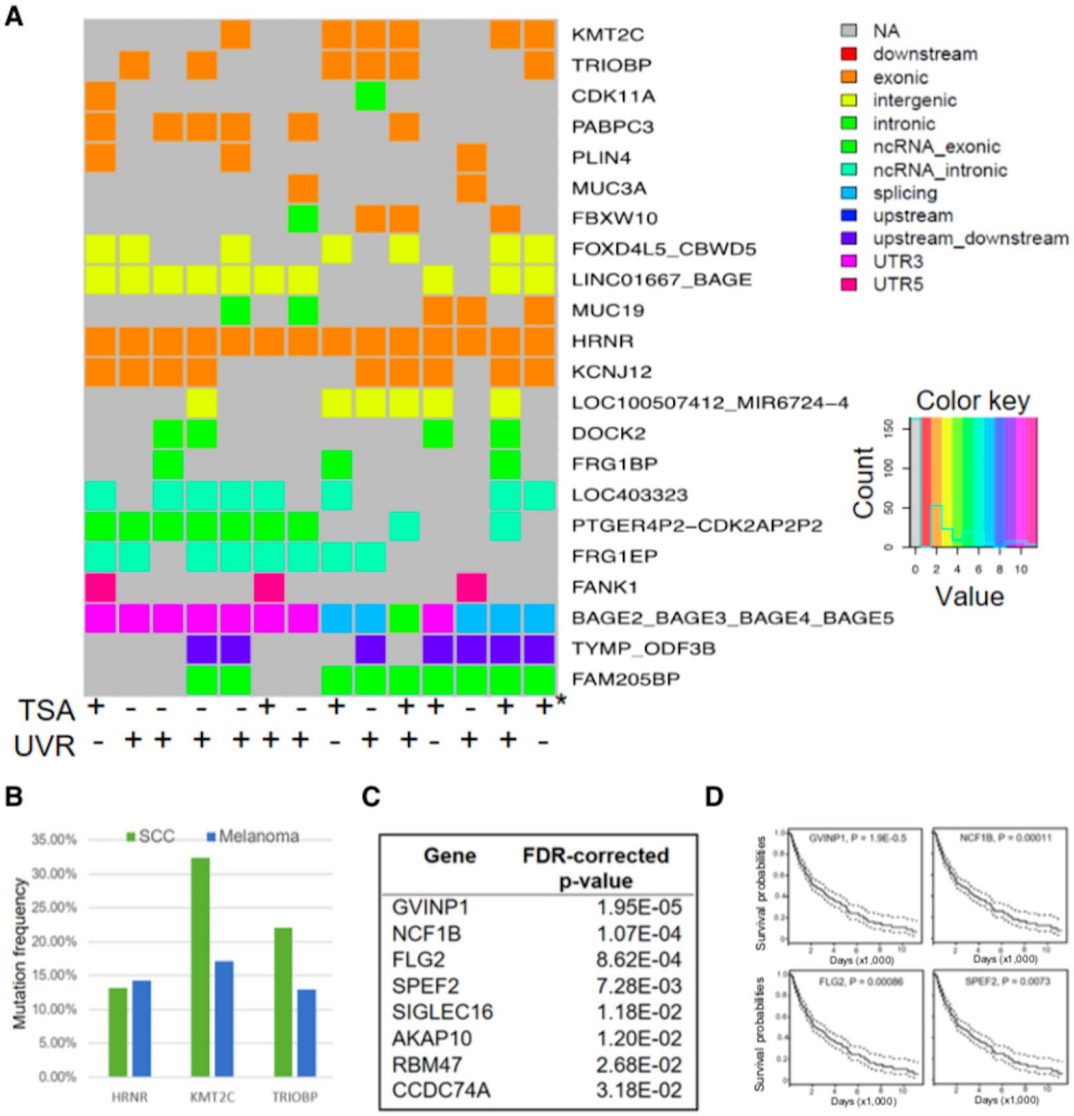
Genes harboring recurrent UVB-induced mutations exist in skin tumor tissues. (**A**) Heat maps showing genes harboring UV- and TSA-induced recurring mutations under different experimental conditions. Genes were included in the heat map if they were mutated in at least three of the 14 experimental conditions. (**B**) Mutation frequency of selected UVB-mutated genes from **4A** in human SCCs and melanomas. (**C**,**D**) UV target genes harboring conserved T > C conversions with significant FDR-corrected p-values (p < 0.05) in the cox proportional hazard test showing a significant association of the activity of these UV target genes with patient survival based on TCGA skin cancer datasets.

Our WES analyses identified exome-wide mutations as a direct result of UVB radiation, whereas previous cancer genomics studies relied on statistical and mathematical modelings to infer founding UV mutations from large complex mutation datasets. By cross comparing the WES datasets from different UV conditions, we isolated genes harboring recurrent mutations and designated them as hotspot UV target genes. In doing so, we found *HRNR, TRIOBP, KCNJ12, KMT2C*, and *PABPC3* genes to be most frequently mutated within their exons, whereas other genes, such as the BAGE family genes, were associated with non-exon mutations (Fig. 4A). To assess whether any of these UV target genes might confer pathogenic potential to skin cancer development, we consulted the skin cancer mutation database in the Catalogue of Somatic Mutations in Cancer (COSMIC) (https://cancer.sanger.ac.uk/cosmic)^42^. We found that *HRNR, KMT2C*, and *PABPC3* were among the most frequently mutated genes in cutaneous SCCs and melanomas (Fig. 4B). *KMT2C*, for example, encodes a histone methyltransferase involved in epigenetic transcriptional regulation. Inactivating KMT2C mutations are linked to aggressive SCCs, and patients with KMT2C mutations often display significantly shorter time to recurrence with higher rates of bone invasion^29^.

In addition to the mutations in genes known to be associated with skin cancer development, we identified multiple new UV target genes that harbor high frequency of T > C conversions, including GVINP1, NCF1B, FLG2, and SPEF2 (Fig. 4C). While the relevance of these new UV target genes in neoplastic transformation and/or cancer progression remains to be elucidated, the activity of these genes appears to be significantly associated with patient survival based on Cox proportional hazard modeling of TCGA skin cancer datasets (FDR-corrected p-value <0.01, Fig. 4D). Notably, T > C conversions in FRG1EP and NBPF1 are highly conserved among the UV-irradiated samples (data not shown). Whether these conserved UV-induced mutations function as founding driver mutations in skin carcinogenesis, however, remains to be experimentally validated in future studies.

**Figure 5:**
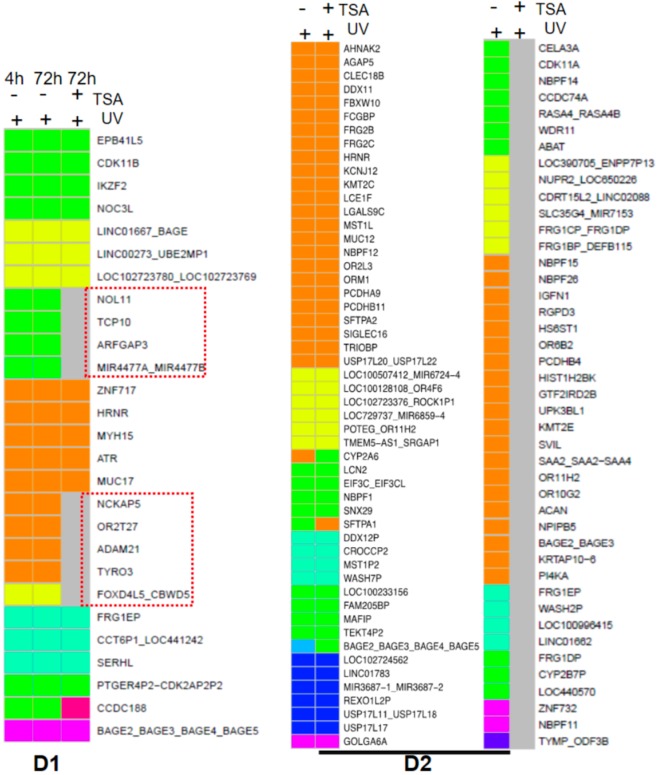
TSA prevented UV-induced mutations. Genes with UVB-induced mutations that were absent in D1 and D2 keratinocytes pretreated with TSA are highlighted in gray in the heat map.

By comparing mutation profiles derived from UVR conditions with or without TSA pretreatment, we found genomic loci in which UVB-induced mutations were prevented by TSA pre-treatment. For example, exonic mutations in *NCKAP5, OR2T27, ADAM21,* and *TRYRO3* in keratinocytes from donor 1 (**D1**) were not detectable if cells were pre-treated with TSA prior to UVB radiation (Fig. 5 left panel). Similar effects were observed in TSA pre-treated D2 keratinocytes (Fig. 5 right panel), highlighting the potentially protective effects of local chromatin remodeling on UV mutagenesis.

## Discussion

This study represents the first comprehensive exome-wide profiling of UV mutations in primary human keratinocytes following acute UVB radiation. There are two major differences between our study and previous UV mutation studies. First, previous studies have relied on targeted sequencing of transgenes or a few selected endogenous genes to characterize UV mutations^12,33,43^. While such methods are sensitive to uncover basic characteristics of UV mutagenesis, they have very limited coverage of the genome, which limited their utility in understanding the complex genome-wide UV mutation features. Our WES-based study, on the other hand, allows unbiased and comprehensive characterization of global UV mutation spectra and types in human keratinocytes following UVB radiation. We chose to focus on the mutagenic effects of acute rather than cumulative UVR as, according to the intermittent exposure hypothesis, it is in fact these short bursts of high-intensity UV exposure that can initiate skin cancer development, especially melanoma^44^. Secondly, recent cancer genomic sequencing studies typically identify thousands of mutations in human skin cancers. These cancer-associated mutations may arise from a combination of UV exposure and other non-UV mutational processes during the long course of cancer evolution. Complex mathematical modeling and statistical tools are needed to indirectly infer UV mutations and to predict which mutations may function as founding driver mutations during skin carcinogenesis. In contrast, our experimental design is unique in that it allows for analysis of direct UV mutations in a biologically relevant model system with minimal influence from other mutational processes.

In addition to the canonical 5′-(C/T)**C**N-3′ sequence context for C > T transition, we found that 5′-A**C**G-3′ is an equally common motif of UVB-induced C > T transition. Furthermore, the T > C transition is the second most prevalent mutation type, accounting for approximately 25% of total UVB mutations with a preferred sequence motif of 5′-A**C**(G/T)-3′. These new UV mutagenic features revealed by our WES analyses have novel implications in understanding the role of UV radiation in skin carcinogenesis. While C > T transition is the most common mutation type following acute UVB radiation, its frequency (approximately 30%) is much lower than that observed in skin cancers (70–90%)^14,29^. The difference can be explained by the experimental focus of our study on UV exposure alone, whereas mutation profiles in skin tumors discovered by cancer genomics reflect long-term cumulative effects from both UV mutagenesis and exposure to other mutagens. In support of this possibility, the UV-induced mutation profile in our study is highly similar to that observed in mouse melanomas induced by a single dose of neonatal UV exposure^45^. Alternatively, the mutation profile in human tumor tissues might represent that of tumor-initiating cells with high C > T mutation content, which undergo clonal expansion during tumor progression. In light of this possibility, precaution should be taken when deducing UV signature mutations based on the mutation log of malignant tissues. It will be interesting to test whether high C > T mutation content promotes tumor growth and becomes selectively enriched in tumor cells.

Our analysis identified several novel genes, including *HRNR, TRIOBP, KCNJ12, KMT2C*, and *PABPC3*, which were mutated under different UVR conditions, suggesting that these genetic loci are highly susceptible to UV-induced photodamage and mutagenesis. Importantly, these genes are often found to be mutated in human SCCs, underscoring their significance as potential founding mutations during UV-induced skin carcinogenesis. It is worth noting, however, that some commonly mutated genes in skin cancers, such as *TP53, NOTCH*, and *PTCH1*, do not appear in the mutated gene list following acute UVB radiation. It is possible that the mutation frequency of these key cancer genes is below the detection limit of our WES studies. Given that most non-melanoma skin cancers arise after decades of chronic sun damage, mutations affecting key cancer genes likely occur stochastically to confer growth advantage and allow for clonal expansion of cells harboring key cancer mutations thereby increasing their frequency in tumor tissues. Our experimental design utilized UVB alone in an acute setting, which is similar to studies showing that mutation profiles of lung cancer from heavy smokers differ significantly from BaP-induced mutations in cultured lung cells^39^. In light of multiple recent studies illustrating that normal appearing tissues harbor mutated cancer genes similar to that seen in cancerous tissues^46,47^, the presence of cancer-associated mutations in UV-treated cells is insufficient to predict the malignant status of these cells. Indeed, in our experience, it is difficult to immortalize or transform primary keratinocytes in cell culture following repetitive exposure to different doses of UVB radiation (unpublished observations).

Importantly, WES has the ability to produce high-quality sequence reads not only from the typical coding regions, but also from noncoding regions, like introns, intergenic regions, and untranslatable regions (UTRs)^48,49^. WES datasets from our study found that about 30% of UV-induced SNVs were positioned in gene bodies (introns and exons), but notably greater than 50% were located in intergenic regions (Fig. 1A). Multiple studies have demonstrated that the non-coding portions of the genome in fact house the bulk of mutations, somatic or germline. The role of UV-induced mutations in non-coding regions during skin carcinogenesis remain to be defined. We included TSA in the experiments to test how the chromatin environment affects UV-induced DNA repair and mutagenesis. Unexpectedly, we found a substantial amount of SNVs in TSA-treated cells, suggesting that TSA itself as a mutagen. Broad-spectrum HDACis such as vorinostat, a TSA analog, have been approved for treatment of human malignancies^50^. While these agents exhibit effective anti-cancer properties, their therapeutic effect and mechanism(s) of action against tumor cells are believed to be due to epigenetic control of the genes involved in cell proliferation, cell cycle regulation, and apoptosis^50^. Based on structural similarities between TSA and SAHA, our serendipitous discovery of TSA and SAHA as DNA-damaging agents provides new insights into their anti-cancer effects, while also raising concerns regarding the safety features of such epigenetic drugs due to potential genotoxic effects that remain to be to further investigated. It is interesting to note that the mutational profiles between TSA and UVB are largely similar, except that TSA-induced mutations are mainly located in gene bodies (Fig. 1D).

In summary, our WES studies not only demonstrate UVB-induced mutation features characteristic of UV signature mutations as described previously, such as the dominant C > T mutation type and its preferred sequence context, but also uncover novel UV mutational features at endogenous chromosomal loci across the genome. These new findings will enhance our understanding of the UV mutational process and its impact on skin homeostasis and disease pathogenesis via its genotoxic effects. Identification of new UV target genes that are frequently mutated in response to acute UV radiation has important ramifications for deciphering UV-induced founding mutations to better understand the evolution of the complex mutation profiles associated with skin cancer development and progression.

## Materials and Methods

### Cells and reagents

Primary human keratinocytes from neonatal foreskins were obtained from the Columbia University Skin Disease Research Center (epiCURE) Tissue Culture Core facility^51,52^. The epiCURE collected neonatal foreskins from healthy newborns with informed consent from a parent and/or legal guardian for study participation through the Columbia University Children’s Hospital under a protocol (# AAAD6866) that was approved by the Institutional Review Board at Columbia University Medical Center. All samples were de-identified prior to being received by researchers and designated as non-human subject research under 45 CFR Part 46. All experiments were performed in accordance with the relevant guidelines and regulations. Cells were cultured in CnT keratinocyte medium supplemented with human keratinocyte growth supplement (ZenBio, Research Triangle Park, NC). Etoposide, TSA, and suberoylanilide hydroxamic acid (SAHA) were purchased from Sigma-Aldrich (St. Louis, MO) and were dissolved in DMSO at a final concentration of 10 mM as a stock solution. The working concentration for etoposide was 50 µM, for TSA was 200 nM and for SAHA was 5 µM throughout this study^53^. UVB radiation was supplied by 4 FS20T12/UVB tubes (National Biological Corp., Beachwood, OH), which emit UV rays between 290 and 320 nm with an emission peak at 313 nm^51,54^. UVR dose was measured using an IL1700 radiometer and a SED240 UVB detector (International Light, Newburyport, MA) at a distance of 27 cm from the UV source to the cell culture dishes. Cells were irradiated with different doses of UVR (10, 20, 30, or 40 mJ/cm^2^) as indicated and then collected 4 or 72 h after UV exposure. Non-irradiated control cells from each donor were maintained in identical culturing conditions and used in all experiments. Sequencing reads from each UV-irradiated or TSA-treated cells were compared against the reads from the non-irradiated control cells of the same donor to eliminate false mutations due to either polymorphism or background mutations.

### Reactive oxidative stress (ROS) measurement and DNA comet assay

For ROS measurement, primary human keratinocytes were treated with TSA (200 nM), SAHA (50 µM), DMSO, or H_2_O_2_ (100 µM), respectively, for 1 h. Treated cells were harvested using trypsin-EDTA 0.05% (Gibco), washed 2x with PBS, resuspended in 100 µl of ROS assay staining solution (Invitrogen), and incubated at 37 °C with 5% CO2 for 1 h. After incubation with the staining solution, culture media containing TSA, SAHA, DMASO, or H_2_O_2_ were added into each sample and incubated at 37 °C with 5% CO2 for another hour. Cells were then analyzed by BD LSRFortessa (BD Biosciences) at 520 nm to detect ROS generation. Results were analyzed using FlowJo® (BD Biosciences). For the comet assay, primary human keratinocytes were treated with TSA, SAHA, etoposide (positive control), or DMSO for 4 h or 24 h. Following treatment, cells were harvested and washed with ice-cold PBS twice. The neutral comet assay was performed using a Comet Assay® Kit (Trevigen) following manufacturer’s instructions. Briefly, cells were mixed with low melting agarose at 37 °C. The cells and agarose mixture were added to the comet slide and incubated at 4 °C for 30 minutes in the dark to improve the gel adherence. The slides were immersed in cold lysis solution for 1 h and then incubated in 4 °C of neutral tris-acetate buffer for 30 minutes. The slide was electrophoresed at 35 volts for 30 minutes. After incubation with DNA precipitation solution (1 M NH4Ac in 95% ethanol) for 30 minutes, the slides were immersed in 70% ethanol for 30 minutes at room temperature. Samples were dried at 37 °C for 15 minutes and DNA was stained using SYBR Safe DNA Gel stain (APExBio) following manufacturer’s instructions. Slides were viewed using Apotome microscopy (Zeiss) to image DNA double-strand breaks.

### DNA preparation and WES analysis

Genomic DNA was isolated from UVB-irradiated and control keratinocytes using the Wizard Genomic DNA Purification Kit (Promega). WES was performed at the Columbia Genome Center following a standard Illumina TruSeq multiplexing protocol to generate a targeted number of reads with greater than 85% coverage of the targeted regions by ≥15 reads and 90% covered by ≥10 reads. All FASTQ files were analyzed with FastQC to ensure sample homogeneity and quality. FASTQ sequences were then aligned with the human GRCh38 reference genome using Burrows-Wheeler Aligner (BWA-MEM, version 0.7.15) with default settings. Resulting SAM files were converted and compressed to BAM format using the Samtools program^55^, which were then sorted and indexed using Picard-tools (version 1.141) (http://broadinstitute.github.io/picard). Duplicate reads were marked using Picard-tools. Variant calling was performed following Genome Analysis Toolkits (GATK) best practices^56^. Local realignment around indels was performed in two steps: creation of a table of possible indels using GATK (version 3.5) RealignerTargetCreator followed by realignment of reads around those targets with GATK IndelRealigner. The base quality score recalibration required two steps: generation of a recalibration table with GATK BaseRecalibrator and printing reads based on the previous table with GATK PrintReads. An average of 53.9 million reads were sequenced per sample, of which 99.6% were mapped to the genome, and 83.3% were on target with a median coverage of 62 (See Supplementary Table 1 for summary of statistics). Recalibrated BAM files were used to call variants with MuTect2 software^57^ using default parameters (read quality>20) and a contamination fraction set at 0.01. As MuTect2 is tuned to perform normal/tumor comparison, reads from control keratinocytes were used as “normal” samples and UVR or TSA-treated cells as “tumor” samples. We removed identified variants that are known polymorphisms in multiple databases, e.g., the dbSNP146 database and the Catalogue of Somatic Mutations in Cancer (COSMIC). We then used VarScan v2.3.9^58^ to further filter out somatic mutations whose coverage was fewer than 6, with fewer than 2 non-reference bases. We kept single nucleotide variations (SNVs) with mutational frequency higher than 10% for subsequent mutation analyses.

### Annotations of mutation data

For all datasets, chromosome number, genomic coordinates, and reference and mutated nucleotides were extracted for each variant. Variants were annotated with AnnoVar (version 2017July17)^59^ using databases such as refGene, cosmic70, exact03, avsnp150, dbnsfp33a, and cytoBand for the hg38 human genome build. We included mutations in the analyses only if they could be successfully annotated. Lists of all somatic mutations identified in the samples are provided in Supplementary Table 2. For analyses of mutation signatures, mutations were classified into six types determined by the six possible substitutions (A:T > C:G, A:T > G:C, A:T > T:A, C:G > A:T, C:G > G:C, C:G > T:A) and the 16 combinations of flanking (5′ and 3′) nucleotides.

### Functional annotation analysis

A comprehensive list of established cancer driver genes (oncogenes and tumor suppressor genes) was assembled from the literature and somatic mutation database mining^42,60,61^. The comprehensive list of genes mutated in melanoma or cutaneous squamous cell carcinomas (SCCs) was compared with genes identified as harboring recurring UVB-induced somatic mutations in our samples.

### Statistical analyses

One-Way ANOVA test was performed for statistical analysis of the comet assay data using GraphPad Prism. Statistical analyses of the WES datasets were performed using R software (R Core Team, 2017, v3.4.0). Since identification of a mutation depends on the coverage (number of reads) mapped to the specific genomic position and follows a binomial distribution, we first normalized all samples based on their coverage to compare the number of somatic mutations among different samples with different coverage. To do this, we first used “bedtools coverage” to estimate the read coverage in the target regions defined by the Agilent SureSelect Human All Exon V6 + UTR covered genomic regions, e.g., bedfiles. We then calculated coverage for each genomic position using length of reads multiplied by the number of reads divided by the genomic interval length (Supplementary Fig. 3 for coverage distribution). Then, we applied a method similar to the size factor estimation algorithm in DESeq. 2^62^ to normalize each sample read and coverage. Specifically, we first obtained the geometric mean of the estimated read coverage for each target interval across all samples. Then, in each sample, we computed the ratio of coverage by comparing the estimated read coverage in that sample versus the computed geometric mean for each target interval. Finally, we took the average of all ratios across all intervals in each sample to obtain the size factor for that specific sample. A sample with a larger size factor is expected to have more somatic mutations. The number of total somatic reads in each sample was then normalized to the corresponding size factor to correct for sequencing depth in each experiment.

## Supplementary information

Supplementary Information.

## Data Availability

The datasets generated during the current study are available from the corresponding author on reasonable request.
