# Supplementary material for: Exome sequencing identifies novel mutation signatures of UV radiation and trichostatin A in primary human keratinocytes
Source: Sci Rep. 2020 Mar 18;10:4943. doi: 10.1038/s41598-020-61807-4 (PMC7080724; doi:10.1038/s41598-020-61807-4)
Supplement: Supplementary file 1 — Supplementary Information. [file 41598_2020_61807_MOESM1_ESM.pdf]

Supplementary Information

Exome sequencing identifies novel mutation signatures of UV radiation and trichostatin A in primary human keratinocytes

Yao Shen[1], Wootae Ha[2], Wangyong Zeng[3], Dawn Queen[4], and Liang Liu[2,3] *

[1]Department of Systems Biology, Columbia University, New York, New York

[2]The Hormel Institute, University of Minnesota, Austin, MN

[3]Department of Dermatology, Columbia University, New York, NY

[4]Columbia University Vagelos College of Physicians and Surgeons, New York, NY 10032

[*]To whom correspondence should be addressed:

The Hormel Institute

University of Minnesota

Austin, MN

55912

Phone: 507-437-9627

Email: LIU00965@umn.edu

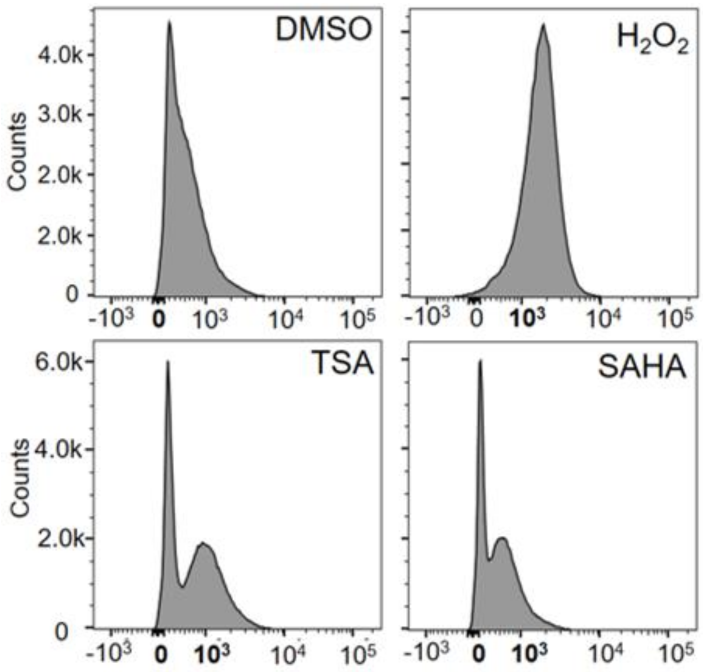

Supplementary Figure 1. TSA and SAHA increased ROS levels in primary human keratinocytes. H_2O_2 treatment was included as a positive control.

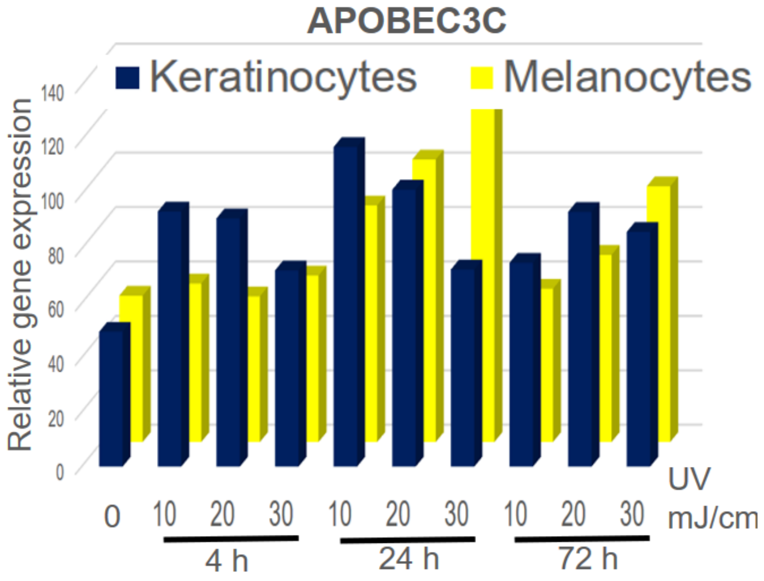

Supplementary Figure 2. Changes in APOBEC3C mRNA expression in melanocytes and keratinocytes following acute UVB exposure.

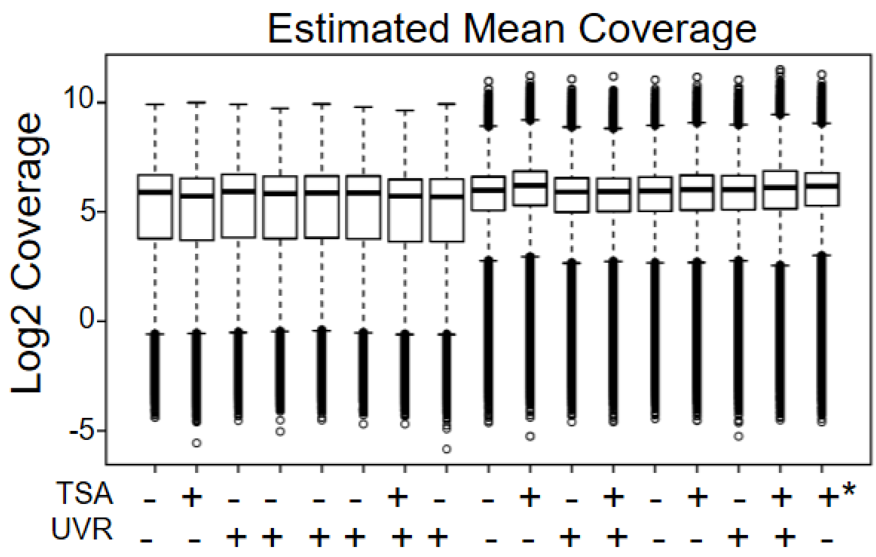

Supplementary Figure 3. Schematic illustration of coverage distribution for each sample. Coverage for each genomic position was calculated by using the length of reads multiplied by the number of reads, then divided by the genomic interval length.

Supplementary Table 1. Summary of statistics of the sequencing reads for all experimental samples included in this study. An average of 53.9 million reads per sample, of which 99.6% were mapped to the genome and 83.3% were on target with a median coverage of 62, were generated from WES.

Supplementary Table 2. Lists of somatic mutations identified in the experimental samples that were used for analyses of mutation signatures. We included mutations in the analyses only if they could be successfully annotated.

Supplementary Table 1

Sample	TotalReads	MapTargetReads	MapTargetRatio	MedianCoverage
LL001	65660860	52480207	0.799261645	60.6
LL002	57544332	47710854	0.829114742	53.025
LL003	67809916	53467359	0.788488796	61.94666667
LL004	63837317	49820073	0.780422413	57.71428571
LL005	65453708	51003393	0.779228474	59.04615385
LL006	64819207	50755135	0.783026164	58.83164983
LL007	56890045	45296905	0.796218477	52.98360656
LL008	56741235	45960293	0.809998108	51.60262009
s1con	43330473	37714136	0.870383667	64.35563718
s2TSA	51901883	45253836	0.871911256	74.60732984
s3UV	41723414	36271406	0.869329772	60.8193795
s4TSA_UV	41302101	35624309	0.862530189	61.81818182
s5con	43513770	37828803	0.86935246	62.8113741
s6TSA	46897679	40158161	0.856293144	65.05102041
s7UV	45869550	39332123	0.857477848	65.67164179
s8TSA_UV	52787917	46535226	0.881550715	69.57831325
s9TSA2x	49452523	42731833	0.864098137	72.85714286
Mean	53855055	44584944	0.833	62
SD	9282462.527	5901331.901	0.03877603	6.488909918

Supplementary Table 2

Sample	Chr	Start	Ref	Alt	Func.refGene	Gene.refGene	ExonicFunc.refGene
LL002	chr1	1657358	-	A	intronic	CDK11B	.
LL002	chr1	1703565	T	C	exonic	CDK11A	synonymous_SNV
LL002	chr1	125180202	C	G	intergenic	NONE_NONE	.
LL002	chr1	125180250	A	T	intergenic	NONE_NONE	.
LL002	chr1	125180336	G	C	intergenic	NONE_NONE	.
LL002	chr1	143214671	T	G	intergenic	NONE_LOC645166	.
LL002	chr1	143214750	A	T	intergenic	NONE_LOC645166	.
LL002	chr1	148952882	G	A	exonic	PDE4DIP	synonymous_SNV
LL002	chr1	152215165	G	A	exonic	HRNR	nonsynonymous_SNV
LL002	chr3	75738643	A	G	exonic	ZNF717	nonsynonymous_SNV
LL002	chr3	75738661	-	T	exonic	ZNF717	frameshift_insertion
LL002	chr3	130471877	T	-	exonic	COL6A5	frameshift_deletion
LL002	chr4	53416	C	T	UTR5	ZNF595	.
LL002	chr7	100752368	T	C	exonic	ZAN	nonsynonymous_SNV
LL002	chr7	101037930	G	C	exonic	MUC17	nonsynonymous_SNV
LL002	chr9	62844751	C	T	ncRNA_exonic	PTGER4P2-CDK2AP2P2	.
LL002	chr9	62844772	C	T	ncRNA_exonic	PTGER4P2-CDK2AP2P2	.
LL002	chr9	62844842	G	T	ncRNA_exonic	PTGER4P2-CDK2AP2P2	.
LL002	chr9	62858319	G	A	ncRNA_intronic	LOC403323	.
LL002	chr9	63819568	A	G	upstream_downstream	MIR4477B_LINC00537_MIR4477A	.
LL002	chr9	63819584	G	A	ncRNA_exonic	MIR4477A_MIR4477B	.
LL002	chr9	65646931	A	T	intergenic	FOXD4L5_CBWD5	.
LL002	chr9	65650190	G	T	intergenic	FOXD4L5_CBWD5	.
LL002	chr10	125896581	C	A	UTR5	FANK1	.
LL002	chr13	25096712	C	T	exonic	PABPC3	stopgain
LL002	chr16	34582987	G	C	intergenic	LINC00273_UBE2MP1	.
LL002	chr16	34583017	C	G	intergenic	LINC00273_UBE2MP1	.
LL002	chr16	34583045	G	A	intergenic	LINC00273_UBE2MP1	.
LL002	chr16	34583092	A	C	intergenic	LINC00273_UBE2MP1	.
LL002	chr16	46388671	T	A	intergenic	NONE_ANKRD26P1	.
LL002	chr16	46390265	G	T	intergenic	NONE_ANKRD26P1	.
LL002	chr16	46394682	T	C	intergenic	NONE_ANKRD26P1	.
LL002	chr16	46394751	T	A	intergenic	NONE_ANKRD26P1	.
LL002	chr16	46400864	C	T	intergenic	NONE_ANKRD26P1	.
LL002	chr16	46401096	C	T	intergenic	NONE_ANKRD26P1	.
LL002	chr17	21415939	C	T	exonic	KCNJ12	synonymous_SNV
LL002	chr19	4511317	CTGGACGGCCCCTTTGC	-	exonic	PLIN4	nonframeshift_deletion
LL002	chr20	29496310	T	C	ncRNA_intronic	FRG1EP	.
LL002	chr21	10397846	C	T	intergenic	LINC01667_BAGE	.
LL002	chr21	10473155	T	C	UTR3	BAGE2_BAGE3_BAGE4_BAGE5	.
LL002	chr21	10473159	G	A	UTR3	BAGE2_BAGE3_BAGE4_BAGE5	.

LL002	chr22	11279319	G	A	intergenic	LOC102723780_LOC102723769	.
LL002	chr22	11279338	G	A	intergenic	LOC102723780_LOC102723769	.
LL002	chr22	11279365	C	G	intergenic	LOC102723780_LOC102723769	.
LL002	chrX	74591860	A	G	exonic	RLIM	synonymous_SNV
LL002	chrX	74591902	T	A	exonic	RLIM	synonymous_SNV
LL002	chrX	74591904	A	G	exonic	RLIM	nonsynonymous_SNV
LL002	chrX	74591920	C	G	exonic	RLIM	synonymous_SNV
LL002	chrX	74591926	G	T	exonic	RLIM	synonymous_SNV
LL002	chrM	709	G	A	ncRNA_exonic	RNR1	.
LL002	chrM	15452	C	A	intergenic	RNR2_NONE	.
LL002	chrM	15607	A	G	intergenic	RNR2_NONE	.
LL002	chr9_KI270720v1_random	5118	G	A	intergenic	NONE_NONE	.
LL002	chr9_KI270720v1_random	5153	T	C	intergenic	NONE_NONE	.
LL002	chr9_KI270720v1_random	5162	G	C	intergenic	NONE_NONE	.
LL002	chr17_GL000205v2_random	51415	A	G	intergenic	NONE_MGC70870	.
LL002	chr17_GL000205v2_random	51451	T	C	intergenic	NONE_MGC70870	.
LL002	chrUn_KI270438v1	104006	A	G	intergenic	NONE_NONE	.
LL002	chrUn_KI270438v1	104059	G	C	intergenic	NONE_NONE	.
LL002	chrUn_KI270438v1	104060	A	G	intergenic	NONE_NONE	.
LL002	chrUn_KI270438v1	109252	G	C	intergenic	NONE_NONE	.
LL002	chrUn_KI270438v1	109509	A	G	intergenic	NONE_NONE	.
LL002	chrUn_KI270438v1	109521	T	G	intergenic	NONE_NONE	.
LL002	chrUn_KI270438v1	109602	C	T	intergenic	NONE_NONE	.
LL002	chrUn_KI270438v1	109632	CGAATGGAATGGAAG	-	intergenic	NONE_NONE	.
LL002	chrUn_KI270438v1	109638	GAATGGAAGC	-	intergenic	NONE_NONE	.
LL002	chrUn_KI270438v1	109643	GAAGC	-	intergenic	NONE_NONE	.
LL002	chrUn_KI270438v1	109659	AATGGAATGA	-	intergenic	NONE_NONE	.
LL002	chrUn_KI270438v1	109664	AATGA	-	intergenic	NONE_NONE	.
LL002	chrUn_KI270438v1	109813	G	A	intergenic	NONE_NONE	.
LL002	chrUn_KI270438v1	109844	A	T	intergenic	NONE_NONE	.
LL002	chrUn_KI270438v1	109873	G	A	intergenic	NONE_NONE	.
LL002	chrUn_KI270438v1	109945	C	T	intergenic	NONE_NONE	.
LL002	chrUn_KI270438v1	109996	T	A	intergenic	NONE_NONE	.
LL002	chrUn_KI270438v1	112173	T	C	intergenic	NONE_NONE	.
LL002	chrUn_KI270438v1	112270	GAAT	-	intergenic	NONE_NONE	.
LL002	chrUn_KI270438v1	112275	GAATGGAATGA	-	intergenic	NONE_NONE	.
LL002	chrUn_KI270438v1	112319	G	C	intergenic	NONE_NONE	.
LL002	chrUn_KI270438v1	112339	T	G	intergenic	NONE_NONE	.
LL002	chrUn_KI270438v1	112340	G	T	intergenic	NONE_NONE	.
Sample	Chr	Start	Ref	Alt	Func.refGene	Gene.refGene	ExonicFunc.refGene
LL003	chr1	1647255	T	C	intronic	CDK11B	.
LL003	chr1	15716230	C	-	intronic	PLEKHM2	.

LL003	chr1	125180250	A	T	intergenic	NONE_NONE	.
LL003	chr1	143214750	A	T	intergenic	NONE_LOC645166	.
LL003	chr1	152215165	G	A	exonic	HRNR	nonsynonymous_SNV
LL003	chr1	161630399	A	C	intronic	FCGR3B	.
LL003	chr1	248650825	G	A	exonic	OR2T27	synonymous_SNV
LL003	chr2	120131597	G	C	intronic	EPB41L5	.
LL003	chr2	131444045	T	C	ncRNA_exonic	NOC2LP2	.
LL003	chr2	132781993	G	A	exonic	NCKAP5	synonymous_SNV
LL003	chr2	213147680	AA	-	intronic	IKZF2	.
LL003	chr3	75737447	G	A	exonic	ZNF717	nonsynonymous_SNV
LL003	chr3	75738643	A	G	exonic	ZNF717	nonsynonymous_SNV
LL003	chr3	75738661	-	T	exonic	ZNF717	frameshift_insertion
LL003	chr3	93470578	C	T	intergenic	NONE_PROS1	.
LL003	chr3	93470585	A	T	intergenic	NONE_PROS1	.
LL003	chr3	93470598	A	T	intergenic	NONE_PROS1	.
LL003	chr3	93470610	A	C	intergenic	NONE_PROS1	.
LL003	chr3	93470667	G	A	intergenic	NONE_PROS1	.
LL003	chr3	93470698	A	G	intergenic	NONE_PROS1	.
LL003	chr3	93470734	T	C	intergenic	NONE_PROS1	.
LL003	chr3	108470146	G	A	exonic	MYH15	nonsynonymous_SNV
LL003	chr3	142558694	A	G	exonic	ATR	synonymous_SNV
LL003	chr4	53416	C	T	UTR5	ZNF595	.
LL003	chr4	3589151	C	T	ncRNA_exonic	LINC00955	.
LL003	chr4	73483947	T	G	exonic	AFM	nonsynonymous_SNV
LL003	chr4	109685238	-	T	intronic	MCUB	.
LL003	chr4	121037032	A	G	exonic	NDNF	synonymous_SNV
LL003	chr4	122743272	G	C	exonic	BBS12	synonymous_SNV
LL003	chr6	10633809	T	G	intergenic	GCNT2_C6orf52	.
LL003	chr6	167375916	C	G	intronic	TCP10	.
LL003	chr7	65762926	T	C	ncRNA_intronic	CCT6P1_LOC441242	.
LL003	chr7	99768320	C	A	intronic	CYP3A4	.
LL003	chr7	101032695	G	A	exonic	MUC17	nonsynonymous_SNV
LL003	chr7	101034678	G	T	exonic	MUC17	nonsynonymous_SNV
LL003	chr7	101037930	G	C	exonic	MUC17	nonsynonymous_SNV
LL003	chr7	142469894	C	T	intergenic	TRY2P_MTRNR2L6	.
LL003	chr9	62844751	C	T	ncRNA_exonic	PTGER4P2-CDK2AP2P2	.
LL003	chr9	62844772	C	T	ncRNA_exonic	PTGER4P2-CDK2AP2P2	.
LL003	chr9	63819584	G	A	ncRNA_exonic	MIR4477A_MIR4477B	.
LL003	chr9	65650190	G	T	intergenic	FOXD4L5_CBWD5	.
LL003	chr10	94358222	CA	-	intronic	NOC3L	.
LL003	chr11	6716748	T	C	ncRNA_exonic	GVINP1	.
LL003	chr11	47638786	G	A	exonic	MTCH2	synonymous_SNV

LL003	chr11	64116555	C	T	exonic	FLRT1	synonymous_SNV
LL003	chr11	133458887	G	A	intronic	OPCML	.
LL003	chr12	121240538	G	T	exonic	CAMKK2	nonsynonymous_SNV
LL003	chr14	70457733	C	T	exonic	ADAM21	synonymous_SNV
LL003	chr15	41573327	G	T	exonic	TYRO3	nonsynonymous_SNV
LL003	chr15	64126022	C	G	intronic	SNX1	.
LL003	chr16	34574039	C	T	intergenic	LINC00273_UBE2MP1	.
LL003	chr16	34583017	C	G	intergenic	LINC00273_UBE2MP1	.
LL003	chr16	34583045	G	A	intergenic	LINC00273_UBE2MP1	.
LL003	chr16	34583092	A	C	intergenic	LINC00273_UBE2MP1	.
LL003	chr16	34588255	G	C	intergenic	LINC00273_UBE2MP1	.
LL003	chr16	34588260	A	T	intergenic	LINC00273_UBE2MP1	.
LL003	chr16	46388667	ATCATCGAATGAGATCGA	-	intergenic	NONE_ANKRD26P1	.
LL003	chr16	46388671	T	A	intergenic	NONE_ANKRD26P1	.
LL003	chr16	46388753	T	A	intergenic	NONE_ANKRD26P1	.
LL003	chr16	46390081	C	G	intergenic	NONE_ANKRD26P1	.
LL003	chr16	46390265	G	T	intergenic	NONE_ANKRD26P1	.
LL003	chr16	46390369	T	A	intergenic	NONE_ANKRD26P1	.
LL003	chr16	46390581	G	C	intergenic	NONE_ANKRD26P1	.
LL003	chr16	46390643	T	A	intergenic	NONE_ANKRD26P1	.
LL003	chr16	46390698	G	A	intergenic	NONE_ANKRD26P1	.
LL003	chr16	46390759	G	A	intergenic	NONE_ANKRD26P1	.
LL003	chr16	46394682	T	C	intergenic	NONE_ANKRD26P1	.
LL003	chr16	46394756	T	A	intergenic	NONE_ANKRD26P1	.
LL003	chr16	46399271	G	C	intergenic	NONE_ANKRD26P1	.
LL003	chr16	46400864	C	T	intergenic	NONE_ANKRD26P1	.
LL003	chr16	46400868	A	T	intergenic	NONE_ANKRD26P1	.
LL003	chr17	21415939	C	T	exonic	KCNJ12	synonymous_SNV
LL003	chr17	47974545	G	A	UTR5	CDK5RAP3	.
LL003	chr17	50740237	-	T	intronic	LUC7L3	.
LL003	chr17	67743673	A	G	intronic	NOL11	.
LL003	chr19	35716434	G	C	intronic	ZBTB32	.
LL003	chr20	28565360	C	T	intergenic	NONE_FRG1CP	.
LL003	chr20	29496263	A	G	ncRNA_intronic	FRG1EP	.
LL003	chr20	29496310	T	C	ncRNA_intronic	FRG1EP	.
LL003	chr21	10397775	G	C	intergenic	LINC01667_BAGE	.
LL003	chr21	10397846	C	T	intergenic	LINC01667_BAGE	.
LL003	chr21	10473155	T	C	UTR3	BAGE2_BAGE3_BAGE4_BAGE5	.
LL003	chr21	10473159	G	A	UTR3	BAGE2_BAGE3_BAGE4_BAGE5	.
LL003	chr22	11279319	G	A	intergenic	LOC102723780_LOC102723769	.
LL003	chr22	11279338	G	A	intergenic	LOC102723780_LOC102723769	.
LL003	chr22	11279365	C	G	intergenic	LOC102723780_LOC102723769	.

LL003	chr22	20149712	C	T	intronic	CCDC188	.
LL003	chr22	20149762	G	A	UTR5	CCDC188	.
LL003	chr22	37725145	C	A	exonic	TRIOBP	nonsynonymous_SNV
LL003	chr22	42509113	G	A	ncRNA_intronic	SERHL	.
LL003	chr22	42817625	-	A	intronic	ARFGAP3	.
LL003	chrY	56763492	GAATGGAAAGGAATT	-	intergenic	NONE_SPRY3	.
LL003	chrM	15452	C	A	intergenic	RNR2_NONE	.
LL003	chr9_KI270720v1_random	5118	G	A	intergenic	NONE_NONE	.
LL003	chr9_KI270720v1_random	5153	T	C	intergenic	NONE_NONE	.
LL003	chr9_KI270720v1_random	5162	G	C	intergenic	NONE_NONE	.
LL003	chr17_GL000205v2_random	51379	G	A	intergenic	NONE_MGC70870	.
LL003	chr17_GL000205v2_random	51413	G	A	intergenic	NONE_MGC70870	.
LL003	chr17_GL000205v2_random	51415	A	G	intergenic	NONE_MGC70870	.
LL003	chr17_GL000205v2_random	51437	G	A	intergenic	NONE_MGC70870	.
LL003	chrUn_KI270438v1	104059	G	C	intergenic	NONE_NONE	.
LL003	chrUn_KI270438v1	104060	A	G	intergenic	NONE_NONE	.
LL003	chrUn_KI270438v1	104093	G	A	intergenic	NONE_NONE	.
LL003	chrUn_KI270438v1	104121	A	G	intergenic	NONE_NONE	.
LL003	chrUn_KI270438v1	104136	A	C	intergenic	NONE_NONE	.
LL003	chrUn_KI270438v1	104157	C	T	intergenic	NONE_NONE	.
LL003	chrUn_KI270438v1	104335	AATGGAATGGAATCG	-	intergenic	NONE_NONE	.
LL003	chrUn_KI270438v1	109333	G	A	intergenic	NONE_NONE	.
LL003	chrUn_KI270438v1	109376	T	C	intergenic	NONE_NONE	.
LL003	chrUn_KI270438v1	109423	G	C	intergenic	NONE_NONE	.
LL003	chrUn_KI270438v1	109424	-	AT	intergenic	NONE_NONE	.
LL003	chrUn_KI270438v1	109521	T	G	intergenic	NONE_NONE	.
LL003	chrUn_KI270438v1	109632	CGAATGGAATGGAAG	-	intergenic	NONE_NONE	.
LL003	chrUn_KI270438v1	109659	AATGGAATGA	-	intergenic	NONE_NONE	.
LL003	chrUn_KI270438v1	109664	AATGA	-	intergenic	NONE_NONE	.
LL003	chrUn_KI270438v1	109693	G	A	intergenic	NONE_NONE	.
LL003	chrUn_KI270438v1	109766	A	T	intergenic	NONE_NONE	.
LL003	chrUn_KI270438v1	109813	G	A	intergenic	NONE_NONE	.
LL003	chrUn_KI270438v1	109945	C	T	intergenic	NONE_NONE	.
LL003	chrUn_KI270438v1	110470	A	T	intergenic	NONE_NONE	.
LL003	chrUn_KI270438v1	110486	C	T	intergenic	NONE_NONE	.
LL003	chrUn_KI270438v1	110487	G	A	intergenic	NONE_NONE	.
LL003	chrUn_KI270438v1	110525	C	T	intergenic	NONE_NONE	.
LL003	chrUn_KI270438v1	112173	T	C	intergenic	NONE_NONE	.
LL003	chrUn_KI270438v1	112319	G	C	intergenic	NONE_NONE	.
Sample	Chr	Start	Ref	Alt	Func.refGene	Gene.refGene	ExonicFunc.refGene
LL004	chr1	1657358	-	A	intronic	CDK11B	.
LL004	chr1	1668501	C	T	intronic	SLC35E2B	.

LL004	chr1	8951478	G	A	intronic	CA6	.
LL004	chr1	15397347	G	A	exonic	FHAD1	synonymous_SNV
LL004	chr1	40991151	C	T	intronic	CTPS1	.
LL004	chr1	53910028	C	T	UTR3	DIO1	.
LL004	chr1	125180202	C	G	intergenic	NONE_NONE	.
LL004	chr1	125180232	C	T	intergenic	NONE_NONE	.
LL004	chr1	125180250	A	T	intergenic	NONE_NONE	.
LL004	chr1	148952882	G	A	exonic	PDE4DIP	synonymous_SNV
LL004	chr1	148953097	C	T	exonic	PDE4DIP	nonsynonymous_SNV
LL004	chr1	152215165	G	A	exonic	HRNR	nonsynonymous_SNV
LL004	chr1	152216522	C	T	exonic	HRNR	nonsynonymous_SNV
LL004	chr1	155763594	T	C	intronic	GON4L	.
LL004	chr1	171557431	G	A	exonic	PRRC2C	synonymous_SNV
LL004	chr1	207062609	G	A	intronic	PFKFB2	.
LL004	chr1	233353960	TG	-	intronic	MAP3K21	.
LL004	chr1	248650825	G	A	exonic	OR2T27	synonymous_SNV
LL004	chr2	132781887	A	T	intronic	NCKAP5	.
LL004	chr2	213147680	AA	-	intronic	IKZF2	.
LL004	chr3	10101265	C	T	UTR3	FANCD2	.
LL004	chr3	15042828	T	G	intronic	NR2C2	.
LL004	chr3	52576635	T	A	exonic	PBRM1	synonymous_SNV
LL004	chr3	93470593	A	G	intergenic	NONE_PROS1	.
LL004	chr3	93470653	A	T	intergenic	NONE_PROS1	.
LL004	chr3	93470734	T	C	intergenic	NONE_PROS1	.
LL004	chr3	142558694	A	G	exonic	ATR	synonymous_SNV
LL004	chr4	40438198	T	C	exonic	RBM47	synonymous_SNV
LL004	chr5	170080127	TG	-	intronic	DOCK2	.
LL004	chr7	65762926	T	C	ncRNA_intronic	CCT6P1_LOC441242	.
LL004	chr7	99768320	C	A	intronic	CYP3A4	.
LL004	chr7	101034678	G	T	exonic	MUC17	nonsynonymous_SNV
LL004	chr7	101037930	G	C	exonic	MUC17	nonsynonymous_SNV
LL004	chr7	111746455	-	T	intronic	DOCK4	.
LL004	chr7	142469894	C	T	intergenic	TRY2P_MTRNR2L6	.
LL004	chr8	102551679	G	T	UTR5	ODF1	.
LL004	chr9	62844842	G	T	ncRNA_exonic	PTGER4P2-CDK2AP2P2	.
LL004	chr9	62858307	C	A	ncRNA_intronic	LOC403323	.
LL004	chr9	62858319	G	A	ncRNA_intronic	LOC403323	.
LL004	chr9	63819559	T	C	upstream_downstream	MIR4477B_LINC00537_MIR4477A	.
LL004	chr9	63819584	G	A	ncRNA_exonic	MIR4477A_MIR4477B	.
LL004	chr10	49470323	T	C	exonic	ERCC6	nonsynonymous_SNV
LL004	chr10	94358222	CA	-	intronic	NOC3L	.
LL004	chr11	5581385	A	G	exonic	OR52B6	nonsynonymous_SNV

LL004	chr11	5901270	T	C	intergenic	OR52E4_OR56A3	.
LL004	chr11	6716748	T	C	ncRNA_exonic	GVINP1	.
LL004	chr12	543134	TG	-	intronic	B4GALNT3	.
LL004	chr12	42109665	T	C	exonic	GXYLT1	synonymous_SNV
LL004	chr12	122715611	G	C	exonic	HCAR3	nonsynonymous_SNV
LL004	chr12	131799016	CT	-	intronic	SFSWAP	.
LL004	chr13	25096712	C	T	exonic	PABPC3	stopgain
LL004	chr14	90279078	T	C	exonic	NRDE2	nonsynonymous_SNV
LL004	chr15	33584316	A	G	intronic	RYR3	.
LL004	chr15	41573327	G	T	exonic	TYRO3	nonsynonymous_SNV
LL004	chr15	82238289	A	G	intronic	EFL1	.
LL004	chr16	2071832	T	C	exonic	TSC2	synonymous_SNV
LL004	chr16	34582987	G	C	intergenic	LINC00273_UBE2MP1	.
LL004	chr16	34583017	C	G	intergenic	LINC00273_UBE2MP1	.
LL004	chr16	34583045	G	A	intergenic	LINC00273_UBE2MP1	.
LL004	chr16	34583092	A	C	intergenic	LINC00273_UBE2MP1	.
LL004	chr16	34588255	G	C	intergenic	LINC00273_UBE2MP1	.
LL004	chr16	34588260	A	T	intergenic	LINC00273_UBE2MP1	.
LL004	chr16	46388671	T	A	intergenic	NONE_ANKRD26P1	.
LL004	chr16	46388753	T	A	intergenic	NONE_ANKRD26P1	.
LL004	chr16	46390081	C	G	intergenic	NONE_ANKRD26P1	.
LL004	chr16	46390581	G	C	intergenic	NONE_ANKRD26P1	.
LL004	chr16	46390633	T	C	intergenic	NONE_ANKRD26P1	.
LL004	chr16	46390643	T	A	intergenic	NONE_ANKRD26P1	.
LL004	chr16	46390661	G	A	intergenic	NONE_ANKRD26P1	.
LL004	chr16	46390706	T	C	intergenic	NONE_ANKRD26P1	.
LL004	chr16	46390759	G	A	intergenic	NONE_ANKRD26P1	.
LL004	chr16	46394682	T	C	intergenic	NONE_ANKRD26P1	.
LL004	chr16	46399206	C	T	intergenic	NONE_ANKRD26P1	.
LL004	chr16	46399271	G	C	intergenic	NONE_ANKRD26P1	.
LL004	chr16	46400868	A	T	intergenic	NONE_ANKRD26P1	.
LL004	chr16	46401045	C	A	intergenic	NONE_ANKRD26P1	.
LL004	chr16	46401096	C	T	intergenic	NONE_ANKRD26P1	.
LL004	chr16	46401425	T	C	intergenic	NONE_ANKRD26P1	.
LL004	chr17	7387502	T	C	intronic	TNK1	.
LL004	chr17	18699163	A	G	intergenic	ZNF286B_TRIM16L	.
LL004	chr17	19909228	T	C	exonic	AKAP10	nonsynonymous_SNV
LL004	chr17	21415939	C	T	exonic	KCNJ12	synonymous_SNV
LL004	chr17	31296270	C	T	exonic	OMG	nonsynonymous_SNV
LL004	chr17	40698154	G	A	UTR3	KRT24	.
LL004	chr17	74305293	A	G	exonic	DNAI2	synonymous_SNV
LL004	chr19	11617816	A	C	exonic	ZNF627	nonsynonymous_SNV

LL004	chr19	15658569	G	C	intronic	CYP4F3	.
LL004	chr19	35716434	G	C	intronic	ZBTB32	.
LL004	chr19	48680448	G	A	ncRNA_exonic	SEC1P	.
LL004	chr19	48918646	A	G	ncRNA_intronic	NUCB1-AS1	.
LL004	chr20	28565360	C	T	intergenic	NONE_FRG1CP	.
LL004	chr20	28565367	C	T	intergenic	NONE_FRG1CP	.
LL004	chr20	30399311	T	A	ncRNA_exonic	FRG1BP	.
LL004	chr20	30417599	A	C	ncRNA_exonic	FRG1BP	.
LL004	chr21	10397775	G	C	intergenic	LINC01667_BAGE	.
LL004	chr21	10397846	C	T	intergenic	LINC01667_BAGE	.
LL004	chr21	10473155	T	C	UTR3	BAGE2_BAGE3_BAGE4_BAGE5	.
LL004	chr21	10473159	G	A	UTR3	BAGE2_BAGE3_BAGE4_BAGE5	.
LL004	chr22	11279319	G	A	intergenic	LOC102723780_LOC102723769	.
LL004	chr22	11279338	G	A	intergenic	LOC102723780_LOC102723769	.
LL004	chr22	11279365	C	G	intergenic	LOC102723780_LOC102723769	.
LL004	chr22	20149712	C	T	intronic	CCDC188	.
LL004	chr22	20149762	G	A	UTR5	CCDC188	.
LL004	chr22	39103648	C	T	intronic	APOBEC3H	.
LL004	chr22	42509113	G	A	ncRNA_intronic	SERHL	.
LL004	chr22	42817625	-	A	intronic	ARFGAP3	.
LL004	chrM	13368	G	A	intergenic	RNR2_NONE	.
LL004	chr9_KI270720v1_random	5118	G	A	intergenic	NONE_NONE	.
LL004	chr9_KI270720v1_random	5153	T	C	intergenic	NONE_NONE	.
LL004	chr9_KI270720v1_random	5162	G	C	intergenic	NONE_NONE	.
LL004	chr17_GL000205v2_random	51379	G	A	intergenic	NONE_MGC70870	.
LL004	chr17_GL000205v2_random	51413	G	A	intergenic	NONE_MGC70870	.
LL004	chr17_GL000205v2_random	51415	A	G	intergenic	NONE_MGC70870	.
LL004	chr17_GL000205v2_random	51451	T	C	intergenic	NONE_MGC70870	.
LL004	chrUn_KI270438v1	104006	A	G	intergenic	NONE_NONE	.
LL004	chrUn_KI270438v1	104059	G	C	intergenic	NONE_NONE	.
LL004	chrUn_KI270438v1	104060	A	G	intergenic	NONE_NONE	.
LL004	chrUn_KI270438v1	104121	A	G	intergenic	NONE_NONE	.
LL004	chrUn_KI270438v1	104136	A	C	intergenic	NONE_NONE	.
LL004	chrUn_KI270438v1	104157	C	T	intergenic	NONE_NONE	.
LL004	chrUn_KI270438v1	104335	AATGGAATGGAATCG	-	intergenic	NONE_NONE	.
LL004	chrUn_KI270438v1	104677	T	C	intergenic	NONE_NONE	.
LL004	chrUn_KI270438v1	109252	G	C	intergenic	NONE_NONE	.
LL004	chrUn_KI270438v1	109272	T	G	intergenic	NONE_NONE	.
LL004	chrUn_KI270438v1	109273	G	T	intergenic	NONE_NONE	.
LL004	chrUn_KI270438v1	109376	T	C	intergenic	NONE_NONE	.
LL004	chrUn_KI270438v1	109509	A	G	intergenic	NONE_NONE	.
LL004	chrUn_KI270438v1	109521	T	G	intergenic	NONE_NONE	.

LL004	chrUn_KI270438v1	109602	C	T	intergenic	NONE_NONE	.
LL004	chrUn_KI270438v1	109659	AATGGAATGA	-	intergenic	NONE_NONE	.
LL004	chrUn_KI270438v1	109664	AATGA	-	intergenic	NONE_NONE	.
LL004	chrUn_KI270438v1	109766	A	T	intergenic	NONE_NONE	.
LL004	chrUn_KI270438v1	109812	C	G	intergenic	NONE_NONE	.
LL004	chrUn_KI270438v1	109844	A	T	intergenic	NONE_NONE	.
LL004	chrUn_KI270438v1	109873	G	A	intergenic	NONE_NONE	.
LL004	chrUn_KI270438v1	109945	C	T	intergenic	NONE_NONE	.
LL004	chrUn_KI270438v1	110470	A	G	intergenic	NONE_NONE	.
LL004	chrUn_KI270438v1	110525	C	T	intergenic	NONE_NONE	.
LL004	chrUn_KI270438v1	112173	T	C	intergenic	NONE_NONE	.
LL004	chrUn_KI270438v1	112319	-	TAA	intergenic	NONE_NONE	.
LL004	chrUn_KI270438v1	112320	-	T	intergenic	NONE_NONE	.
LL004	chrUn_KI270438v1	112339	T	G	intergenic	NONE_NONE	.
LL004	chrUn_KI270438v1	112340	G	T	intergenic	NONE_NONE	.

Sample	Chr	Start	Ref	Alt	Func.refGene	Gene.refGene	ExonicFunc.refGene
LL005	chr1	26951532	C	T	exonic	KDF1	synonymous_SNV
LL005	chr1	125180080	G	C	intergenic	NONE_NONE	.
LL005	chr1	143214750	A	T	intergenic	NONE_LOC645166	.
LL005	chr1	146794258	T	A	ncRNA_exonic	HYDIN2	.
LL005	chr1	152215165	G	A	exonic	HRNR	nonsynonymous_SNV
LL005	chr1	155763594	T	C	intronic	GON4L	.
LL005	chr1	171557431	G	A	exonic	PRRC2C	synonymous_SNV
LL005	chr1	248650825	G	A	exonic	OR2T27	synonymous_SNV
LL005	chr2	178597767	A	G	exonic	TTN	synonymous_SNV
LL005	chr2	213147680	AA	-	intronic	IKZF2	.
LL005	chr3	75738643	A	G	exonic	ZNF717	nonsynonymous_SNV
LL005	chr3	75738661	-	T	exonic	ZNF717	frameshift_insertion
LL005	chr3	128813586	CA	-	UTR3	RAB7A	.
LL005	chr4	53416	C	T	UTR5	ZNF595	.
LL005	chr5	170080127	TG	-	intronic	DOCK2	.
LL005	chr7	65762926	T	C	ncRNA_intronic	CCT6P1_LOC441242	.
LL005	chr7	101037985	C	A	exonic	MUC17	nonsynonymous_SNV
LL005	chr7	142469894	C	T	intergenic	TRY2P_MTRNR2L6	.
LL005	chr8	21990344	G	C	exonic	XPO7	synonymous_SNV
LL005	chr9	34835276	A	C	ncRNA_exonic	FAM205BP	.
LL005	chr9	62844751	C	T	ncRNA_exonic	PTGER4P2-CDK2AP2P2	.
LL005	chr9	62844772	C	T	ncRNA_exonic	PTGER4P2-CDK2AP2P2	.
LL005	chr9	62844775	C	T	ncRNA_exonic	PTGER4P2-CDK2AP2P2	.
LL005	chr9	62844842	G	T	ncRNA_exonic	PTGER4P2-CDK2AP2P2	.

LL005	chr9	62858307	C	A	ncRNA_intronic	LOC403323	.
LL005	chr9	62858319	G	A	ncRNA_intronic	LOC403323	.
LL005	chr9	63819568	A	G	upstream_downstream	MIR4477B_LINC00537_MIR4477A	.
LL005	chr9	63819584	G	A	ncRNA_exonic	MIR4477A_MIR4477B	.
LL005	chr9	63819588	A	G	ncRNA_exonic	MIR4477A_MIR4477B	.
LL005	chr10	91942414	T	G	intronic	BTAF1	.
LL005	chr12	543134	TG	-	intronic	B4GALNT3	.
LL005	chr12	109092474	CA	-	intronic	ALKBH2	.
LL005	chr12	122715611	G	C	exonic	HCAR3	nonsynonymous_SNV
LL005	chr13	25096675	C	T	exonic	PABPC3	synonymous_SNV
LL005	chr13	25096712	C	T	exonic	PABPC3	stopgain
LL005	chr14	70457733	C	T	exonic	ADAM21	synonymous_SNV
LL005	chr14	78988048	T	G	exonic	NRXN3	nonsynonymous_SNV
LL005	chr15	41573327	G	T	exonic	TYRO3	nonsynonymous_SNV
LL005	chr15	64934110	A	T	intronic	ANKDD1A	.
LL005	chr16	2071832	T	C	exonic	TSC2	synonymous_SNV
LL005	chr16	34574039	C	T	intergenic	LINC00273_UBE2MP1	.
LL005	chr16	34582246	G	C	intergenic	LINC00273_UBE2MP1	.
LL005	chr16	34583045	G	A	intergenic	LINC00273_UBE2MP1	.
LL005	chr16	34583092	A	C	intergenic	LINC00273_UBE2MP1	.
LL005	chr16	34588070	G	A	intergenic	LINC00273_UBE2MP1	.
LL005	chr16	34588260	A	T	intergenic	LINC00273_UBE2MP1	.
LL005	chr16	46388667	ATCATCGAATGAGATCGA	-	intergenic	NONE_ANKRD26P1	.
LL005	chr16	46388671	T	A	intergenic	NONE_ANKRD26P1	.
LL005	chr16	46390581	G	C	intergenic	NONE_ANKRD26P1	.
LL005	chr16	46390633	T	C	intergenic	NONE_ANKRD26P1	.
LL005	chr16	46390670	C	T	intergenic	NONE_ANKRD26P1	.
LL005	chr16	46390802	A	T	intergenic	NONE_ANKRD26P1	.
LL005	chr16	46394682	T	C	intergenic	NONE_ANKRD26P1	.
LL005	chr16	46394756	T	A	intergenic	NONE_ANKRD26P1	.
LL005	chr16	46399271	G	C	intergenic	NONE_ANKRD26P1	.
LL005	chr16	46401096	C	T	intergenic	NONE_ANKRD26P1	.
LL005	chr16	87412447	G	A	exonic	ZCCHC14	synonymous_SNV
LL005	chr17	21415939	C	T	exonic	KCNJ12	synonymous_SNV
LL005	chr17	28911100	T	G	intronic	PHF12	.
LL005	chr17	47974545	G	A	UTR5	CDK5RAP3	.
LL005	chr18	58579210	T	C	exonic	ALPK2	synonymous_SNV
LL005	chr19	5111856	C	T	intronic	KDM4B	.

LL005	chr20	29496248	G	A	ncRNA_intronic	FRG1EP	.
LL005	chr20	29496310	T	C	ncRNA_intronic	FRG1EP	.
LL005	chr21	8420440	GT	-	intergenic	LOC100507412_MIR6724-4	.
LL005	chr21	10397775	G	C	intergenic	LINC01667_BAGE	.
LL005	chr21	10473159	G	A	UTR3	BAGE2_BAGE3_BAGE4_BAGE5	.
LL005	chr21	36397402	G	T	intronic	CHAF1B	.
LL005	chr22	11279319	G	A	intergenic	LOC102723780_LOC102723769	.
LL005	chr22	11279338	G	A	intergenic	LOC102723780_LOC102723769	.
LL005	chr22	11279365	C	G	intergenic	LOC102723780_LOC102723769	.
LL005	chr22	19089284	C	A	intronic	DGCR2	.
LL005	chr22	19132173	C	T	exonic	TSSK2	synonymous_SNV
LL005	chr22	20149762	G	A	UTR5	CCDC188	.
LL005	chr22	37725145	C	A	exonic	TRIOBP	nonsynonymous_SNV
LL005	chr22	42817625	-	A	intronic	ARFGAP3	.
LL005	chr22	50530398	AG	-	upstream_downstream	TYMP_ODF3B	.
LL005	chrM	13368	G	A	intergenic	RNR2_NONE	.
LL005	chr9_KI270720v1_random	5118	G	A	intergenic	NONE_NONE	.
LL005	chr9_KI270720v1_random	5153	T	C	intergenic	NONE_NONE	.
LL005	chr9_KI270720v1_random	5162	G	C	intergenic	NONE_NONE	.
LL005	chr17_GL000205v2_random	51379	G	A	intergenic	NONE_MGC70870	.
LL005	chr17_GL000205v2_random	51403	C	G	intergenic	NONE_MGC70870	.
LL005	chr17_GL000205v2_random	51413	G	A	intergenic	NONE_MGC70870	.
LL005	chr17_GL000205v2_random	51415	A	G	intergenic	NONE_MGC70870	.
LL005	chr17_GL000205v2_random	51437	G	A	intergenic	NONE_MGC70870	.
LL005	chr17_GL000205v2_random	51451	T	C	intergenic	NONE_MGC70870	.
LL005	chr17_GL000205v2_random	51460	G	A	intergenic	NONE_MGC70870	.
LL005	chr17_GL000205v2_random	51532	T	A	intergenic	NONE_MGC70870	.
LL005	chr22_KI270733v1_random	135755	C	T	downstream	RNA28SN1_RNA28SN2_RNA28SN4	.
LL005	chrUn_KI270438v1	104006	A	G	intergenic	NONE_NONE	.
LL005	chrUn_KI270438v1	104059	G	C	intergenic	NONE_NONE	.
LL005	chrUn_KI270438v1	104121	A	G	intergenic	NONE_NONE	.
LL005	chrUn_KI270438v1	104136	A	C	intergenic	NONE_NONE	.
LL005	chrUn_KI270438v1	104157	C	T	intergenic	NONE_NONE	.
LL005	chrUn_KI270438v1	109272	T	G	intergenic	NONE_NONE	.
LL005	chrUn_KI270438v1	109273	G	T	intergenic	NONE_NONE	.
LL005	chrUn_KI270438v1	109376	T	C	intergenic	NONE_NONE	.
LL005	chrUn_KI270438v1	109392	GGAACAGAATGGAAC	-	intergenic	NONE_NONE	.
LL005	chrUn_KI270438v1	109509	A	G	intergenic	NONE_NONE	.

LL005	chrUn_KI270438v1	109521	T	G	intergenic	NONE_NONE	.
LL005	chrUn_KI270438v1	109602	C	T	intergenic	NONE_NONE	.
LL005	chrUn_KI270438v1	109632	CGAATGGAATGGAAG	-	intergenic	NONE_NONE	.
LL005	chrUn_KI270438v1	109659	AATGGAATGA	-	intergenic	NONE_NONE	.
LL005	chrUn_KI270438v1	109664	AATGA	-	intergenic	NONE_NONE	.
LL005	chrUn_KI270438v1	109766	A	T	intergenic	NONE_NONE	.
LL005	chrUn_KI270438v1	109844	A	T	intergenic	NONE_NONE	.
LL005	chrUn_KI270438v1	109873	G	A	intergenic	NONE_NONE	.
LL005	chrUn_KI270438v1	109945	C	T	intergenic	NONE_NONE	.
LL005	chrUn_KI270438v1	109965	A	G	intergenic	NONE_NONE	.
LL005	chrUn_KI270438v1	109969	A	C	intergenic	NONE_NONE	.
LL005	chrUn_KI270438v1	110470	A	T	intergenic	NONE_NONE	.
LL005	chrUn_KI270438v1	112173	T	C	intergenic	NONE_NONE	.
LL005	chrUn_KI270438v1	112319	-	TAA	intergenic	NONE_NONE	.
LL005	chrUn_KI270438v1	112320	-	T	intergenic	NONE_NONE	.
LL005	chrUn_KI270438v1	112339	T	G	intergenic	NONE_NONE	.
LL005	chrUn_KI270438v1	112340	G	T	intergenic	NONE_NONE	.
LL005	chrUn_GL000220v1	142111	A	T	intergenic	LOC100507412_MIR6724-4	.
LL005	chrUn_GL000216v2	99158	G	T	intergenic	NONE_NONE	.
LL005	chrUn_GL000216v2	99177	C	A	intergenic	NONE_NONE	.
Sample	Chr	Start	Ref	Alt	Func.refGene	Gene.refGene	ExonicFunc.refGene
LL006	chr1	1647255	T	C	intronic	CDK11B	.
LL006	chr1	1657358	-	A	intronic	CDK11B	.
LL006	chr1	1668501	C	T	intronic	SLC35E2B	.
LL006	chr1	15397347	G	A	exonic	FHAD1	synonymous_SNV
LL006	chr1	46344998	T	C	exonic	NSUN4	synonymous_SNV
LL006	chr1	125180208	G	A	intergenic	NONE_NONE	.
LL006	chr1	125180250	A	T	intergenic	NONE_NONE	.
LL006	chr1	143214750	A	T	intergenic	NONE_LOC645166	.
LL006	chr1	147615921	T	A	intronic	BCL9	.
LL006	chr1	148952882	G	A	exonic	PDE4DIP	synonymous_SNV
LL006	chr1	148953097	C	T	exonic	PDE4DIP	nonsynonymous_SNV
LL006	chr1	152215165	G	A	exonic	HRNR	nonsynonymous_SNV
LL006	chr1	152355575	T	C	exonic	FLG2	synonymous_SNV
LL006	chr1	168576773	A	T	intronic	XCL1	.
LL006	chr1	168576778	A	T	intronic	XCL1	.
LL006	chr1	233353960	TG	-	intronic	MAP3K21	.
LL006	chr1	248650825	G	A	exonic	OR2T27	synonymous_SNV
LL006	chr2	37952272	TG	-	ncRNA_intronic	RMDN2-AS1	.
LL006	chr2	38798490	A	G	intronic	DHX57	.

LL006	chr2	61840298	T	C	exonic	FAM161A	nonsynonymous_SNV
LL006	chr2	120131597	G	C	intronic	EPB41L5	.
LL006	chr2	132781993	G	A	exonic	NCKAP5	synonymous_SNV
LL006	chr2	213147680	AA	-	intronic	IKZF2	.
LL006	chr2	217848164	GCT	-	exonic	TNS1	nonframeshift_deletion
LL006	chr2	239578600	A	C	intergenic	LOC101928111_LOC150935	.
LL006	chr3	75738643	A	G	exonic	ZNF717	nonsynonymous_SNV
LL006	chr3	75738661	-	T	exonic	ZNF717	frameshift_insertion
LL006	chr3	108470146	G	A	exonic	MYH15	nonsynonymous_SNV
LL006	chr3	130471877	T	-	exonic	COL6A5	frameshift_deletion
LL006	chr3	142558694	A	G	exonic	ATR	synonymous_SNV
LL006	chr4	40438198	T	C	exonic	RBM47	synonymous_SNV
LL006	chr5	35753613	T	C	intronic	SPEF2	.
LL006	chr5	132674018	C	T	UTR5	IL4	.
LL006	chr6	8054329	T	C	ncRNA_intronic	BLOC1S5-TXNDC5_EEF1E1-BLOC	.
LL006	chr6	26027205	G	A	exonic	HIST1H4B	synonymous_SNV
LL006	chr6	151365770	C	T	exonic	ZBTB2	synonymous_SNV
LL006	chr6	167375916	C	G	intronic	TCP10	.
LL006	chr7	64991278	G	A	exonic	ERV3-1	synonymous_SNV
LL006	chr7	65762926	T	C	ncRNA_intronic	CCT6P1_LOC441242	.
LL006	chr7	101037930	G	C	exonic	MUC17	nonsynonymous_SNV
LL006	chr7	130215997	C	T	exonic	SSMEM1	nonsynonymous_SNV
LL006	chr7	134941283	G	A	intronic	CALD1	.
LL006	chr7	152247975	G	A	exonic	KMT2C	nonsynonymous_SNV
LL006	chr7	157526018	C	T	intergenic	LOC101927914_PTPRN2	.
LL006	chr8	10610141	-	CTC	exonic	RP1L1	frameshift_insertion
LL006	chr8	11838363	T	C	intronic	FDFT1	.
LL006	chr9	34835276	A	C	ncRNA_exonic	FAM205BP	.
LL006	chr9	62844751	C	T	ncRNA_exonic	PTGER4P2-CDK2AP2P2	.
LL006	chr9	62844772	C	T	ncRNA_exonic	PTGER4P2-CDK2AP2P2	.
LL006	chr9	62844842	G	T	ncRNA_exonic	PTGER4P2-CDK2AP2P2	.
LL006	chr9	62858307	C	A	ncRNA_intronic	LOC403323	.
LL006	chr9	63819568	A	G	upstream_downstream	MIR4477B_LINC00537_MIR4477A	.
LL006	chr9	63819584	G	A	ncRNA_exonic	MIR4477A_MIR4477B	.
LL006	chr9	65646931	A	T	intergenic	FOXD4L5_CBWD5	.
LL006	chr9	122144177	C	T	UTR3	NDUFA8	.
LL006	chr10	49470323	T	C	exonic	ERCC6	nonsynonymous_SNV
LL006	chr10	91942414	T	G	intronic	BTAF1	.
LL006	chr10	94358222	CA	-	intronic	NOC3L	.
LL006	chr11	78672291	G	A	exonic	TENM4	synonymous_SNV
LL006	chr12	543134	TG	-	intronic	B4GALNT3	.
LL006	chr12	4912062	T	C	exonic	KCNA1	synonymous_SNV

LL006	chr12	21638457	A	C	exonic	LDHB	nonsynonymous_SNV
LL006	chr12	40542710	C	A	intronic	MUC19	.
LL006	chr12	55412350	T	C	intergenic	OR6C65_OR6C76	.
LL006	chr12	122715611	G	C	exonic	HCAR3	nonsynonymous_SNV
LL006	chr12	131799016	CT	-	intronic	SFSWAP	.
LL006	chr13	25096712	C	T	exonic	PABPC3	stopgain
LL006	chr14	70457733	C	T	exonic	ADAM21	synonymous_SNV
LL006	chr14	78988048	T	G	exonic	NRXN3	nonsynonymous_SNV
LL006	chr14	88185618	C	T	exonic	KCNK10	nonsynonymous_SNV
LL006	chr15	33584316	A	G	intronic	RYR3	.
LL006	chr15	41573327	G	T	exonic	TYRO3	nonsynonymous_SNV
LL006	chr16	21179532	A	C	exonic	TMEM159	nonsynonymous_SNV
LL006	chr16	34583017	C	G	intergenic	LINC00273_UBE2MP1	.
LL006	chr16	34583045	G	A	intergenic	LINC00273_UBE2MP1	.
LL006	chr16	34583092	A	C	intergenic	LINC00273_UBE2MP1	.
LL006	chr16	34588150	G	A	intergenic	LINC00273_UBE2MP1	.
LL006	chr16	34588260	A	T	intergenic	LINC00273_UBE2MP1	.
LL006	chr16	46388667	ATCATCGAATGAGATCG/	-	intergenic	NONE_ANKRD26P1	.
LL006	chr16	46388671	T	A	intergenic	NONE_ANKRD26P1	.
LL006	chr16	46390250	T	C	intergenic	NONE_ANKRD26P1	.
LL006	chr16	46390369	T	A	intergenic	NONE_ANKRD26P1	.
LL006	chr16	46390633	T	C	intergenic	NONE_ANKRD26P1	.
LL006	chr16	46390643	T	A	intergenic	NONE_ANKRD26P1	.
LL006	chr16	46390670	C	T	intergenic	NONE_ANKRD26P1	.
LL006	chr16	46390759	G	A	intergenic	NONE_ANKRD26P1	.
LL006	chr16	46390802	A	T	intergenic	NONE_ANKRD26P1	.
LL006	chr16	46394526	G	A	intergenic	NONE_ANKRD26P1	.
LL006	chr16	46394682	T	C	intergenic	NONE_ANKRD26P1	.
LL006	chr16	46399525	G	T	intergenic	NONE_ANKRD26P1	.
LL006	chr16	46400864	C	T	intergenic	NONE_ANKRD26P1	.
LL006	chr16	46401096	C	T	intergenic	NONE_ANKRD26P1	.
LL006	chr16	46401425	T	C	intergenic	NONE_ANKRD26P1	.
LL006	chr16	74661181	G	T	exonic	RFWD3	nonsynonymous_SNV
LL006	chr17	18889912	A	G	intronic	PRPSAP2	.
LL006	chr17	19909228	T	C	exonic	AKAP10	nonsynonymous_SNV
LL006	chr17	28911100	T	G	intronic	PHF12	.
LL006	chr17	31296270	C	T	exonic	OMG	nonsynonymous_SNV
LL006	chr17	40698154	G	A	UTR3	KRT24	.
LL006	chr17	67743673	A	G	intronic	NOL11	.
LL006	chr18	13826392	C	G	exonic	MC5R	nonsynonymous_SNV
LL006	chr18	58579210	T	C	exonic	ALPK2	synonymous_SNV
LL006	chr19	4511528	TTGGCCACATTCGCAGC	-	exonic	PLIN4	nonframeshift_deletion

LL006	chr19	15658639	A	G	intronic	CYP4F3	.
LL006	chr19	48206378	G	A	intergenic	C19orf68_CARD8	.
LL006	chr20	28565378	G	A	intergenic	NONE_FRG1CP	.
LL006	chr20	29496263	A	G	ncRNA_intronic	FRG1EP	.
LL006	chr20	29496310	T	C	ncRNA_intronic	FRG1EP	.
LL006	chr21	10397775	G	C	intergenic	LINC01667_BAGE	.
LL006	chr21	10397846	C	T	intergenic	LINC01667_BAGE	.
LL006	chr21	10397887	-	T	intergenic	LINC01667_BAGE	.
LL006	chr21	10473159	G	A	UTR3	BAGE2_BAGE3_BAGE4_BAGE5	.
LL006	chr22	11279319	G	A	intergenic	LOC102723780_LOC102723769	.
LL006	chr22	11279338	G	A	intergenic	LOC102723780_LOC102723769	.
LL006	chr22	11279365	C	G	intergenic	LOC102723780_LOC102723769	.
LL006	chr22	20149712	C	T	intronic	CCDC188	.
LL006	chr22	20149762	G	A	UTR5	CCDC188	.
LL006	chr22	20976319	C	T	intronic	AIFM3	.
LL006	chr22	42509113	G	A	ncRNA_intronic	SERHL	.
LL006	chr22	42817625	-	A	intronic	ARFGAP3	.
LL006	chr22	50530398	AG	-	upstream_downstream	TYMP_ODF3B	.
LL006	chrX	74591860	A	G	exonic	RLIM	synonymous_SNV
LL006	chrX	74591902	T	A	exonic	RLIM	synonymous_SNV
LL006	chrX	74591904	A	G	exonic	RLIM	nonsynonymous_SNV
LL006	chrX	74591920	C	G	exonic	RLIM	synonymous_SNV
LL006	chrX	74591926	G	T	exonic	RLIM	synonymous_SNV
LL006	chrX	89922674	G	A	exonic	TGIF2LX	nonsynonymous_SNV
LL006	chrM	4917	A	G	intergenic	RNR2_NONE	.
LL006	chrM	7158	A	G	intergenic	RNR2_NONE	.
LL006	chr9_KI270720v1_random	5118	G	A	intergenic	NONE_NONE	.
LL006	chr9_KI270720v1_random	5153	T	C	intergenic	NONE_NONE	.
LL006	chr9_KI270720v1_random	5162	G	C	intergenic	NONE_NONE	.
LL006	chr17_GL000205v2_random	51415	A	G	intergenic	NONE_MGC70870	.
LL006	chrUn_KI270438v1	104006	A	G	intergenic	NONE_NONE	.
LL006	chrUn_KI270438v1	104036	A	G	intergenic	NONE_NONE	.
LL006	chrUn_KI270438v1	104059	G	C	intergenic	NONE_NONE	.
LL006	chrUn_KI270438v1	104121	A	G	intergenic	NONE_NONE	.
LL006	chrUn_KI270438v1	104136	A	C	intergenic	NONE_NONE	.
LL006	chrUn_KI270438v1	104876	ATGGAATGG	-	intergenic	NONE_NONE	.
LL006	chrUn_KI270438v1	109272	T	G	intergenic	NONE_NONE	.
LL006	chrUn_KI270438v1	109273	G	T	intergenic	NONE_NONE	.
LL006	chrUn_KI270438v1	109376	T	C	intergenic	NONE_NONE	.
LL006	chrUn_KI270438v1	109402	G	C	intergenic	NONE_NONE	.
LL006	chrUn_KI270438v1	109423	G	C	intergenic	NONE_NONE	.
LL006	chrUn_KI270438v1	109424	-	AT	intergenic	NONE_NONE	.

LL006	chrUn_KI270438v1	109435	-	TGG	intergenic	NONE_NONE	.
LL006	chrUn_KI270438v1	109509	A	G	intergenic	NONE_NONE	.
LL006	chrUn_KI270438v1	109521	T	G	intergenic	NONE_NONE	.
LL006	chrUn_KI270438v1	109602	C	T	intergenic	NONE_NONE	.
LL006	chrUn_KI270438v1	109659	AATGGAATGA	-	intergenic	NONE_NONE	.
LL006	chrUn_KI270438v1	109664	AATGA	-	intergenic	NONE_NONE	.
LL006	chrUn_KI270438v1	109766	A	T	intergenic	NONE_NONE	.
LL006	chrUn_KI270438v1	109791	T	A	intergenic	NONE_NONE	.
LL006	chrUn_KI270438v1	109812	C	G	intergenic	NONE_NONE	.
LL006	chrUn_KI270438v1	109844	A	T	intergenic	NONE_NONE	.
LL006	chrUn_KI270438v1	109873	G	A	intergenic	NONE_NONE	.
LL006	chrUn_KI270438v1	112173	T	C	intergenic	NONE_NONE	.
LL006	chrUn_KI270438v1	112319	G	C	intergenic	NONE_NONE	.
Sample	Chr	Start	Ref	Alt	Func.refGene	Gene.refGene	ExonicFunc.refGene
LL007	chr1	1647255	T	C	intronic	CDK11B	.
LL007	chr1	1657358	-	A	intronic	CDK11B	.
LL007	chr1	11668809	A	G	exonic	FBXO6	nonsynonymous_SNV
LL007	chr1	15716230	C	-	intronic	PLEKHM2	.
LL007	chr1	125180080	G	C	intergenic	NONE_NONE	.
LL007	chr1	143214750	A	T	intergenic	NONE_LOC645166	.
LL007	chr1	148952882	G	A	exonic	PDE4DIP	synonymous_SNV
LL007	chr1	148953097	C	T	exonic	PDE4DIP	nonsynonymous_SNV
LL007	chr1	152215165	G	A	exonic	HRNR	nonsynonymous_SNV
LL007	chr1	233353960	TG	-	intronic	MAP3K21	.
LL007	chr2	120131597	G	C	intronic	EPB41L5	.
LL007	chr2	213147680	AA	-	intronic	IKZF2	.
LL007	chr3	75738643	A	G	exonic	ZNF717	nonsynonymous_SNV
LL007	chr3	108470146	G	A	exonic	MYH15	nonsynonymous_SNV
LL007	chr3	142558694	A	G	exonic	ATR	synonymous_SNV
LL007	chr4	53416	C	T	UTR5	ZNF595	.
LL007	chr4	25314033	A	T	intronic	ZCCHC4	.
LL007	chr5	77311802	-	T	intronic	PDE8B	.
LL007	chr5	128186488	G	T	intronic	SLC12A2	.
LL007	chr7	65762926	T	C	ncRNA_intronic	CCT6P1_LOC441242	.
LL007	chr7	101037930	G	C	exonic	MUC17	nonsynonymous_SNV
LL007	chr7	142469894	C	T	intergenic	TRY2P_MTRNR2L6	.
LL007	chr9	6986464	G	C	exonic	KDM4C	nonsynonymous_SNV
LL007	chr9	62844751	C	T	ncRNA_exonic	PTGER4P2-CDK2AP2P2	.
LL007	chr9	62844772	C	T	ncRNA_exonic	PTGER4P2-CDK2AP2P2	.
LL007	chr9	62858319	G	A	ncRNA_intronic	LOC403323	.
LL007	chr9	63819568	A	G	upstream_downstream	MIR4477B_LINC00537_MIR4477A	.
LL007	chr9	114096345	T	G	intronic	KIF12	.

LL007	chr9	122750129	C	T	exonic	OR1L6	synonymous_SNV
LL007	chr10	91942414	T	G	intronic	BTAF1	.
LL007	chr10	94358222	CA	-	intronic	NOC3L	.
LL007	chr10	125896581	C	A	UTR5	FANK1	.
LL007	chr10	128106848	TG	-	exonic	MKI67	frameshift_deletion
LL007	chr11	8671858	G	A	intergenic	TRIM66_RPL27A	.
LL007	chr12	105052774	T	C	intronic	ALDH1L2	.
LL007	chr12	122715611	G	C	exonic	HCAR3	nonsynonymous_SNV
LL007	chr12	131799016	CT	-	intronic	SFSWAP	.
LL007	chr14	78988048	T	G	exonic	NRXN3	nonsynonymous_SNV
LL007	chr15	50484443	A	G	intronic	USP8	.
LL007	chr16	34583017	C	G	intergenic	LINC00273_UBE2MP1	.
LL007	chr16	34583045	G	A	intergenic	LINC00273_UBE2MP1	.
LL007	chr16	34583092	A	C	intergenic	LINC00273_UBE2MP1	.
LL007	chr16	34588255	G	C	intergenic	LINC00273_UBE2MP1	.
LL007	chr16	34588260	A	T	intergenic	LINC00273_UBE2MP1	.
LL007	chr16	46388667	ATCATCGAATGAGATCGA	-	intergenic	NONE_ANKRD26P1	.
LL007	chr16	46388671	T	A	intergenic	NONE_ANKRD26P1	.
LL007	chr16	46390081	C	G	intergenic	NONE_ANKRD26P1	.
LL007	chr16	46390643	T	A	intergenic	NONE_ANKRD26P1	.
LL007	chr16	46394682	T	C	intergenic	NONE_ANKRD26P1	.
LL007	chr16	46394751	T	A	intergenic	NONE_ANKRD26P1	.
LL007	chr16	46398828	G	A	intergenic	NONE_ANKRD26P1	.
LL007	chr16	46400864	C	T	intergenic	NONE_ANKRD26P1	.
LL007	chr16	46400868	A	T	intergenic	NONE_ANKRD26P1	.
LL007	chr16	46401096	C	T	intergenic	NONE_ANKRD26P1	.
LL007	chr16	46401425	T	C	intergenic	NONE_ANKRD26P1	.
LL007	chr18	13826392	C	G	exonic	MC5R	nonsynonymous_SNV
LL007	chr20	29496310	T	C	ncRNA_intronic	FRG1EP	.
LL007	chr21	10397775	G	C	intergenic	LINC01667_BAGE	.
LL007	chr21	10397846	C	T	intergenic	LINC01667_BAGE	.
LL007	chr21	10473159	G	A	UTR3	BAGE2_BAGE3_BAGE4_BAGE5	.
LL007	chr22	11066568	T	G	intergenic	LOC102723780_LOC102723769	.
LL007	chr22	11279319	G	A	intergenic	LOC102723780_LOC102723769	.
LL007	chr22	11279338	G	A	intergenic	LOC102723780_LOC102723769	.
LL007	chr22	20149762	G	A	UTR5	CCDC188	.
LL007	chr22	42032723	A	T	intergenic	WBP2NL_NAGA	.
LL007	chr22	42509113	G	A	ncRNA_intronic	SERHL	.
LL007	chrM	15607	A	G	intergenic	RNR2_NONE	.
LL007	chr9_KI270720v1_random	5118	G	A	intergenic	NONE_NONE	.
LL007	chr17_GL000205v2_random	51379	G	A	intergenic	NONE_MGC70870	.
LL007	chr17_GL000205v2_random	51413	G	A	intergenic	NONE_MGC70870	.

LL007	chr17_GL000205v2_random	51415	A	G	intergenic	NONE_MGC70870	.
LL007	chr17_GL000205v2_random	51437	G	A	intergenic	NONE_MGC70870	.
LL007	chr17_GL000205v2_random	51451	T	C	intergenic	NONE_MGC70870	.
LL007	chrUn_KI270438v1	104006	A	G	intergenic	NONE_NONE	.
LL007	chrUn_KI270438v1	104059	G	C	intergenic	NONE_NONE	.
LL007	chrUn_KI270438v1	104121	A	G	intergenic	NONE_NONE	.
LL007	chrUn_KI270438v1	104136	A	C	intergenic	NONE_NONE	.
LL007	chrUn_KI270438v1	104311	A	G	intergenic	NONE_NONE	.
LL007	chrUn_KI270438v1	104677	T	C	intergenic	NONE_NONE	.
LL007	chrUn_KI270438v1	104695	A	G	intergenic	NONE_NONE	.
LL007	chrUn_KI270438v1	104707	T	G	intergenic	NONE_NONE	.
LL007	chrUn_KI270438v1	109252	G	C	intergenic	NONE_NONE	.
LL007	chrUn_KI270438v1	109272	T	G	intergenic	NONE_NONE	.
LL007	chrUn_KI270438v1	109376	T	C	intergenic	NONE_NONE	.
LL007	chrUn_KI270438v1	109423	G	C	intergenic	NONE_NONE	.
LL007	chrUn_KI270438v1	109424	-	AT	intergenic	NONE_NONE	.
LL007	chrUn_KI270438v1	109435	-	TGG	intergenic	NONE_NONE	.
LL007	chrUn_KI270438v1	109602	C	T	intergenic	NONE_NONE	.
LL007	chrUn_KI270438v1	109659	AATGGAATGA	-	intergenic	NONE_NONE	.
LL007	chrUn_KI270438v1	109664	AATGA	-	intergenic	NONE_NONE	.
LL007	chrUn_KI270438v1	109873	G	A	intergenic	NONE_NONE	.
LL007	chrUn_KI270438v1	109945	C	T	intergenic	NONE_NONE	.
LL007	chrUn_KI270438v1	112173	T	C	intergenic	NONE_NONE	.
LL007	chrUn_KI270438v1	112319	G	C	intergenic	NONE_NONE	.
LL007	chrUn_KI270438v1	112339	T	G	intergenic	NONE_NONE	.
LL007	chrUn_KI270438v1	112340	G	T	intergenic	NONE_NONE	.
Sample	Chr	Start	Ref	Alt	Func.refGene	Gene.refGene	ExonicFunc.refGene
LL008	chr1	1657358	-	A	intronic	CDK11B	.
LL008	chr1	21977031	G	A	UTR5	CELA3B	.
LL008	chr1	26951532	C	T	exonic	KDF1	synonymous_SNV
LL008	chr1	63551707	T	-	intronic	EFCAB7	.
LL008	chr1	125180336	G	C	intergenic	NONE_NONE	.
LL008	chr1	125180365	A	C	intergenic	NONE_NONE	.
LL008	chr1	152215165	G	A	exonic	HRNR	nonsynonymous_SNV
LL008	chr1	172442356	A	G	exonic	PIGC	synonymous_SNV
LL008	chr1	178520597	G	A	intronic	TEX35	.
LL008	chr1	207062609	G	A	intronic	PFKFB2	.
LL008	chr1	248650825	G	A	exonic	OR2T27	synonymous_SNV
LL008	chr2	120131597	G	C	intronic	EPB41L5	.
LL008	chr2	239578600	A	C	intergenic	LOC101928111_LOC150935	.
LL008	chr4	53416	C	T	UTR5	ZNF595	.
LL008	chr4	147857534	-	T	intronic	ARHGAP10	.

LL008	chr5	109774836	T	A	exonic	MAN2A1	synonymous_SNV
LL008	chr5	132674018	C	T	UTR5	IL4	.
LL008	chr6	8054329	T	C	ncRNA_intronic	BLOC1S5-TXNDC5_EEF1E1-BLOC	.
LL008	chr6	137203699	G	A	intronic	IFNGR1	.
LL008	chr7	65762926	T	C	ncRNA_intronic	CCT6P1_LOC441242	.
LL008	chr7	98903333	C	T	intronic	TRRAP	.
LL008	chr7	100959582	T	C	exonic	MUC3A	synonymous_SNV
LL008	chr7	101034678	G	T	exonic	MUC17	nonsynonymous_SNV
LL008	chr7	101037930	G	C	exonic	MUC17	nonsynonymous_SNV
LL008	chr7	128502534	G	-	UTR3	METTL2B	.
LL008	chr8	141166181	G	A	intronic	DENND3	.
LL008	chr9	62844751	C	T	ncRNA_exonic	PTGER4P2-CDK2AP2P2	.
LL008	chr9	63819584	G	A	ncRNA_exonic	MIR4477A_MIR4477B	.
LL008	chr9	133108117	T	C	exonic	RALGDS	synonymous_SNV
LL008	chr10	68471944	A	G	UTR5	DNA2	.
LL008	chr10	94358222	CA	-	intronic	NOC3L	.
LL008	chr11	119134422	C	T	exonic	HINFP	nonsynonymous_SNV
LL008	chr12	40542710	C	A	intronic	MUC19	.
LL008	chr13	25096712	C	T	exonic	PABPC3	stopgain
LL008	chr15	33584316	A	G	intronic	RYR3	.
LL008	chr15	33584342	C	T	intronic	RYR3	.
LL008	chr15	41573327	G	T	exonic	TYRO3	nonsynonymous_SNV
LL008	chr16	2449029	A	G	intronic	CCNF	.
LL008	chr16	34582246	G	C	intergenic	LINC00273_UBE2MP1	.
LL008	chr16	34582987	G	C	intergenic	LINC00273_UBE2MP1	.
LL008	chr16	34583017	C	G	intergenic	LINC00273_UBE2MP1	.
LL008	chr16	34583045	G	A	intergenic	LINC00273_UBE2MP1	.
LL008	chr16	34583092	A	C	intergenic	LINC00273_UBE2MP1	.
LL008	chr16	34588260	A	T	intergenic	LINC00273_UBE2MP1	.
LL008	chr16	46387909	A	G	intergenic	NONE_ANKRD26P1	.
LL008	chr16	46388667	ATCATCGAATGAGATCGA	-	intergenic	NONE_ANKRD26P1	.
LL008	chr16	46388671	T	A	intergenic	NONE_ANKRD26P1	.
LL008	chr16	46390081	C	G	intergenic	NONE_ANKRD26P1	.
LL008	chr16	46390633	T	C	intergenic	NONE_ANKRD26P1	.
LL008	chr16	46390661	G	A	intergenic	NONE_ANKRD26P1	.
LL008	chr16	46390670	C	T	intergenic	NONE_ANKRD26P1	.
LL008	chr16	46390706	T	C	intergenic	NONE_ANKRD26P1	.
LL008	chr16	46390802	A	T	intergenic	NONE_ANKRD26P1	.
LL008	chr16	46394526	G	A	intergenic	NONE_ANKRD26P1	.
LL008	chr16	46394682	T	C	intergenic	NONE_ANKRD26P1	.
LL008	chr16	46394756	T	A	intergenic	NONE_ANKRD26P1	.
LL008	chr16	46400864	C	T	intergenic	NONE_ANKRD26P1	.

LL008	chr16	46400868	A	T	intergenic	NONE_ANKRD26P1	.
LL008	chr16	46401096	C	T	intergenic	NONE_ANKRD26P1	.
LL008	chr16	87412447	G	A	exonic	ZCCHC14	synonymous_SNV
LL008	chr17	18699163	A	G	intergenic	ZNF286B_TRIM16L	.
LL008	chr17	18775235	C	T	intronic	FBXW10	.
LL008	chr17	28911100	T	G	intronic	PHF12	.
LL008	chr17	40698154	G	A	UTR3	KRT24	.
LL008	chr17	43814069	G	A	exonic	MPP3	synonymous_SNV
LL008	chr17	44398615	G	C	exonic	GPATCH8	synonymous_SNV
LL008	chr18	58579210	T	C	exonic	ALPK2	synonymous_SNV
LL008	chr19	4024022	G	A	intronic	PIAS4	.
LL008	chr19	6988780	G	C	ncRNA_intronic	ADGRE4P	.
LL008	chr19	10638144	T	C	intronic	SLC44A2	.
LL008	chr20	29496310	T	C	ncRNA_intronic	FRG1EP	.
LL008	chr20	63359522	T	C	intronic	CHRNA4	.
LL008	chr21	10397775	G	C	intergenic	LINC01667_BAGE	.
LL008	chr21	10473155	T	C	UTR3	BAGE2_BAGE3_BAGE4_BAGE5	.
LL008	chr21	10473159	G	A	UTR3	BAGE2_BAGE3_BAGE4_BAGE5	.
LL008	chr22	11279319	G	A	intergenic	LOC102723780_LOC102723769	.
LL008	chr22	11279338	G	A	intergenic	LOC102723780_LOC102723769	.
LL008	chr22	11279365	C	G	intergenic	LOC102723780_LOC102723769	.
LL008	chr22	20149762	G	A	UTR5	CCDC188	.
LL008	chr22	42509113	G	A	ncRNA_intronic	SERHL	.
LL008	chr22	42817625	-	A	intronic	ARFGAP3	.
LL008	chrM	7158	A	G	intergenic	RNR2_NONE	.
LL008	chrM	13368	G	A	intergenic	RNR2_NONE	.
LL008	chrM	15452	C	A	intergenic	RNR2_NONE	.
LL008	chrM	15607	A	G	intergenic	RNR2_NONE	.
LL008	chr9_KI270720v1_random	5118	G	A	intergenic	NONE_NONE	.
LL008	chr17_GL000205v2_random	51415	A	G	intergenic	NONE_MGC70870	.
LL008	chr17_GL000205v2_random	51451	T	C	intergenic	NONE_MGC70870	.
LL008	chrUn_KI270438v1	104006	A	G	intergenic	NONE_NONE	.
LL008	chrUn_KI270438v1	104059	G	C	intergenic	NONE_NONE	.
LL008	chrUn_KI270438v1	104093	G	A	intergenic	NONE_NONE	.
LL008	chrUn_KI270438v1	104311	A	G	intergenic	NONE_NONE	.
LL008	chrUn_KI270438v1	104695	A	G	intergenic	NONE_NONE	.
LL008	chrUn_KI270438v1	109252	G	C	intergenic	NONE_NONE	.
LL008	chrUn_KI270438v1	109423	G	C	intergenic	NONE_NONE	.
LL008	chrUn_KI270438v1	109424	-	AT	intergenic	NONE_NONE	.
LL008	chrUn_KI270438v1	109435	-	TGC	intergenic	NONE_NONE	.
LL008	chrUn_KI270438v1	109521	T	G	intergenic	NONE_NONE	.
LL008	chrUn_KI270438v1	109600	C	A	intergenic	NONE_NONE	.

LL008	chrUn_KI270438v1	109632	CGAATGGAATGGAAG	-	intergenic	NONE_NONE	.
LL008	chrUn_KI270438v1	109664	AATGA	-	intergenic	NONE_NONE	.
LL008	chrUn_KI270438v1	109766	A	T	intergenic	NONE_NONE	.
LL008	chrUn_KI270438v1	109813	G	A	intergenic	NONE_NONE	.
LL008	chrUn_KI270438v1	109868	G	T	intergenic	NONE_NONE	.
LL008	chrUn_KI270438v1	109873	G	A	intergenic	NONE_NONE	.
LL008	chrUn_KI270438v1	109945	C	T	intergenic	NONE_NONE	.
LL008	chrUn_KI270438v1	110525	C	T	intergenic	NONE_NONE	.
LL008	chrUn_KI270438v1	112173	T	C	intergenic	NONE_NONE	.
LL008	chrUn_KI270438v1	112319	G	C	intergenic	NONE_NONE	.
LL008	chrUn_KI270744v1	77837	G	A	intergenic	NONE_NONE	.
LL008	chrUn_GL000216v2	99158	G	T	intergenic	NONE_NONE	.
LL008	chrUn_GL000216v2	99177	C	A	intergenic	NONE_NONE	.
Sample	Chr	Start	Ref	Alt	Func.refGene	Gene.refGene	ExonicFunc.refGene
s2	chr1	17556	C	T	ncRNA_intronic	WASH7P	.
s2	chr1	183937	G	A	intergenic	LOC729737_MIR6859-4	.
s2	chr1	12881867	C	T	exonic	PRAMEF4	nonsynonymous_SNV
s2	chr1	12881880	G	A	exonic	PRAMEF4	synonymous_SNV
s2	chr1	12893329	C	G	exonic	PRAMEF10	nonsynonymous_SNV
s2	chr1	12941422	T	C	exonic	PRAMEF6	nonsynonymous_SNV
s2	chr1	13319482	A	C	exonic	PRAMEF15	nonsynonymous_SNV
s2	chr1	16536138	C	T	upstream	LINC01783	.
s2	chr1	16565461	C	T	intronic	NBPF1	.
s2	chr1	16565625	G	T	intronic	NBPF1	.
s2	chr1	16576328	G	C	exonic	NBPF1	unknown
s2	chr1	16580670	G	A	intronic	NBPF1	.
s2	chr1	16581396	T	C	intronic	NBPF1	.
s2	chr1	16581539	C	G	intronic	NBPF1	.
s2	chr1	16583468	G	A	intronic	NBPF1	.
s2	chr1	16627177	C	T	ncRNA_exonic	CROCCP2	.
s2	chr1	16645695	T	C	ncRNA_intronic	MST1P2	.
s2	chr1	16647559	A	G	ncRNA_exonic	MST1P2	.
s2	chr1	16647748	G	C	ncRNA_intronic	MST1P2	.
s2	chr1	16648263	G	A	ncRNA_exonic	MST1P2	.
s2	chr1	16648285	C	T	ncRNA_exonic	MST1P2	.
s2	chr1	16648291	G	C	ncRNA_exonic	MST1P2	.
s2	chr1	16649698	C	T	ncRNA_exonic	MST1P2	.
s2	chr1	16650155	G	A	ncRNA_exonic	MST1P2	.
s2	chr1	16650310	G	A	ncRNA_exonic	MST1P2	.
s2	chr1	16650312	C	T	ncRNA_exonic	MST1P2	.
s2	chr1	16757283	C	T	exonic	MST1L	synonymous_SNV
s2	chr1	16757423	C	T	intronic	MST1L	.

s2	chr1	16759300	G	T	exonic	MST1L	synonymous_SNV
s2	chr1	16760810	G	C	exonic	MST1L	synonymous_SNV
s2	chr1	22002726	C	T	intronic	CELA3A	.
s2	chr1	70862253	-	A	intronic	PTGER3	.
s2	chr1	120810541	C	T	exonic	NBPF26	nonsynonymous_SNV
s2	chr1	146989687	A	G	exonic	NBPF12	nonsynonymous_SNV
s2	chr1	148593699	C	G	exonic	NBPF14	nonsynonymous_SNV
s2	chr1	152214232	C	T	exonic	HRNR	nonsynonymous_SNV
s2	chr1	152215143	A	G	exonic	HRNR	synonymous_SNV
s2	chr1	152215642	C	T	exonic	HRNR	nonsynonymous_SNV
s2	chr1	152215843	C	T	exonic	HRNR	nonsynonymous_SNV
s2	chr1	152776551	C	T	exonic	LCE1F	synonymous_SNV
s2	chr1	154321988	A	G	exonic	AQP10	nonsynonymous_SNV
s2	chr1	248273336	A	G	exonic	OR2T33	nonsynonymous_SNV
s2	chr2	94199259	A	G	ncRNA_exonic	BMS1P14	.
s2	chr2	94199288	T	-	ncRNA_exonic	BMS1P14	.
s2	chr2	95935488	G	A	exonic	ANKRD36C	synonymous_SNV
s2	chr2	95945265	A	T	intronic	ANKRD36C	.
s2	chr2	113598529	T	C	ncRNA_intronic	WASH2P	.
s2	chr2	128318365	T	A	exonic	HS6ST1	stopgain
s2	chr2	232380577	G	T	intronic	ALPP	.
s2	chr3	75665744	G	A	exonic	FRG2C	synonymous_SNV
s2	chr3	75669319	A	G	intergenic	FRG2C_LINC00960	.
s2	chr3	125976844	G	A	intronic	ROPN1B	.
s2	chr3	195619990	T	C	intergenic	APOD_LOC105374297	.
s2	chr3	195975750	G	A	ncRNA_exonic	SDHAP1	.
s2	chr4	9243843	C	T	upstream	USP17L17	.
s2	chr4	9248531	C	A	upstream	USP17L11_USP17L18	.
s2	chr4	9248548	G	A	upstream	USP17L11_USP17L18	.
s2	chr4	9248616	T	A	upstream	USP17L11_USP17L18	.
s2	chr4	9268602	G	A	exonic	USP17L20_USP17L22	stopgain
s2	chr5	34191075	T	A	intergenic	C1QTNF3-AMACR_NONE	.
s2	chr5	34193423	A	G	intergenic	C1QTNF3-AMACR_NONE	.
s2	chr5	141123606	T	C	exonic	PCDHB4	synonymous_SNV
s2	chr5	141123609	T	C	exonic	PCDHB4	synonymous_SNV
s2	chr5	141201296	A	G	exonic	PCDHB11	nonsynonymous_SNV
s2	chr7	6757889	G	C	exonic	RSPH10B_RSPH10B2	nonsynonymous_SNV
s2	chr7	56373626	A	G	intergenic	NUPR2_LOC650226	.
s2	chr7	73225850	A	C	ncRNA_intronic	NCF1B	.
s2	chr7	75148237	T	C	exonic	GTF2IRD2B	nonsynonymous_SNV
s2	chr7	75148882	T	C	exonic	GTF2IRD2B	nonsynonymous_SNV
s2	chr7	100998641	C	G	exonic	MUC12	nonsynonymous_SNV

s2	chr7	100999874	A	T	exonic	MUC12	nonsynonymous_SNV
s2	chr7	101000011	G	A	exonic	MUC12	nonsynonymous_SNV
s2	chr7	101000063	G	T	exonic	MUC12	nonsynonymous_SNV
s2	chr7	101000457	G	A	exonic	MUC12	synonymous_SNV
s2	chr7	101000633	C	T	exonic	MUC12	nonsynonymous_SNV
s2	chr7	101000638	A	G	exonic	MUC12	nonsynonymous_SNV
s2	chr7	102639154	G	T	exonic	UPK3BL1	synonymous_SNV
s2	chr7	152265083	C	A	exonic	KMT2C	nonsynonymous_SNV
s2	chr8	85655689	G	T	upstream	REXO1L2P	.
s2	chr8	85655705	T	G	upstream	REXO1L2P	.
s2	chr8	85655709	C	A	upstream	REXO1L2P	.
s2	chr8	85655746	G	C	upstream	REXO1L2P	.
s2	chr9	34834503	G	A	ncRNA_exonic	FAM205BP	.
s2	chr9	62801388	C	G	upstream	LINC01410	.
s2	chr9	62858252	C	A	ncRNA_intronic	LOC403323	.
s2	chr9	65650207	G	A	intergenic	FOXD4L5_CBWD5	.
s2	chr9	65650228	G	A	intergenic	FOXD4L5_CBWD5	.
s2	chr9	65650238	C	T	intergenic	FOXD4L5_CBWD5	.
s2	chr9	96938627	C	T	exonic	NUTM2G	synonymous_SNV
s2	chr9	114323755	C	T	exonic	ORM1	synonymous_SNV
s2	chr9	122724407	T	C	exonic	OR1L4	nonsynonymous_SNV
s2	chr9	122724418	A	G	exonic	OR1L4	synonymous_SNV
s2	chr9	128153053	CA	-	intronic	LCN2	.
s2	chr9	134053401	A	C	exonic	BRD3	nonsynonymous_SNV
s2	chr9	134422453	A	C	intronic	RXRA	.
s2	chr9	136755870	G	A	intronic	LCN8	.
s2	chr10	73675610	C	T	exonic	AGAP5	synonymous_SNV
s2	chr10	79507580	C	G	ncRNA_exonic	LOC729815	.
s2	chr10	79559428	G	A	exonic	SFTPA2	nonsynonymous_SNV
s2	chr10	79611798	T	A	intronic	SFTPA1	.
s2	chr10	79611881	T	C	exonic	SFTPA1	nonsynonymous_SNV
s2	chr10	120886648	-	A	intronic	WDR11	.
s2	chr10	133625456	C	-	exonic	FRG2B	frameshift_deletion
s2	chr10	133625692	C	T	intronic	FRG2B	.
s2	chr10	133625702	C	T	intronic	FRG2B	.
s2	chr11	18247969	T	C	exonic	SAA2_SAA2-SAA4	nonsynonymous_SNV
s2	chr12	9425638	T	A	ncRNA_intronic	DDX12P	.
s2	chr12	31091769	C	T	exonic	DDX11	synonymous_SNV
s2	chr12	31091875	T	C	intronic	DDX11	.
s2	chr12	31100610	G	A	intronic	DDX11	.
s2	chr13	18177976	A	T	intergenic	NONE_FAM230C	.
s2	chr14	19402323	G	A	downstream	POTEG	.

s2	chr14	19414622	C	A	intronic	POTEG	.
s2	chr14	19678610	A	C	intergenic	POTEG_OR11H2	.
s2	chr14	104951388	C	G	exonic	AHNAK2	nonsynonymous_SNV
s2	chr15	20408783	-	T	ncRNA_intronic	HERC2P3	.
s2	chr15	101753899	A	G	intergenic	LOC100128108_OR4F6	.
s2	chr15	101757405	T	G	intergenic	LOC100128108_OR4F6	.
s2	chr15	101761203	T	C	intergenic	LOC100128108_OR4F6	.
s2	chr15	101772004	A	G	intergenic	LOC100128108_OR4F6	.
s2	chr16	12203286	C	G	intronic	SNX29	.
s2	chr16	22533964	G	C	exonic	NPIPB5	synonymous_SNV
s2	chr16	28723438	G	A	intronic	EIF3C_EIF3CL	.
s2	chr16	33741748	G	A	intergenic	LOC390705_ENPP7P13	.
s2	chr16	70131778	G	A	intronic	PDPR	.
s2	chr16	74391416	G	A	exonic	NPIPB15	nonsynonymous_SNV
s2	chr16	74409592	C	T	exonic	CLEC18B	nonsynonymous_SNV
s2	chr16	74409770	T	C	intronic	CLEC18B	.
s2	chr17	15738101	G	C	intronic	TBC1D26	.
s2	chr17	18441587	G	A	ncRNA_exonic	KRT16P1	.
s2	chr17	18492905	-	AG	intronic	LGALS9C	.
s2	chr17	20589700	C	A	intergenic	CDRT15L2_LINC02088	.
s2	chr17	21703201	G	A	exonic	KCNJ18	nonsynonymous_SNV
s2	chr18	47686	C	T	intergenic	LOC102723376_ROCK1P1	.
s2	chr18	48137	C	T	intergenic	LOC102723376_ROCK1P1	.
s2	chr18	74960489	C	G	intronic	ZNF407	.
s2	chr19	21569093	G	A	intergenic	ZNF429_LOC400682	.
s2	chr19	22665021	A	G	exonic	ZNF492	nonsynonymous_SNV
s2	chr19	39877979	G	A	exonic	FCGBP	synonymous_SNV
s2	chr19	39886648	T	C	intronic	FCGBP	.
s2	chr19	40850014	A	C	intronic	CYP2A6	.
s2	chr19	40850019	T	C	intronic	CYP2A6	.
s2	chr19	41088986	G	A	exonic	CYP2A13	nonsynonymous_SNV
s2	chr19	41089011	A	G	exonic	CYP2A13	nonsynonymous_SNV
s2	chr19	41089049	C	T	exonic	CYP2A13	stopgain
s2	chr19	41089070	G	A	exonic	CYP2A13	nonsynonymous_SNV
s2	chr19	49044175	A	G	UTR5	CGB5	.
s2	chr19	49044189	G	C	UTR5	CGB5	.
s2	chr19	49969964	T	C	exonic	SIGLEC16	nonsynonymous_SNV
s2	chr19	55772851	A	T	exonic	RFPL4AL1	nonsynonymous_SNV
s2	chr20	29496341	T	G	ncRNA_intronic	FRG1EP	.
s2	chr20	30417462	C	G	ncRNA_exonic	FRG1BP	.
s2	chr20	30815144	C	A	intergenic	FRG1BP_DEFB115	.
s2	chr20	30815156	G	T	intergenic	FRG1BP_DEFB115	.

s2	chr20	30815433	C	T	intergenic	FRG1BP_DEFB115	.
s2	chr21	8420440	GT	-	intergenic	LOC100507412_MIR6724-4	.
s2	chr21	9068586	A	G	ncRNA_exonic	TEKT4P2	.
s2	chr21	9068589	G	A	ncRNA_exonic	TEKT4P2	.
s2	chr21	10462834	A	G	splicing	BAGE2_BAGE3_BAGE4_BAGE5	.
s2	chr21	10462836	C	T	exonic	BAGE2_BAGE3	stopgain
s2	chr21	10462861	G	A	exonic	BAGE2_BAGE3	nonsynonymous_SNV
s2	chr21	10462962	G	T	UTR3	BAGE2_BAGE3_BAGE4_BAGE5	.
s2	chr21	10463002	C	T	UTR3	BAGE2_BAGE3_BAGE4_BAGE5	.
s2	chr21	10473245	G	A	UTR3	BAGE2_BAGE3_BAGE4_BAGE5	.
s2	chr21	10473366	A	T	UTR3	BAGE2_BAGE3_BAGE4_BAGE5	.
s2	chr21	10473402	T	C	UTR3	BAGE2_BAGE3_BAGE4_BAGE5	.
s2	chr21	10473490	A	G	UTR3	BAGE2_BAGE3_BAGE4_BAGE5	.
s2	chr21	44574137	C	T	exonic	KRTAP10-4	nonsynonymous_SNV
s2	chr22	10685774	G	A	intergenic	NONE_LOC102723780	.
s2	chr22	10742085	C	G	intergenic	NONE_LOC102723780	.
s2	chr22	10752758	A	T	intergenic	NONE_LOC102723780	.
s2	chr22	10752820	C	T	intergenic	NONE_LOC102723780	.
s2	chr22	10752912	C	T	intergenic	NONE_LOC102723780	.
s2	chr22	10752930	A	C	intergenic	NONE_LOC102723780	.
s2	chr22	12625456	A	G	intergenic	LOC102723769_NONE	.
s2	chr22	18188614	A	T	ncRNA_intronic	LOC100996415	.
s2	chr22	18537293	G	A	ncRNA_exonic	PI4KAP1	.
s2	chr22	18543718	G	C	ncRNA_exonic	PI4KAP1	.
s2	chr22	21309403	G	A	ncRNA_exonic	LOC100996335	.
s2	chr22	37724556	T	C	exonic	TRIOBP	nonsynonymous_SNV
s2	chr1_KI270711v1_random	7945	T	A	intergenic	NONE_NONE	.
s2	chr1_KI270711v1_random	8083	G	A	intergenic	NONE_NONE	.
s2	chr1_KI270711v1_random	8421	T	C	intergenic	NONE_NONE	.
s2	chr1_KI270711v1_random	8545	C	G	intergenic	NONE_NONE	.
s2	chr1_KI270711v1_random	8565	T	C	intergenic	NONE_NONE	.
s2	chr1_KI270711v1_random	8579	T	A	intergenic	NONE_NONE	.
s2	chr1_KI270711v1_random	8599	G	C	intergenic	NONE_NONE	.
s2	chr1_KI270711v1_random	9142	C	A	intergenic	NONE_NONE	.
s2	chr1_KI270711v1_random	9143	C	T	intergenic	NONE_NONE	.
s2	chr1_KI270711v1_random	9777	A	G	intergenic	NONE_NONE	.
s2	chr1_KI270711v1_random	9784	G	A	intergenic	NONE_NONE	.
s2	chr1_KI270711v1_random	9802	G	A	intergenic	NONE_NONE	.
s2	chr1_KI270711v1_random	20129	T	C	intergenic	NONE_NONE	.
s2	chr1_KI270711v1_random	23064	A	G	intergenic	NONE_NONE	.
s2	chr1_KI270711v1_random	23085	A	C	intergenic	NONE_NONE	.
s2	chr1_KI270711v1_random	23134	G	A	intergenic	NONE_NONE	.

s2	chr1_KI270713v1_random	3816	A	G	upstream	LOC102724562	.
s2	chr1_KI270713v1_random	5578	-	CC	ncRNA_exonic	LOC102724562	.
s2	chr1_KI270713v1_random	6131	C	T	ncRNA_intronic	LOC102724562	.
s2	chr9_KI270719v1_random	164997	C	G	intergenic	NONE_NONE	.
s2	chr9_KI270719v1_random	165007	G	A	intergenic	NONE_NONE	.
s2	chr9_KI270719v1_random	165011	C	T	intergenic	NONE_NONE	.
s2	chr9_KI270719v1_random	165018	C	T	intergenic	NONE_NONE	.
s2	chr9_KI270720v1_random	26184	T	C	intergenic	NONE_NONE	.
s2	chr9_KI270720v1_random	26213	-	T	intergenic	NONE_NONE	.
s2	chr9_KI270720v1_random	26218	C	-	intergenic	NONE_NONE	.
s2	chr14_GL000194v1_random	53745	C	T	ncRNA_exonic	MAFIP	.
s2	chr17_GL000205v2_random	51452	T	G	intergenic	NONE_MGC70870	.
s2	chr22_KI270733v1_random	169995	C	T	upstream	MIR3687-1_MIR3687-2	.
s2	chrUn_KI270744v1	80507	G	C	intergenic	NONE_NONE	.
s2	chrUn_KI270744v1	80511	G	C	intergenic	NONE_NONE	.
s2	chrUn_KI270744v1	80564	G	A	intergenic	NONE_NONE	.
s2	chrUn_KI270746v1	24636	C	T	intergenic	NONE_NONE	.
s2	chrUn_KI270746v1	35656	A	T	intergenic	NONE_NONE	.
s2	chrUn_KI270746v1	35670	C	T	intergenic	NONE_NONE	.
s2	chrUn_KI270746v1	36144	A	C	intergenic	NONE_NONE	.
s2	chrUn_GL000218v1	40716	C	T	ncRNA_exonic	LOC100233156	.
s2	chr3_KI270779v1_alt	150960	A	G	exonic	MUC4	nonsynonymous_SNV
s2	chr3_KI270779v1_alt	163150	A	G	exonic	MUC4	nonsynonymous_SNV
Sample	Chr	Start	Ref	Alt	Func.refGene	Gene.refGene	ExonicFunc.refGene
s3	chr1	17556	C	T	ncRNA_intronic	WASH7P	.
s3	chr1	183937	G	A	intergenic	LOC729737_MIR6859-4	.
s3	chr1	1707623	T	C	intronic	CDK11A	.
s3	chr1	16536138	C	T	upstream	LINC01783	.
s3	chr1	16565625	G	T	intronic	NBPF1	.
s3	chr1	16580670	G	A	intronic	NBPF1	.
s3	chr1	16581396	T	C	intronic	NBPF1	.
s3	chr1	16581642	A	G	intronic	NBPF1	.
s3	chr1	16583754	T	C	intronic	NBPF1	.
s3	chr1	16621877	G	A	ncRNA_intronic	CROCCP2	.
s3	chr1	16627177	C	T	ncRNA_exonic	CROCCP2	.
s3	chr1	16645695	T	C	ncRNA_intronic	MST1P2	.
s3	chr1	16645722	C	G	ncRNA_intronic	MST1P2	.
s3	chr1	16645744	G	A	ncRNA_intronic	MST1P2	.
s3	chr1	16647559	A	G	ncRNA_exonic	MST1P2	.
s3	chr1	16648263	G	A	ncRNA_exonic	MST1P2	.
s3	chr1	16648285	C	T	ncRNA_exonic	MST1P2	.
s3	chr1	16648291	G	C	ncRNA_exonic	MST1P2	.

s3	chr1	16649698	C	T	ncRNA_exonic	MST1P2	.
s3	chr1	16650155	G	A	ncRNA_exonic	MST1P2	.
s3	chr1	16650310	G	A	ncRNA_exonic	MST1P2	.
s3	chr1	16650312	C	T	ncRNA_exonic	MST1P2	.
s3	chr1	16757313	G	A	exonic	MST1L	synonymous_SNV
s3	chr1	16757321	G	A	exonic	MST1L	stopgain
s3	chr1	16759300	G	T	exonic	MST1L	synonymous_SNV
s3	chr1	16760797	A	G	exonic	MST1L	nonsynonymous_SNV
s3	chr1	16760804	T	C	exonic	MST1L	synonymous_SNV
s3	chr1	16760810	G	C	exonic	MST1L	synonymous_SNV
s3	chr1	16764407	-	T	intronic	MST1L	.
s3	chr1	22002726	C	T	intronic	CELA3A	.
s3	chr1	120810541	C	T	exonic	NBPF26	nonsynonymous_SNV
s3	chr1	144436988	C	A	exonic	NBPF15	nonsynonymous_SNV
s3	chr1	146989687	A	G	exonic	NBPF12	nonsynonymous_SNV
s3	chr1	148103850	T	C	UTR3	NBPF11	.
s3	chr1	148593522	C	T	intronic	NBPF14	.
s3	chr1	152214232	C	T	exonic	HRNR	nonsynonymous_SNV
s3	chr1	152215143	A	G	exonic	HRNR	synonymous_SNV
s3	chr1	152215642	C	T	exonic	HRNR	nonsynonymous_SNV
s3	chr1	152215843	C	T	exonic	HRNR	nonsynonymous_SNV
s3	chr1	152776551	C	T	exonic	LCE1F	synonymous_SNV
s3	chr1	201209630	G	C	exonic	IGFN1	synonymous_SNV
s3	chr1	201211524	A	G	exonic	IGFN1	nonsynonymous_SNV
s3	chr1	248061293	C	T	exonic	OR2L3	synonymous_SNV
s3	chr2	106425156	A	C	exonic	RGPD3	nonsynonymous_SNV
s3	chr2	113598529	T	C	ncRNA_intronic	WASH2P	.
s3	chr2	128318365	T	A	exonic	HS6ST1	stopgain
s3	chr2	131532842	T	C	intronic	CCDC74A	.
s3	chr2	240030223	C	T	exonic	OR6B2	synonymous_SNV
s3	chr3	75665744	G	A	exonic	FRG2C	synonymous_SNV
s3	chr4	270822	AAAAGCTTTGCCACATT(	-	UTR3	ZNF732	.
s3	chr4	9243843	C	T	upstream	USP17L17	.
s3	chr4	9248531	C	A	upstream	USP17L11_USP17L18	.
s3	chr4	9248548	G	A	upstream	USP17L11_USP17L18	.
s3	chr4	9248616	T	A	upstream	USP17L11_USP17L18	.
s3	chr4	9268602	G	A	exonic	USP17L20_USP17L22	stopgain
s3	chr5	34191075	T	A	intergenic	C1QTNF3-AMACR_NONE	.
s3	chr5	34191659	C	G	intergenic	C1QTNF3-AMACR_NONE	.
s3	chr5	34192589	A	G	intergenic	C1QTNF3-AMACR_NONE	.
s3	chr5	34193423	A	G	intergenic	C1QTNF3-AMACR_NONE	.
s3	chr5	140848888	A	G	exonic	PCDHA9	synonymous_SNV

s3	chr5	141123609	T	C	exonic	PCDHB4	synonymous_SNV
s3	chr5	141201296	A	G	exonic	PCDHB11	nonsynonymous_SNV
s3	chr6	27146790	T	C	exonic	HIST1H2BK	synonymous_SNV
s3	chr7	56373525	G	C	intergenic	NUPR2_LOC650226	.
s3	chr7	56373626	A	G	intergenic	NUPR2_LOC650226	.
s3	chr7	75148237	T	C	exonic	GTF2IRD2B	nonsynonymous_SNV
s3	chr7	100993793	G	A	exonic	MUC12	nonsynonymous_SNV
s3	chr7	100998641	C	G	exonic	MUC12	nonsynonymous_SNV
s3	chr7	101000011	G	A	exonic	MUC12	nonsynonymous_SNV
s3	chr7	101000063	G	T	exonic	MUC12	nonsynonymous_SNV
s3	chr7	101000457	G	A	exonic	MUC12	synonymous_SNV
s3	chr7	101000595	C	T	exonic	MUC12	synonymous_SNV
s3	chr7	101000597	A	C	exonic	MUC12	nonsynonymous_SNV
s3	chr7	101000612	G	A	exonic	MUC12	nonsynonymous_SNV
s3	chr7	101000614	G	A	exonic	MUC12	nonsynonymous_SNV
s3	chr7	101000626	A	G	exonic	MUC12	nonsynonymous_SNV
s3	chr7	101000633	C	T	exonic	MUC12	nonsynonymous_SNV
s3	chr7	101000638	A	G	exonic	MUC12	nonsynonymous_SNV
s3	chr7	102606000	C	T	intronic	RASA4_RASA4B	.
s3	chr7	102639154	G	T	exonic	UPK3BL1	synonymous_SNV
s3	chr7	105112793	A	C	exonic	KMT2E	synonymous_SNV
s3	chr7	105112799	A	C	exonic	KMT2E	synonymous_SNV
s3	chr7	152265049	G	T	exonic	KMT2C	stopgain
s3	chr7	152265083	C	A	exonic	KMT2C	nonsynonymous_SNV
s3	chr8	85655689	G	T	upstream	REXO1L2P	.
s3	chr8	85655705	T	G	upstream	REXO1L2P	.
s3	chr8	85655746	G	C	upstream	REXO1L2P	.
s3	chr8	85655791	G	A	upstream	REXO1L2P	.
s3	chr9	34834503	G	A	ncRNA_exonic	FAM205BP	.
s3	chr9	114323755	C	T	exonic	ORM1	synonymous_SNV
s3	chr9	128153053	CA	-	intronic	LCN2	.
s3	chr10	29490860	C	T	exonic	SVIL	nonsynonymous_SNV
s3	chr10	73675610	C	T	exonic	AGAP5	synonymous_SNV
s3	chr10	79559428	G	A	exonic	SFTPA2	nonsynonymous_SNV
s3	chr10	79611798	T	A	intronic	SFTPA1	.
s3	chr10	79611881	T	C	exonic	SFTPA1	nonsynonymous_SNV
s3	chr10	120886648	-	A	intronic	WDR11	.
s3	chr10	133625456	C	-	exonic	FRG2B	frameshift_deletion
s3	chr10	133625702	C	T	intronic	FRG2B	.
s3	chr11	18247969	T	C	exonic	SAA2_SAA2-SAA4	nonsynonymous_SNV
s3	chr12	9425638	T	A	ncRNA_intronic	DDX12P	.
s3	chr12	9429017	T	C	ncRNA_intronic	DDX12P	.

s3	chr12	31091769	C	T	exonic	DDX11	synonymous_SNV
s3	chr12	31091875	T	C	intronic	DDX11	.
s3	chr12	31091884	C	-	intronic	DDX11	.
s3	chr12	31100610	G	A	intronic	DDX11	.
s3	chr12	63823162	A	G	intergenic	TMEM5-AS1_SRGAP1	.
s3	chr13	18174070	C	T	intergenic	NONE_FAM230C	.
s3	chr14	19678529	A	T	intergenic	POTEG_OR11H2	.
s3	chr14	19678610	A	C	intergenic	POTEG_OR11H2	.
s3	chr14	19713214	G	T	exonic	OR11H2	nonsynonymous_SNV
s3	chr14	21634344	A	G	exonic	OR10G2	synonymous_SNV
s3	chr14	104951388	C	G	exonic	AHNAK2	nonsynonymous_SNV
s3	chr15	74070868	G	A	UTR3	GOLGA6A	.
s3	chr15	74071890	C	T	intronic	GOLGA6A	.
s3	chr15	74071891	A	G	intronic	GOLGA6A	.
s3	chr15	88855594	C	T	exonic	ACAN	synonymous_SNV
s3	chr15	101753899	A	G	intergenic	LOC100128108_OR4F6	.
s3	chr15	101757294	G	A	intergenic	LOC100128108_OR4F6	.
s3	chr15	101757405	T	G	intergenic	LOC100128108_OR4F6	.
s3	chr15	101761203	T	C	intergenic	LOC100128108_OR4F6	.
s3	chr15	101772004	A	G	intergenic	LOC100128108_OR4F6	.
s3	chr16	8764844	CA	-	intronic	ABAT	.
s3	chr16	12203329	C	T	intronic	SNX29	.
s3	chr16	22533964	G	C	exonic	NPIPB5	synonymous_SNV
s3	chr16	22534751	G	A	exonic	NPIPB5	nonsynonymous_SNV
s3	chr16	28723438	G	A	intronic	EIF3C_EIF3CL	.
s3	chr16	33741748	G	A	intergenic	LOC390705_ENPP7P13	.
s3	chr16	74409592	C	T	exonic	CLEC18B	nonsynonymous_SNV
s3	chr16	74409770	T	C	intronic	CLEC18B	.
s3	chr17	18492817	T	C	exonic	LGALS9C	synonymous_SNV
s3	chr17	18492997	A	G	intronic	LGALS9C	.
s3	chr17	18778850	T	C	exonic	FBXW10	nonsynonymous_SNV
s3	chr17	20589700	C	A	intergenic	CDRT15L2_LINC02088	.
s3	chr17	21415458	G	A	exonic	KCNJ12	nonsynonymous_SNV
s3	chr17	21415461	G	A	exonic	KCNJ12	nonsynonymous_SNV
s3	chr18	47686	C	T	intergenic	LOC102723376_ROCK1P1	.
s3	chr18	48137	C	T	intergenic	LOC102723376_ROCK1P1	.
s3	chr18	11644659	T	C	intergenic	SLC35G4_MIR7153	.
s3	chr19	39901935	G	A	exonic	FCGBP	nonsynonymous_SNV
s3	chr19	40848701	G	T	exonic	CYP2A6	synonymous_SNV
s3	chr19	40941364	A	G	ncRNA_exonic	CYP2B7P	.
s3	chr19	49969964	T	C	exonic	SIGLEC16	nonsynonymous_SNV
s3	chr20	28565360	C	T	intergenic	NONE_FRG1CP	.

s3	chr20	28565367	C	T	intergenic	NONE_FRG1CP	.
s3	chr20	29079212	A	G	intergenic	FRG1CP_FRG1DP	.
s3	chr20	29094411	C	T	ncRNA_exonic	FRG1DP	.
s3	chr20	29496310	T	C	ncRNA_intronic	FRG1EP	.
s3	chr20	30815497	G	C	intergenic	FRG1BP_DEFB115	.
s3	chr21	9068586	A	G	ncRNA_exonic	TEKT4P2	.
s3	chr21	9068589	G	A	ncRNA_exonic	TEKT4P2	.
s3	chr21	10462834	A	G	splicing	BAGE2_BAGE3_BAGE4_BAGE5	.
s3	chr21	10462836	C	T	exonic	BAGE2_BAGE3	stopgain
s3	chr21	10462861	G	A	exonic	BAGE2_BAGE3	nonsynonymous_SNV
s3	chr21	10473155	T	C	UTR3	BAGE2_BAGE3_BAGE4_BAGE5	.
s3	chr21	10473245	G	A	UTR3	BAGE2_BAGE3_BAGE4_BAGE5	.
s3	chr21	10473366	A	T	UTR3	BAGE2_BAGE3_BAGE4_BAGE5	.
s3	chr21	10473402	T	C	UTR3	BAGE2_BAGE3_BAGE4_BAGE5	.
s3	chr21	10473490	A	G	UTR3	BAGE2_BAGE3_BAGE4_BAGE5	.
s3	chr21	44592464	G	A	exonic	KRTAP10-6	synonymous_SNV
s3	chr21	44592470	G	A	exonic	KRTAP10-6	synonymous_SNV
s3	chr22	10685774	G	A	intergenic	NONE_LOC102723780	.
s3	chr22	10742085	C	G	intergenic	NONE_LOC102723780	.
s3	chr22	10752912	C	T	intergenic	NONE_LOC102723780	.
s3	chr22	18188614	A	T	ncRNA_intronic	LOC100996415	.
s3	chr22	18751011	G	A	ncRNA_intronic	LINC01662	.
s3	chr22	20711357	A	G	exonic	PI4KA	synonymous_SNV
s3	chr22	37724422	T	C	exonic	TRIOBP	synonymous_SNV
s3	chr22	37724619	G	A	exonic	TRIOBP	nonsynonymous_SNV
s3	chr22	50530398	AG	-	upstream_downstream	TYMP_ODF3B	.
s3	chr1_KI270711v1_random	7945	T	A	intergenic	NONE_NONE	.
s3	chr1_KI270711v1_random	8083	G	A	intergenic	NONE_NONE	.
s3	chr1_KI270711v1_random	8545	C	G	intergenic	NONE_NONE	.
s3	chr1_KI270711v1_random	8565	T	C	intergenic	NONE_NONE	.
s3	chr1_KI270711v1_random	8579	T	A	intergenic	NONE_NONE	.
s3	chr1_KI270711v1_random	8599	G	C	intergenic	NONE_NONE	.
s3	chr1_KI270711v1_random	9142	C	A	intergenic	NONE_NONE	.
s3	chr1_KI270711v1_random	9143	C	T	intergenic	NONE_NONE	.
s3	chr1_KI270711v1_random	9777	A	G	intergenic	NONE_NONE	.
s3	chr1_KI270711v1_random	9784	G	A	intergenic	NONE_NONE	.
s3	chr1_KI270711v1_random	23064	A	G	intergenic	NONE_NONE	.
s3	chr1_KI270711v1_random	23085	A	C	intergenic	NONE_NONE	.
s3	chr1_KI270711v1_random	23134	G	A	intergenic	NONE_NONE	.
s3	chr1_KI270713v1_random	3816	A	G	upstream	LOC102724562	.
s3	chr1_KI270713v1_random	5578	-	CC	ncRNA_exonic	LOC102724562	.
s3	chr1_KI270713v1_random	32494	C	T	ncRNA_exonic	LOC440570	.

s3	chr1_KI270713v1_random	32593	G	A	ncRNA_exonic	LOC440570	.
s3	chr3_GL000221v1_random	14983	A	G	intergenic	NONE_NONE	.
s3	chr9_KI270719v1_random	164997	C	G	intergenic	NONE_NONE	.
s3	chr9_KI270719v1_random	165007	G	A	intergenic	NONE_NONE	.
s3	chr9_KI270719v1_random	165011	C	T	intergenic	NONE_NONE	.
s3	chr9_KI270719v1_random	165018	C	T	intergenic	NONE_NONE	.
s3	chr9_KI270720v1_random	5153	T	C	intergenic	NONE_NONE	.
s3	chr9_KI270720v1_random	5162	G	C	intergenic	NONE_NONE	.
s3	chr9_KI270720v1_random	26080	G	T	intergenic	NONE_NONE	.
s3	chr9_KI270720v1_random	26184	T	C	intergenic	NONE_NONE	.
s3	chr14_GL000194v1_random	53745	C	T	ncRNA_exonic	MAFIP	.
s3	chr16_KI270728v1_random	1580019	G	A	intergenic	ENPP7P13_NONE	.
s3	chr17_GL000205v2_random	51452	T	G	intergenic	NONE_MGC70870	.
s3	chr17_GL000205v2_random	51492	A	G	intergenic	NONE_MGC70870	.
s3	chr17_GL000205v2_random	51620	C	A	intergenic	NONE_MGC70870	.
s3	chr22_KI270733v1_random	169850	-	GC(	upstream	MIR3687-1_MIR3687-2	.
s3	chrUn_GL000220v1	142182	G	A	intergenic	LOC100507412_MIR6724-4	.
s3	chrUn_KI270744v1	80564	G	A	intergenic	NONE_NONE	.
s3	chrUn_KI270746v1	24636	C	T	intergenic	NONE_NONE	.
s3	chrUn_KI270746v1	24641	C	T	intergenic	NONE_NONE	.
s3	chrUn_KI270746v1	35656	A	T	intergenic	NONE_NONE	.
s3	chrUn_KI270746v1	35670	C	T	intergenic	NONE_NONE	.
s3	chrUn_KI270746v1	35774	A	G	intergenic	NONE_NONE	.
s3	chrUn_GL000218v1	40716	C	T	ncRNA_exonic	LOC100233156	.
Sample	Chr	Start	Ref	Alt	Func.refGene	Gene.refGene	ExonicFunc.refGene
s4	chr1	17556	C	T	ncRNA_intronic	WASH7P	.
s4	chr1	183937	G	A	intergenic	LOC729737_MIR6859-4	.
s4	chr1	1495915	T	C	UTR3	ATAD3B	.
s4	chr1	12893329	C	G	exonic	PRAMEF10	nonsynonymous_SNV
s4	chr1	12941422	T	C	exonic	PRAMEF6	nonsynonymous_SNV
s4	chr1	16536138	C	T	upstream	LINC01783	.
s4	chr1	16565461	C	T	intronic	NBPF1	.
s4	chr1	16580670	G	A	intronic	NBPF1	.
s4	chr1	16583754	T	C	intronic	NBPF1	.
s4	chr1	16621877	G	A	ncRNA_intronic	CROCCP2	.
s4	chr1	16627177	C	T	ncRNA_exonic	CROCCP2	.
s4	chr1	16645695	T	C	ncRNA_intronic	MST1P2	.
s4	chr1	16645722	C	G	ncRNA_intronic	MST1P2	.
s4	chr1	16645744	G	A	ncRNA_intronic	MST1P2	.
s4	chr1	16646924	G	A	ncRNA_intronic	MST1P2	.
s4	chr1	16647559	A	G	ncRNA_exonic	MST1P2	.
s4	chr1	16648250	G	A	ncRNA_exonic	MST1P2	.

s4	chr1	16648263	G	A	ncRNA_exonic	MST1P2	.
s4	chr1	16648285	C	T	ncRNA_exonic	MST1P2	.
s4	chr1	16648291	G	C	ncRNA_exonic	MST1P2	.
s4	chr1	16648783	-	CC	ncRNA_exonic	MST1P2	.
s4	chr1	16649698	C	T	ncRNA_exonic	MST1P2	.
s4	chr1	16650155	G	A	ncRNA_exonic	MST1P2	.
s4	chr1	16757313	G	A	exonic	MST1L	synonymous_SNV
s4	chr1	16757393	C	T	exonic	MST1L	nonsynonymous_SNV
s4	chr1	16759300	G	T	exonic	MST1L	synonymous_SNV
s4	chr1	143499053	G	T	intergenic	LOC645166_RNVU1-17	.
s4	chr1	146989666	T	G	exonic	NBPF12	nonsynonymous_SNV
s4	chr1	146989687	A	G	exonic	NBPF12	nonsynonymous_SNV
s4	chr1	146989731	G	C	exonic	NBPF12	nonsynonymous_SNV
s4	chr1	152214232	C	T	exonic	HRNR	nonsynonymous_SNV
s4	chr1	152214433	C	T	exonic	HRNR	nonsynonymous_SNV
s4	chr1	152215143	A	G	exonic	HRNR	synonymous_SNV
s4	chr1	152215642	C	T	exonic	HRNR	nonsynonymous_SNV
s4	chr1	152215843	C	T	exonic	HRNR	nonsynonymous_SNV
s4	chr1	152776551	C	T	exonic	LCE1F	synonymous_SNV
s4	chr1	248061187	A	G	exonic	OR2L3	nonsynonymous_SNV
s4	chr1	248061267	G	T	exonic	OR2L3	nonsynonymous_SNV
s4	chr1	248061293	C	T	exonic	OR2L3	synonymous_SNV
s4	chr2	95945265	A	T	intronic	ANKRD36C	.
s4	chr2	112390163	C	T	exonic	RGPD5_RGPD8	nonsynonymous_SNV
s4	chr2	131252968	T	C	exonic	POTEE	synonymous_SNV
s4	chr3	75630958	G	T	downstream	MIR1324	.
s4	chr3	75665744	G	A	exonic	FRG2C	synonymous_SNV
s4	chr4	9243843	C	T	upstream	USP17L17	.
s4	chr4	9248548	G	A	upstream	USP17L11_USP17L18	.
s4	chr4	9248616	T	A	upstream	USP17L11_USP17L18	.
s4	chr4	9268602	G	A	exonic	USP17L20_USP17L22	stopgain
s4	chr5	34191659	C	G	intergenic	C1QTNF3-AMACR_NONE	.
s4	chr5	34193423	A	G	intergenic	C1QTNF3-AMACR_NONE	.
s4	chr5	140848888	A	G	exonic	PCDHA9	synonymous_SNV
s4	chr5	141201296	A	G	exonic	PCDHB11	nonsynonymous_SNV
s4	chr7	73225850	A	C	ncRNA_intronic	NCF1B	.
s4	chr7	100993793	G	A	exonic	MUC12	nonsynonymous_SNV
s4	chr7	101000011	G	A	exonic	MUC12	nonsynonymous_SNV
s4	chr7	101000063	G	T	exonic	MUC12	nonsynonymous_SNV
s4	chr7	101000457	G	A	exonic	MUC12	synonymous_SNV
s4	chr7	101000612	G	A	exonic	MUC12	nonsynonymous_SNV
s4	chr7	101000614	G	A	exonic	MUC12	nonsynonymous_SNV

s4	chr7	101000633	C	T	exonic	MUC12	nonsynonymous_SNV
s4	chr7	152265049	G	T	exonic	KMT2C	stopgain
s4	chr7	152265083	C	A	exonic	KMT2C	nonsynonymous_SNV
s4	chr8	8028407	G	A	intergenic	FAM66E_MIR548I3	.
s4	chr8	8028541	T	C	intergenic	FAM66E_MIR548I3	.
s4	chr8	85655705	T	G	upstream	REXO1L2P	.
s4	chr8	85655746	G	C	upstream	REXO1L2P	.
s4	chr9	34834503	G	A	ncRNA_exonic	FAM205BP	.
s4	chr9	62843444	C	T	ncRNA_intronic	PTGER4P2-CDK2AP2P2	.
s4	chr9	65650207	G	A	intergenic	FOXD4L5_CBWD5	.
s4	chr9	65650228	G	A	intergenic	FOXD4L5_CBWD5	.
s4	chr9	65650238	C	T	intergenic	FOXD4L5_CBWD5	.
s4	chr9	96938627	C	T	exonic	NUTM2G	synonymous_SNV
s4	chr9	114323755	C	T	exonic	ORM1	synonymous_SNV
s4	chr9	128153053	CA	-	intronic	LCN2	.
s4	chr10	73675610	C	T	exonic	AGAP5	synonymous_SNV
s4	chr10	79559428	G	A	exonic	SFTPA2	nonsynonymous_SNV
s4	chr10	79611881	T	C	exonic	SFTPA1	nonsynonymous_SNV
s4	chr10	133625387	T	C	exonic	FRG2B	synonymous_SNV
s4	chr10	133625456	C	-	exonic	FRG2B	frameshift_deletion
s4	chr10	133625511	T	C	exonic	FRG2B	nonsynonymous_SNV
s4	chr11	124023094	A	G	exonic	OR10G9	nonsynonymous_SNV
s4	chr12	9425638	T	A	ncRNA_intronic	DDX12P	.
s4	chr12	9429017	T	C	ncRNA_intronic	DDX12P	.
s4	chr12	31091769	C	T	exonic	DDX11	synonymous_SNV
s4	chr12	31091875	T	C	intronic	DDX11	.
s4	chr12	31091884	C	-	intronic	DDX11	.
s4	chr12	31100610	G	A	intronic	DDX11	.
s4	chr12	63823162	A	G	intergenic	TMEM5-AS1_SRGAP1	.
s4	chr13	23892093	T	C	UTR3	PCOTH	.
s4	chr13	25097260	C	T	exonic	PABPC3	synonymous_SNV
s4	chr14	19402323	G	A	downstream	POTEG	.
s4	chr14	19414622	C	A	intronic	POTEG	.
s4	chr14	19678529	A	T	intergenic	POTEG_OR11H2	.
s4	chr14	19679029	G	A	intergenic	POTEG_OR11H2	.
s4	chr14	104951388	C	G	exonic	AHNAK2	nonsynonymous_SNV
s4	chr15	20408783	-	T	ncRNA_intronic	HERC2P3	.
s4	chr15	74070868	G	A	UTR3	GOLGA6A	.
s4	chr15	101761203	T	C	intergenic	LOC100128108_OR4F6	.
s4	chr15	101772004	A	G	intergenic	LOC100128108_OR4F6	.
s4	chr16	12203303	T	C	intronic	SNX29	.
s4	chr16	28723438	G	A	intronic	EIF3C_EIF3CL	.

s4	chr16	67195891	CAG	-	exonic	E2F4	nonframeshift_deletion
s4	chr16	70941764	T	C	exonic	HYDIN	nonsynonymous_SNV
s4	chr16	74391380	T	C	intronic	NPIPB15	.
s4	chr16	74391416	G	A	exonic	NPIPB15	nonsynonymous_SNV
s4	chr16	74409592	C	T	exonic	CLEC18B	nonsynonymous_SNV
s4	chr16	74409652	G	T	intronic	CLEC18B	.
s4	chr16	74409770	T	C	intronic	CLEC18B	.
s4	chr17	15619445	A	T	exonic	CDRT1	nonsynonymous_SNV
s4	chr17	18492817	T	C	exonic	LGALS9C	synonymous_SNV
s4	chr17	18778850	T	C	exonic	FBXW10	nonsynonymous_SNV
s4	chr17	21415458	G	A	exonic	KCNJ12	nonsynonymous_SNV
s4	chr17	21415461	G	A	exonic	KCNJ12	nonsynonymous_SNV
s4	chr18	47686	C	T	intergenic	LOC102723376_ROCK1P1	.
s4	chr18	48191	G	C	intergenic	LOC102723376_ROCK1P1	.
s4	chr19	12392184	C	T	exonic	ZNF799	nonsynonymous_SNV
s4	chr19	21569093	G	A	intergenic	ZNF429_LOC400682	.
s4	chr19	39886240	T	C	exonic	FCGBP	nonsynonymous_SNV
s4	chr19	40850014	A	C	intronic	CYP2A6	.
s4	chr19	40850019	T	C	intronic	CYP2A6	.
s4	chr19	49044175	A	G	UTR5	CGB5	.
s4	chr19	49044189	G	C	UTR5	CGB5	.
s4	chr19	49969964	T	C	exonic	SIGLEC16	nonsynonymous_SNV
s4	chr19	52915302	A	G	exonic	ZNF888	synonymous_SNV
s4	chr21	8420440	GT	-	intergenic	LOC100507412_MIR6724-4	.
s4	chr21	9068586	A	G	ncRNA_exonic	TEKT4P2	.
s4	chr21	10463097	T	A	intronic	BAGE2_BAGE3_BAGE4_BAGE5	.
s4	chr21	10473366	A	T	UTR3	BAGE2_BAGE3_BAGE4_BAGE5	.
s4	chr21	10473402	T	C	UTR3	BAGE2_BAGE3_BAGE4_BAGE5	.
s4	chr21	10473490	A	G	UTR3	BAGE2_BAGE3_BAGE4_BAGE5	.
s4	chr22	10742085	C	G	intergenic	NONE_LOC102723780	.
s4	chr22	12625456	A	G	intergenic	LOC102723769_NONE	.
s4	chr22	15528255	T	A	exonic	OR11H1	nonsynonymous_SNV
s4	chr22	24657425	G	A	ncRNA_exonic	POM121L10P	.
s4	chr22	37724422	T	C	exonic	TRIOBP	synonymous_SNV
s4	chr1_KI270711v1_random	7945	T	A	intergenic	NONE_NONE	.
s4	chr1_KI270711v1_random	8083	G	A	intergenic	NONE_NONE	.
s4	chr1_KI270711v1_random	8545	C	G	intergenic	NONE_NONE	.
s4	chr1_KI270711v1_random	8565	T	C	intergenic	NONE_NONE	.
s4	chr1_KI270711v1_random	8579	T	A	intergenic	NONE_NONE	.
s4	chr1_KI270711v1_random	9142	C	A	intergenic	NONE_NONE	.
s4	chr1_KI270711v1_random	9143	C	T	intergenic	NONE_NONE	.
s4	chr1_KI270711v1_random	9784	G	A	intergenic	NONE_NONE	.

s4	chr1_KI270711v1_random	23064	A	G	intergenic	NONE_NONE	.
s4	chr1_KI270711v1_random	23085	A	C	intergenic	NONE_NONE	.
s4	chr1_KI270711v1_random	24416	C	T	intergenic	NONE_NONE	.
s4	chr1_KI270713v1_random	3816	A	G	upstream	LOC102724562	.
s4	chr1_KI270713v1_random	5578	-	CC	ncRNA_exonic	LOC102724562	.
s4	chr1_KI270713v1_random	6131	C	T	ncRNA_intronic	LOC102724562	.
s4	chr9_KI270719v1_random	164997	C	G	intergenic	NONE_NONE	.
s4	chr9_KI270719v1_random	165007	G	A	intergenic	NONE_NONE	.
s4	chr9_KI270720v1_random	5153	T	C	intergenic	NONE_NONE	.
s4	chr9_KI270720v1_random	5162	G	C	intergenic	NONE_NONE	.
s4	chr9_KI270720v1_random	26184	T	C	intergenic	NONE_NONE	.
s4	chr9_KI270720v1_random	26218	C	-	intergenic	NONE_NONE	.
s4	chr14_GL000194v1_random	53745	C	T	ncRNA_exonic	MAFIP	.
s4	chr17_GL000205v2_random	51492	A	G	intergenic	NONE_MGC70870	.
s4	chr17_GL000205v2_random	51620	C	A	intergenic	NONE_MGC70870	.
s4	chr22_KI270733v1_random	169995	C	T	upstream	MIR3687-1_MIR3687-2	.
s4	chrUn_GL000220v1	142182	G	A	intergenic	LOC100507412_MIR6724-4	.
s4	chrUn_KI270744v1	80564	G	A	intergenic	NONE_NONE	.
s4	chrUn_KI270746v1	35656	A	T	intergenic	NONE_NONE	.
s4	chrUn_KI270746v1	35670	C	T	intergenic	NONE_NONE	.
s4	chrUn_GL000218v1	40716	C	T	ncRNA_exonic	LOC100233156	.
s4	chr3_KI270779v1_alt	163540	CC	-	exonic	MUC4	frameshift_deletion
s4	chr3_KI270779v1_alt	163543	-	TC	exonic	MUC4	frameshift_insertion
s4	chr3_KI270779v1_alt	163564	T	C	exonic	MUC4	nonsynonymous_SNV
Sample	Chr	Start	Ref	Alt	Func.refGene	Gene.refGene	ExonicFunc.refGene
s6	chr1	1486536	T	C	intronic	ATAD3B	.
s6	chr1	13319449	A	G	exonic	PRAMEF15	nonsynonymous_SNV
s6	chr1	13319482	A	C	exonic	PRAMEF15	nonsynonymous_SNV
s6	chr1	16536138	C	T	upstream	LINC01783	.
s6	chr1	16565625	G	T	intronic	NBPF1	.
s6	chr1	16580670	G	A	intronic	NBPF1	.
s6	chr1	16581396	T	C	intronic	NBPF1	.
s6	chr1	16581539	C	G	intronic	NBPF1	.
s6	chr1	16645595	G	A	ncRNA_exonic	MST1P2	.
s6	chr1	16648250	G	A	ncRNA_exonic	MST1P2	.
s6	chr1	16648263	G	A	ncRNA_exonic	MST1P2	.
s6	chr1	16648285	C	T	ncRNA_exonic	MST1P2	.
s6	chr1	16648291	G	C	ncRNA_exonic	MST1P2	.
s6	chr1	16649698	C	T	ncRNA_exonic	MST1P2	.
s6	chr1	16650155	G	A	ncRNA_exonic	MST1P2	.
s6	chr1	16758917	C	T	exonic	MST1L	nonsynonymous_SNV
s6	chr1	16760797	A	G	exonic	MST1L	nonsynonymous_SNV

s6	chr1	16760804	T	C	exonic	MST1L	synonymous_SNV
s6	chr1	16760810	G	C	exonic	MST1L	synonymous_SNV
s6	chr1	22002726	C	T	intronic	CELA3A	.
s6	chr1	146989666	T	G	exonic	NBPF12	nonsynonymous_SNV
s6	chr1	146989687	A	G	exonic	NBPF12	nonsynonymous_SNV
s6	chr1	146989731	G	C	exonic	NBPF12	nonsynonymous_SNV
s6	chr1	149080154	C	G	exonic	NBPF9	nonsynonymous_SNV
s6	chr1	152110008	G	T	exonic	TCHH	nonsynonymous_SNV
s6	chr1	152214232	C	T	exonic	HRNR	nonsynonymous_SNV
s6	chr1	152214326	T	C	exonic	HRNR	nonsynonymous_SNV
s6	chr1	152215143	A	G	exonic	HRNR	synonymous_SNV
s6	chr1	152215642	C	T	exonic	HRNR	nonsynonymous_SNV
s6	chr1	152215843	C	T	exonic	HRNR	nonsynonymous_SNV
s6	chr1	152216231	T	A	exonic	HRNR	nonsynonymous_SNV
s6	chr1	152216371	A	G	exonic	HRNR	nonsynonymous_SNV
s6	chr1	154607873	AC	-	intronic	ADAR	.
s6	chr2	95938785	T	G	intronic	ANKRD36C	.
s6	chr2	95945265	A	T	intronic	ANKRD36C	.
s6	chr2	112390163	C	T	exonic	RGPD5_RGPD8	nonsynonymous_SNV
s6	chr2	128318365	T	A	exonic	HS6ST1	stopgain
s6	chr2	131264056	G	T	exonic	POTEE	nonsynonymous_SNV
s6	chr2	131491116	G	A	intronic	MZT2A	.
s6	chr2	232381443	C	T	intronic	ALPP	.
s6	chr3	75665231	G	T	intronic	FRG2C	.
s6	chr3	75665237	G	T	intronic	FRG2C	.
s6	chr3	75665744	G	A	exonic	FRG2C	synonymous_SNV
s6	chr3	75669294	G	A	intergenic	FRG2C_LINC00960	.
s6	chr3	75669309	C	G	intergenic	FRG2C_LINC00960	.
s6	chr3	75669370	C	T	intergenic	FRG2C_LINC00960	.
s6	chr3	195619990	T	C	intergenic	APOD_LOC105374297	.
s6	chr4	9248616	T	A	upstream	USP17L11_USP17L18	.
s6	chr4	9268602	G	A	exonic	USP17L20_USP17L22	stopgain
s6	chr4	9368818	T	C	ncRNA_exonic	USP17L6P	.
s6	chr5	34191075	T	A	intergenic	C1QTNF3-AMACR_NONE	.
s6	chr5	34192589	A	G	intergenic	C1QTNF3-AMACR_NONE	.
s6	chr5	34193423	A	G	intergenic	C1QTNF3-AMACR_NONE	.
s6	chr5	141201296	A	G	exonic	PCDHB11	nonsynonymous_SNV
s6	chr5	170080127	TG	-	intronic	DOCK2	.
s6	chr7	5958978	G	A	intronic	RSPH10B_RSPH10B2	.
s6	chr7	38358446	T	C	ncRNA_intronic	TRG-AS1	.
s6	chr7	73226001	T	C	ncRNA_intronic	NCF1B	.
s6	chr7	75440925	C	T	intronic	POM121C	.

s6	chr7	100995392	G	T	exonic	MUC12	nonsynonymous_SNV
s6	chr7	100998641	C	G	exonic	MUC12	nonsynonymous_SNV
s6	chr7	100998937	G	A	exonic	MUC12	nonsynonymous_SNV
s6	chr7	101000011	G	A	exonic	MUC12	nonsynonymous_SNV
s6	chr7	101000063	G	T	exonic	MUC12	nonsynonymous_SNV
s6	chr7	101000457	G	A	exonic	MUC12	synonymous_SNV
s6	chr7	101000595	C	T	exonic	MUC12	synonymous_SNV
s6	chr7	102639154	G	T	exonic	UPK3BL1	synonymous_SNV
s6	chr8	85655689	G	T	upstream	REXO1L2P	.
s6	chr8	85655705	T	G	upstream	REXO1L2P	.
s6	chr8	85655709	C	A	upstream	REXO1L2P	.
s6	chr8	85655746	G	C	upstream	REXO1L2P	.
s6	chr8	100709611	C	A	exonic	PABPC1	nonsynonymous_SNV
s6	chr9	34833000	C	T	ncRNA_exonic	FAM205BP	.
s6	chr9	34834503	G	A	ncRNA_exonic	FAM205BP	.
s6	chr9	62801506	G	T	ncRNA_exonic	LINC01410	.
s6	chr9	65737280	G	A	exonic	FOXD4L4	synonymous_SNV
s6	chr9	96938627	C	T	exonic	NUTM2G	synonymous_SNV
s6	chr9	114323755	C	T	exonic	ORM1	synonymous_SNV
s6	chr9	122724407	T	C	exonic	OR1L4	nonsynonymous_SNV
s6	chr9	122724418	A	G	exonic	OR1L4	synonymous_SNV
s6	chr9	128153053	CA	-	intronic	LCN2	.
s6	chr9	138175895	C	T	ncRNA_exonic	TUBBP5	.
s6	chr9	138176968	G	A	ncRNA_exonic	TUBBP5	.
s6	chr10	48010538	G	A	ncRNA_exonic	AGAP12P	.
s6	chr10	79559428	G	A	exonic	SFTPA2	nonsynonymous_SNV
s6	chr10	79611798	T	A	intronic	SFTPA1	.
s6	chr11	1187040	T	C	exonic	MUC5AC	synonymous_SNV
s6	chr11	4587032	A	C	exonic	OR52I2	nonsynonymous_SNV
s6	chr11	18247969	T	C	exonic	SAA2_SAA2-SAA4	nonsynonymous_SNV
s6	chr11	64315749	G	C	exonic	ESRRA	nonsynonymous_SNV
s6	chr11	71538425	A	T	exonic	KRTAP5-8	nonsynonymous_SNV
s6	chr12	9421465	G	-	ncRNA_exonic	DDX12P	.
s6	chr12	9425638	T	A	ncRNA_intronic	DDX12P	.
s6	chr12	9429017	T	C	ncRNA_intronic	DDX12P	.
s6	chr12	9434055	T	C	ncRNA_intronic	DDX12P	.
s6	chr12	40487270	A	G	exonic	MUC19	unknown
s6	chr12	40487284	G	T	exonic	MUC19	unknown
s6	chr12	40487326	C	A	exonic	MUC19	unknown
s6	chr12	52305249	G	A	exonic	KRT86	nonsynonymous_SNV
s6	chr12	52305260	C	G	exonic	KRT86	synonymous_SNV
s6	chr13	18177976	A	T	intergenic	NONE_FAM230C	.

s6	chr13	18177985	-	A	intergenic	NONE_FAM230C	.
s6	chr14	19402323	G	A	downstream	POTEG	.
s6	chr14	19713112	G	A	exonic	OR11H2	nonsynonymous_SNV
s6	chr14	19713115	T	C	exonic	OR11H2	nonsynonymous_SNV
s6	chr15	20261847	A	G	intergenic	NONE_CHEK2P2	.
s6	chr15	74071890	C	T	intronic	GOLGA6A	.
s6	chr15	74071891	A	G	intronic	GOLGA6A	.
s6	chr15	101755290	A	G	intergenic	LOC100128108_OR4F6	.
s6	chr15	101757294	G	A	intergenic	LOC100128108_OR4F6	.
s6	chr15	101760080	G	A	intergenic	LOC100128108_OR4F6	.
s6	chr15	101761203	T	C	intergenic	LOC100128108_OR4F6	.
s6	chr15	101772004	A	G	intergenic	LOC100128108_OR4F6	.
s6	chr16	28723438	G	A	intronic	EIF3C_EIF3CL	.
s6	chr16	29383753	A	G	exonic	NPIPB11	synonymous_SNV
s6	chr16	33741748	G	A	intergenic	LOC390705_ENPP7P13	.
s6	chr16	70120825	T	C	intronic	PDPR	.
s6	chr16	70131836	C	T	intronic	PDPR	.
s6	chr16	70131846	T	C	intronic	PDPR	.
s6	chr16	74391416	G	A	exonic	NPIPB15	nonsynonymous_SNV
s6	chr16	74409592	C	T	exonic	CLEC18B	nonsynonymous_SNV
s6	chr16	74409652	G	T	intronic	CLEC18B	.
s6	chr16	74409770	T	C	intronic	CLEC18B	.
s6	chr16	74418033	G	A	intronic	CLEC18B	.
s6	chr17	18492817	T	C	exonic	LGALS9C	synonymous_SNV
s6	chr17	18492905	-	AG	intronic	LGALS9C	.
s6	chr17	20589700	C	A	intergenic	CDRT15L2_LINC02088	.
s6	chr17	21415960	C	T	exonic	KCNJ12	synonymous_SNV
s6	chr17	21703029	C	G	exonic	KCNJ18	synonymous_SNV
s6	chr17	21703083	C	T	exonic	KCNJ18	synonymous_SNV
s6	chr17	21703101	C	T	exonic	KCNJ18	synonymous_SNV
s6	chr17	60211060	C	T	exonic	USP32	nonsynonymous_SNV
s6	chr18	46969231	C	A	intronic	KATNAL2	.
s6	chr19	35011449	CT	-	intronic	GRAMD1A	.
s6	chr19	39877736	A	C	exonic	FCGBP	synonymous_SNV
s6	chr19	40850019	T	C	intronic	CYP2A6	.
s6	chr19	49958551	G	T	exonic	SIGLEC11	synonymous_SNV
s6	chr19	49969964	T	C	exonic	SIGLEC16	nonsynonymous_SNV
s6	chr19	53375790	T	C	exonic	ZNF525	synonymous_SNV
s6	chr20	29079212	A	G	intergenic	FRG1CP_FRG1DP	.
s6	chr20	29415781	T	G	ncRNA_intronic	FRG2EP	.
s6	chr20	29415796	A	C	ncRNA_intronic	FRG2EP	.
s6	chr21	8208471	G	C	ncRNA_exonic	RNA45SN2	.

s6	chr21	8214799	-	C	ncRNA_exonic	RNA28SN2_RNA45SN2	.
s6	chr21	9068586	A	G	ncRNA_exonic	TEKT4P2	.
s6	chr21	9068589	G	A	ncRNA_exonic	TEKT4P2	.
s6	chr21	10397827	-	T	intergenic	LINC01667_BAGE	.
s6	chr21	10462865	G	A	exonic	BAGE2_BAGE3	synonymous_SNV
s6	chr21	10462873	G	A	exonic	BAGE2_BAGE3	nonsynonymous_SNV
s6	chr21	10462962	G	T	UTR3	BAGE2_BAGE3_BAGE4_BAGE5	.
s6	chr21	10463002	C	T	UTR3	BAGE2_BAGE3_BAGE4_BAGE5	.
s6	chr21	10473366	A	T	UTR3	BAGE2_BAGE3_BAGE4_BAGE5	.
s6	chr21	10473490	A	G	UTR3	BAGE2_BAGE3_BAGE4_BAGE5	.
s6	chr21	10473548	C	A	UTR3	BAGE2_BAGE3_BAGE4_BAGE5	.
s6	chr21	44369403	GGGCGCTGTGGGGAGC	-	intronic	TRPM2	.
s6	chr21	44573789	A	G	exonic	KRTAP10-4	nonsynonymous_SNV
s6	chr22	10742085	C	G	intergenic	NONE_LOC102723780	.
s6	chr22	10752820	C	A	intergenic	NONE_LOC102723780	.
s6	chr22	10752912	C	T	intergenic	NONE_LOC102723780	.
s6	chr22	15528255	T	A	exonic	OR11H1	nonsynonymous_SNV
s6	chr22	15711039	-	TC	intronic	POTEH	.
s6	chr22	18188614	A	T	ncRNA_intronic	LOC100996415	.
s6	chr22	18535866	A	G	ncRNA_exonic	PI4KAP1	.
s6	chr22	18535978	A	T	ncRNA_intronic	PI4KAP1	.
s6	chr22	18536633	T	C	ncRNA_intronic	PI4KAP1	.
s6	chr22	24260162	C	T	ncRNA_intronic	POM121L9P	.
s6	chr22	50530398	AG	-	upstream_downstream	TYMP_ODF3B	.
s6	chr1_KI270711v1_random	7809	G	T	intergenic	NONE_NONE	.
s6	chr1_KI270711v1_random	7945	T	A	intergenic	NONE_NONE	.
s6	chr1_KI270711v1_random	8083	G	A	intergenic	NONE_NONE	.
s6	chr1_KI270711v1_random	8545	C	G	intergenic	NONE_NONE	.
s6	chr1_KI270711v1_random	8565	T	C	intergenic	NONE_NONE	.
s6	chr1_KI270711v1_random	8579	T	A	intergenic	NONE_NONE	.
s6	chr1_KI270711v1_random	9142	C	A	intergenic	NONE_NONE	.
s6	chr1_KI270711v1_random	9143	C	T	intergenic	NONE_NONE	.
s6	chr1_KI270711v1_random	9802	G	A	intergenic	NONE_NONE	.
s6	chr1_KI270711v1_random	23085	A	C	intergenic	NONE_NONE	.
s6	chr1_KI270713v1_random	3164	G	A	intergenic	NONE_LOC102724562	.
s6	chr1_KI270713v1_random	3236	T	C	upstream	LOC102724562	.
s6	chr1_KI270713v1_random	3816	A	G	upstream	LOC102724562	.
s6	chr1_KI270713v1_random	4608	G	C	ncRNA_exonic	LOC102724562	.
s6	chr1_KI270713v1_random	32494	C	T	ncRNA_exonic	LOC440570	.
s6	chr1_KI270713v1_random	32593	G	A	ncRNA_exonic	LOC440570	.
s6	chr9_KI270720v1_random	26078	G	A	intergenic	NONE_NONE	.
s6	chr9_KI270720v1_random	26184	T	C	intergenic	NONE_NONE	.

s6	chr16_KI270728v1_random	1579960	A	G	intergenic	ENPP7P13_NONE	.
s6	chr22_KI270733v1_random	169995	C	T	upstream	MIR3687-1_MIR3687-2	.
s6	chrUn_GL000220v1	142182	G	A	intergenic	LOC100507412_MIR6724-4	.
s6	chrUn_KI270744v1	80564	G	A	intergenic	NONE_NONE	.
s6	chrUn_KI270746v1	35656	A	T	intergenic	NONE_NONE	.
s6	chrUn_KI270746v1	35670	C	T	intergenic	NONE_NONE	.
s6	chrUn_KI270746v1	35985	T	A	intergenic	NONE_NONE	.
s6	chrUn_GL000218v1	40556	G	A	ncRNA_intronic	LOC100233156	.
s6	chrUn_GL000218v1	40716	C	T	ncRNA_exonic	LOC100233156	.
s6	chr3_KI270779v1_alt	163541	C	G	exonic	MUC4	nonsynonymous_SNV
s6	chr11_KI270902v1_alt	81885	C	T	intronic	MUC6	.
s6	chr11_KI270902v1_alt	81892	C	G	intronic	MUC6	.
s6	chr11_KI270902v1_alt	81896	G	C	intronic	MUC6	.
s6	chr11_KI270902v1_alt	81918	G	T	intronic	MUC6	.
s6	chr11_KI270902v1_alt	81925	G	T	intronic	MUC6	.
s6	chr11_KI270902v1_alt	81936	A	C	intronic	MUC6	.
s6	chr3_KI270935v1_alt	154209	GAAGAGGGGTGGCGTG	-	exonic	MUC4	unknown
s6	chr3_KI270937v1_alt	68930	G	A	exonic	MUC20	nonsynonymous_SNV
Sample	Chr	Start	Ref	Alt	Func.refGene	Gene.refGene	ExonicFunc.refGene
s7	chr1	16536138	C	T	upstream	LINC01783	.
s7	chr1	16565625	G	T	intronic	NBPF1	.
s7	chr1	16565761	T	A	exonic	NBPF1	unknown
s7	chr1	16581396	T	C	intronic	NBPF1	.
s7	chr1	16581593	G	T	intronic	NBPF1	.
s7	chr1	16583754	T	C	intronic	NBPF1	.
s7	chr1	16645595	G	A	ncRNA_exonic	MST1P2	.
s7	chr1	16648250	G	A	ncRNA_exonic	MST1P2	.
s7	chr1	16648263	G	A	ncRNA_exonic	MST1P2	.
s7	chr1	16648285	C	T	ncRNA_exonic	MST1P2	.
s7	chr1	16648291	G	C	ncRNA_exonic	MST1P2	.
s7	chr1	16648783	-	CC	ncRNA_exonic	MST1P2	.
s7	chr1	16649698	C	T	ncRNA_exonic	MST1P2	.
s7	chr1	16650155	G	A	ncRNA_exonic	MST1P2	.
s7	chr1	16757393	C	T	exonic	MST1L	nonsynonymous_SNV
s7	chr1	16760797	A	G	exonic	MST1L	nonsynonymous_SNV
s7	chr1	16760804	T	C	exonic	MST1L	synonymous_SNV
s7	chr1	16760810	G	C	exonic	MST1L	synonymous_SNV
s7	chr1	120814068	C	T	intronic	NBPF26	.
s7	chr1	146989666	T	G	exonic	NBPF12	nonsynonymous_SNV
s7	chr1	146989687	A	G	exonic	NBPF12	nonsynonymous_SNV
s7	chr1	146989731	G	C	exonic	NBPF12	nonsynonymous_SNV
s7	chr1	152155485	C	T	exonic	RPTN	nonsynonymous_SNV

s7	chr1	152214232	C	T	exonic	HRNR	nonsynonymous_SNV
s7	chr1	152215143	A	G	exonic	HRNR	synonymous_SNV
s7	chr1	152215642	C	T	exonic	HRNR	nonsynonymous_SNV
s7	chr1	152215843	C	T	exonic	HRNR	nonsynonymous_SNV
s7	chr1	152216231	T	A	exonic	HRNR	nonsynonymous_SNV
s7	chr1	152216237	ATCC	-	exonic	HRNR	frameshift_deletion
s7	chr1	152216371	A	G	exonic	HRNR	nonsynonymous_SNV
s7	chr1	154607873	AC	-	intronic	ADAR	.
s7	chr2	89117499	G	A	intergenic	MIR4436A_LOC101927050	.
s7	chr2	110070071	C	A	upstream	MIR4267	.
s7	chr2	131218780	C	T	exonic	POTEE	synonymous_SNV
s7	chr2	131263974	C	T	exonic	POTEE	nonsynonymous_SNV
s7	chr2	131264056	G	T	exonic	POTEE	nonsynonymous_SNV
s7	chr2	232380134	G	A	intronic	ALPP	.
s7	chr2	232381539	A	G	exonic	ALPP	nonsynonymous_SNV
s7	chr2	236014717	-	T	intronic	AGAP1	.
s7	chr3	75665744	G	A	exonic	FRG2C	synonymous_SNV
s7	chr4	9243843	C	T	upstream	USP17L17	.
s7	chr4	9248548	G	A	upstream	USP17L11_USP17L18	.
s7	chr4	9248616	T	A	upstream	USP17L11_USP17L18	.
s7	chr4	9368818	T	C	ncRNA_exonic	USP17L6P	.
s7	chr5	34192589	A	G	intergenic	C1QTNF3-AMACR_NONE	.
s7	chr5	34193423	A	G	intergenic	C1QTNF3-AMACR_NONE	.
s7	chr5	141123606	T	C	exonic	PCDHB4	synonymous_SNV
s7	chr5	141123609	T	C	exonic	PCDHB4	synonymous_SNV
s7	chr5	141201296	A	G	exonic	PCDHB11	nonsynonymous_SNV
s7	chr5	179652459	C	T	intergenic	C5orf60_LOC105377763	.
s7	chr5	179652523	A	C	intergenic	C5orf60_LOC105377763	.
s7	chr7	5958978	G	A	intronic	RSPH10B_RSPH10B2	.
s7	chr7	73226001	T	C	ncRNA_intronic	NCF1B	.
s7	chr7	100959304	T	C	exonic	MUC3A	nonsynonymous_SNV
s7	chr7	100963346	T	C	intronic	MUC3A	.
s7	chr7	100963374	C	T	intronic	MUC3A	.
s7	chr7	100995392	G	T	exonic	MUC12	nonsynonymous_SNV
s7	chr7	100998641	C	G	exonic	MUC12	nonsynonymous_SNV
s7	chr7	101000011	G	A	exonic	MUC12	nonsynonymous_SNV
s7	chr7	101000063	G	T	exonic	MUC12	nonsynonymous_SNV
s7	chr7	101000457	G	A	exonic	MUC12	synonymous_SNV
s7	chr7	101000612	G	A	exonic	MUC12	nonsynonymous_SNV
s7	chr7	101000614	G	A	exonic	MUC12	nonsynonymous_SNV
s7	chr7	101000626	A	G	exonic	MUC12	nonsynonymous_SNV
s7	chr7	101000633	C	T	exonic	MUC12	nonsynonymous_SNV

s7	chr7	101000638	A	G	exonic	MUC12	nonsynonymous_SNV
s7	chr7	102639154	G	T	exonic	UPK3BL1	synonymous_SNV
s7	chr8	85655689	G	T	upstream	REXO1L2P	.
s7	chr8	85655705	T	G	upstream	REXO1L2P	.
s7	chr8	85655746	G	C	upstream	REXO1L2P	.
s7	chr8	85655791	G	A	upstream	REXO1L2P	.
s7	chr8	100709528	A	C	exonic	PABPC1	synonymous_SNV
s7	chr9	34834503	G	A	ncRNA_exonic	FAM205BP	.
s7	chr9	96938627	C	T	exonic	NUTM2G	synonymous_SNV
s7	chr9	114323755	C	T	exonic	ORM1	synonymous_SNV
s7	chr9	122724407	T	C	exonic	OR1L4	nonsynonymous_SNV
s7	chr9	122724418	A	G	exonic	OR1L4	synonymous_SNV
s7	chr9	128153053	CA	-	intronic	LCN2	.
s7	chr9	138175729	A	T	ncRNA_exonic	TUBBP5	.
s7	chr9	138175895	C	T	ncRNA_exonic	TUBBP5	.
s7	chr9	138177026	G	C	ncRNA_exonic	TUBBP5	.
s7	chr10	48010538	G	A	ncRNA_exonic	AGAP12P	.
s7	chr10	49933321	C	T	exonic	PARG	nonsynonymous_SNV
s7	chr10	73675610	C	T	exonic	AGAP5	synonymous_SNV
s7	chr10	125896583	C	T	UTR5	FANK1	.
s7	chr11	4587032	A	C	exonic	OR52I2	nonsynonymous_SNV
s7	chr11	18247969	T	C	exonic	SAA2_SAA2-SAA4	nonsynonymous_SNV
s7	chr11	71538412	T	C	exonic	KRTAP5-8	synonymous_SNV
s7	chr12	9425638	T	A	ncRNA_intronic	DDX12P	.
s7	chr12	9429017	T	C	ncRNA_intronic	DDX12P	.
s7	chr12	31091731	C	T	exonic	DDX11	nonsynonymous_SNV
s7	chr12	31091769	C	T	exonic	DDX11	synonymous_SNV
s7	chr12	31091884	C	-	intronic	DDX11	.
s7	chr12	40487270	A	G	exonic	MUC19	unknown
s7	chr12	40487284	G	T	exonic	MUC19	unknown
s7	chr12	40487326	C	A	exonic	MUC19	unknown
s7	chr12	40490955	C	G	exonic	MUC19	unknown
s7	chr12	50352189	A	G	exonic	FAM186A	nonsynonymous_SNV
s7	chr12	52305249	G	A	exonic	KRT86	nonsynonymous_SNV
s7	chr13	18178024	A	C	intergenic	NONE_FAM230C	.
s7	chr14	19713112	G	A	exonic	OR11H2	nonsynonymous_SNV
s7	chr14	19713115	T	C	exonic	OR11H2	nonsynonymous_SNV
s7	chr14	21634306	C	T	exonic	OR10G2	synonymous_SNV
s7	chr15	74070868	G	A	UTR3	GOLGA6A	.
s7	chr15	101755290	A	G	intergenic	LOC100128108_OR4F6	.
s7	chr15	101760080	G	A	intergenic	LOC100128108_OR4F6	.
s7	chr15	101761314	G	A	intergenic	LOC100128108_OR4F6	.

s7	chr16	12203522	C	T	intronic	SNX29	.
s7	chr16	28723438	G	A	intronic	EIF3C_EIF3CL	.
s7	chr16	33741835	C	A	intergenic	LOC390705_ENPP7P13	.
s7	chr16	70131836	C	T	intronic	PDPR	.
s7	chr16	74391380	T	C	intronic	NPIPB15	.
s7	chr16	74409592	C	T	exonic	CLEC18B	nonsynonymous_SNV
s7	chr16	74409770	T	C	intronic	CLEC18B	.
s7	chr16	74418062	C	T	exonic	CLEC18B	synonymous_SNV
s7	chr16	74418072	G	C	exonic	CLEC18B	nonsynonymous_SNV
s7	chr17	15738095	T	C	intronic	TBC1D26	.
s7	chr17	15738101	G	C	intronic	TBC1D26	.
s7	chr17	18492817	T	C	exonic	LGALS9C	synonymous_SNV
s7	chr17	20589700	C	A	intergenic	CDRT15L2_LINC02088	.
s7	chr18	48210	G	A	intergenic	LOC102723376_ROCK1P1	.
s7	chr18	48213	G	A	intergenic	LOC102723376_ROCK1P1	.
s7	chr18	46969231	C	A	intronic	KATNAL2	.
s7	chr19	4511211	C	T	exonic	PLIN4	nonsynonymous_SNV
s7	chr19	22665021	A	G	exonic	ZNF492	nonsynonymous_SNV
s7	chr19	39877736	A	C	exonic	FCGBP	synonymous_SNV
s7	chr19	39877838	G	A	exonic	FCGBP	synonymous_SNV
s7	chr19	39894352	C	G	exonic	FCGBP	synonymous_SNV
s7	chr19	49958551	G	T	exonic	SIGLEC11	synonymous_SNV
s7	chr19	49969964	T	C	exonic	SIGLEC16	nonsynonymous_SNV
s7	chr20	29079212	A	G	intergenic	FRG1CP_FRG1DP	.
s7	chr20	29415735	C	A	ncRNA_exonic	FRG2EP	.
s7	chr20	29415770	T	C	ncRNA_intronic	FRG2EP	.
s7	chr20	29415781	T	G	ncRNA_intronic	FRG2EP	.
s7	chr20	29415796	A	C	ncRNA_intronic	FRG2EP	.
s7	chr20	29415836	G	A	ncRNA_intronic	FRG2EP	.
s7	chr20	29415864	C	T	ncRNA_intronic	FRG2EP	.
s7	chr21	8208391	-	G	ncRNA_exonic	RNA45SN2	.
s7	chr21	8208471	G	C	ncRNA_exonic	RNA45SN2	.
s7	chr21	8208487	-	GGT	ncRNA_exonic	MIR3648-1_MIR3648-2_RNA45SN2	.
s7	chr21	8214799	-	C	ncRNA_exonic	RNA28SN2_RNA45SN2	.
s7	chr21	9068586	A	G	ncRNA_exonic	TEKT4P2	.
s7	chr21	9068589	G	A	ncRNA_exonic	TEKT4P2	.
s7	chr21	10462834	A	G	splicing	BAGE2_BAGE3_BAGE4_BAGE5	.
s7	chr21	10462836	C	T	exonic	BAGE2_BAGE3	stopgain
s7	chr21	10462861	G	A	exonic	BAGE2_BAGE3	nonsynonymous_SNV
s7	chr21	10462865	G	A	exonic	BAGE2_BAGE3	synonymous_SNV
s7	chr21	10462873	G	A	exonic	BAGE2_BAGE3	nonsynonymous_SNV
s7	chr21	10463002	C	T	UTR3	BAGE2_BAGE3_BAGE4_BAGE5	.

s7	chr21	10473490	A	G	UTR3	BAGE2_BAGE3_BAGE4_BAGE5	.
s7	chr21	10473548	C	A	UTR3	BAGE2_BAGE3_BAGE4_BAGE5	.
s7	chr21	44573789	A	G	exonic	KRTAP10-4	nonsynonymous_SNV
s7	chr21	44574137	C	T	exonic	KRTAP10-4	nonsynonymous_SNV
s7	chr21	44592464	G	A	exonic	KRTAP10-6	synonymous_SNV
s7	chr21	44592470	G	A	exonic	KRTAP10-6	synonymous_SNV
s7	chr22	15711039	-	TC	intronic	POTEH	.
s7	chr22	18537293	G	A	ncRNA_exonic	PI4KAP1	.
s7	chr22	50530398	AG	-	upstream_downstream	TYMP_ODF3B	.
s7	chrX	51333049	G	A	exonic	NUDT10	synonymous_SNV
s7	chr1_KI270711v1_random	7809	G	T	intergenic	NONE_NONE	.
s7	chr1_KI270711v1_random	7945	T	A	intergenic	NONE_NONE	.
s7	chr1_KI270711v1_random	8083	G	A	intergenic	NONE_NONE	.
s7	chr1_KI270711v1_random	8545	C	G	intergenic	NONE_NONE	.
s7	chr1_KI270711v1_random	8565	T	C	intergenic	NONE_NONE	.
s7	chr1_KI270711v1_random	8579	T	A	intergenic	NONE_NONE	.
s7	chr1_KI270711v1_random	9142	C	A	intergenic	NONE_NONE	.
s7	chr1_KI270711v1_random	9143	C	T	intergenic	NONE_NONE	.
s7	chr1_KI270711v1_random	9777	A	G	intergenic	NONE_NONE	.
s7	chr1_KI270711v1_random	9782	C	T	intergenic	NONE_NONE	.
s7	chr1_KI270711v1_random	9802	G	A	intergenic	NONE_NONE	.
s7	chr1_KI270711v1_random	23064	A	G	intergenic	NONE_NONE	.
s7	chr1_KI270711v1_random	23085	A	C	intergenic	NONE_NONE	.
s7	chr1_KI270713v1_random	3209	ACAGC	-	upstream	LOC102724562	.
s7	chr1_KI270713v1_random	3236	T	C	upstream	LOC102724562	.
s7	chr1_KI270713v1_random	3816	A	G	upstream	LOC102724562	.
s7	chr1_KI270713v1_random	6131	C	T	ncRNA_intronic	LOC102724562	.
s7	chr1_KI270713v1_random	33258	C	A	ncRNA_exonic	LOC440570	.
s7	chr9_KI270719v1_random	164929	G	A	intergenic	NONE_NONE	.
s7	chr9_KI270720v1_random	5143	C	T	intergenic	NONE_NONE	.
s7	chr9_KI270720v1_random	26078	G	A	intergenic	NONE_NONE	.
s7	chr9_KI270720v1_random	26184	T	C	intergenic	NONE_NONE	.
s7	chr14_GL000194v1_random	53745	C	T	ncRNA_exonic	MAFIP	.
s7	chr22_KI270733v1_random	169995	C	T	upstream	MIR3687-1_MIR3687-2	.
s7	chrUn_KI270744v1	80564	G	A	intergenic	NONE_NONE	.
s7	chrUn_KI270746v1	35656	A	T	intergenic	NONE_NONE	.
s7	chrUn_KI270746v1	35670	C	T	intergenic	NONE_NONE	.
s7	chrUn_KI270746v1	35985	T	A	intergenic	NONE_NONE	.
s7	chrUn_KI270746v1	36144	A	C	intergenic	NONE_NONE	.
s7	chrUn_GL000218v1	40556	G	A	ncRNA_intronic	LOC100233156	.
s7	chrUn_GL000218v1	40716	C	T	ncRNA_exonic	LOC100233156	.
s7	chr11_KI270902v1_alt	81668	A	G	intronic	MUC6	.

Sample	Chr	Start	Ref	Alt	Func.refGene	Gene.refGene	ExonicFunc.refGene
s8	chr1	1486536	T	C	intronic	ATAD3B	.
s8	chr1	12893329	C	G	exonic	PRAMEF10	nonsynonymous_SNV
s8	chr1	13319482	A	C	exonic	PRAMEF15	nonsynonymous_SNV
s8	chr1	16536138	C	T	upstream	LINC01783	.
s8	chr1	16565625	G	T	intronic	NBPF1	.
s8	chr1	16577252	T	C	intronic	NBPF1	.
s8	chr1	16577259	C	T	intronic	NBPF1	.
s8	chr1	16581539	C	G	intronic	NBPF1	.
s8	chr1	16645595	G	A	ncRNA_exonic	MST1P2	.
s8	chr1	16648250	G	A	ncRNA_exonic	MST1P2	.
s8	chr1	16648263	G	A	ncRNA_exonic	MST1P2	.
s8	chr1	16648285	C	T	ncRNA_exonic	MST1P2	.
s8	chr1	16648291	G	C	ncRNA_exonic	MST1P2	.
s8	chr1	16648783	-	CC	ncRNA_exonic	MST1P2	.
s8	chr1	16649698	C	T	ncRNA_exonic	MST1P2	.
s8	chr1	16650155	G	A	ncRNA_exonic	MST1P2	.
s8	chr1	16760804	T	C	exonic	MST1L	synonymous_SNV
s8	chr1	16760810	G	C	exonic	MST1L	synonymous_SNV
s8	chr1	16764407	-	T	intronic	MST1L	.
s8	chr1	22002726	C	T	intronic	CELA3A	.
s8	chr1	143498999	A	T	intergenic	LOC645166_RNVU1-17	.
s8	chr1	146989666	T	G	exonic	NBPF12	nonsynonymous_SNV
s8	chr1	146989687	A	G	exonic	NBPF12	nonsynonymous_SNV
s8	chr1	146989731	G	C	exonic	NBPF12	nonsynonymous_SNV
s8	chr1	149812045	G	A	exonic	HIST2H2BF	synonymous_SNV
s8	chr1	152214232	C	T	exonic	HRNR	nonsynonymous_SNV
s8	chr1	152215143	A	G	exonic	HRNR	synonymous_SNV
s8	chr1	152215642	C	T	exonic	HRNR	nonsynonymous_SNV
s8	chr1	152215843	C	T	exonic	HRNR	nonsynonymous_SNV
s8	chr1	152216371	A	G	exonic	HRNR	nonsynonymous_SNV
s8	chr1	154322023	G	A	exonic	AQP10	nonsynonymous_SNV
s8	chr1	154607873	AC	-	intronic	ADAR	.
s8	chr1	201209877	A	G	exonic	IGFN1	nonsynonymous_SNV
s8	chr2	91443526	G	C	intergenic	LOC101927050_LOC654342	.
s8	chr2	91443601	C	A	intergenic	LOC101927050_LOC654342	.
s8	chr2	91443607	C	A	intergenic	LOC101927050_LOC654342	.
s8	chr2	91443617	G	C	intergenic	LOC101927050_LOC654342	.
s8	chr2	106425156	A	C	exonic	RGPD3	nonsynonymous_SNV
s8	chr2	106425182	C	T	exonic	RGPD3	nonsynonymous_SNV
s8	chr2	110070071	C	A	upstream	MIR4267	.
s8	chr2	112390163	C	T	exonic	RGPD5_RGPD8	nonsynonymous_SNV

s8	chr2	128268771	A	G	exonic	HS6ST1	synonymous_SNV
s8	chr2	128318365	T	A	exonic	HS6ST1	stopgain
s8	chr2	128318387	T	C	exonic	HS6ST1	synonymous_SNV
s8	chr2	130075300	G	A	exonic	POTEF	synonymous_SNV
s8	chr2	131218780	C	T	exonic	POTEE	synonymous_SNV
s8	chr2	131480159	C	T	exonic	TUBA3D	nonsynonymous_SNV
s8	chr2	131491116	G	A	intronic	MZT2A	.
s8	chr2	232380134	G	A	intronic	ALPP	.
s8	chr2	232381443	C	T	intronic	ALPP	.
s8	chr3	75665231	G	T	intronic	FRG2C	.
s8	chr3	75665237	G	T	intronic	FRG2C	.
s8	chr3	75665744	G	A	exonic	FRG2C	synonymous_SNV
s8	chr3	75669267	T	G	intergenic	FRG2C_LINC00960	.
s8	chr3	75669271	A	C	intergenic	FRG2C_LINC00960	.
s8	chr4	9243843	C	T	upstream	USP17L17	.
s8	chr4	9248548	G	A	upstream	USP17L11_USP17L18	.
s8	chr4	9248616	T	A	upstream	USP17L11_USP17L18	.
s8	chr4	9268602	G	A	exonic	USP17L20_USP17L22	stopgain
s8	chr5	34191075	T	A	intergenic	C1QTNF3-AMACR_NONE	.
s8	chr5	34192589	A	G	intergenic	C1QTNF3-AMACR_NONE	.
s8	chr5	34193423	A	G	intergenic	C1QTNF3-AMACR_NONE	.
s8	chr5	141123606	T	C	exonic	PCDHB4	synonymous_SNV
s8	chr5	141123609	T	C	exonic	PCDHB4	synonymous_SNV
s8	chr5	141201296	A	G	exonic	PCDHB11	nonsynonymous_SNV
s8	chr5	170080127	TG	-	intronic	DOCK2	.
s8	chr5	177672139	T	A	ncRNA_exonic	LOC202181	.
s8	chr6	27146790	T	C	exonic	HIST1H2BK	synonymous_SNV
s8	chr6	167179083	G	A	exonic	TCP10L2	nonsynonymous_SNV
s8	chr7	2659891	CT	-	intronic	TTYH3	.
s8	chr7	5958978	G	A	intronic	RSPH10B_RSPH10B2	.
s8	chr7	38358446	T	C	ncRNA_intronic	TRG-AS1	.
s8	chr7	43986652	T	C	ncRNA_exonic	POLR2J4	.
s8	chr7	73226001	T	C	ncRNA_intronic	NCF1B	.
s8	chr7	73244222	A	G	intergenic	NCF1B_NSUN5	.
s8	chr7	100995392	G	T	exonic	MUC12	nonsynonymous_SNV
s8	chr7	100998641	C	G	exonic	MUC12	nonsynonymous_SNV
s8	chr7	101000011	G	A	exonic	MUC12	nonsynonymous_SNV
s8	chr7	101000063	G	T	exonic	MUC12	nonsynonymous_SNV
s8	chr7	101000457	G	A	exonic	MUC12	synonymous_SNV
s8	chr7	101000595	C	T	exonic	MUC12	synonymous_SNV
s8	chr7	101000597	A	C	exonic	MUC12	nonsynonymous_SNV
s8	chr7	101000612	G	A	exonic	MUC12	nonsynonymous_SNV

s8	chr7	101000614	G	A	exonic	MUC12	nonsynonymous_SNV
s8	chr7	101000626	A	G	exonic	MUC12	nonsynonymous_SNV
s8	chr7	152265049	G	T	exonic	KMT2C	stopgain
s8	chr7	152265083	C	A	exonic	KMT2C	nonsynonymous_SNV
s8	chr8	85655705	T	G	upstream	REXO1L2P	.
s8	chr8	85655746	G	C	upstream	REXO1L2P	.
s8	chr8	85655791	G	A	upstream	REXO1L2P	.
s8	chr8	142877834	T	C	intronic	CYP11B1	.
s8	chr9	34834503	G	A	ncRNA_exonic	FAM205BP	.
s8	chr9	62843444	C	T	ncRNA_intronic	PTGER4P2-CDK2AP2P2	.
s8	chr9	62860904	G	A	ncRNA_intronic	LOC403323	.
s8	chr9	65650246	T	C	intergenic	FOXD4L5_CBWD5	.
s8	chr9	65737280	G	A	exonic	FOXD4L4	synonymous_SNV
s8	chr9	96938627	C	T	exonic	NUTM2G	synonymous_SNV
s8	chr9	114323755	C	T	exonic	ORM1	synonymous_SNV
s8	chr9	122724407	T	C	exonic	OR1L4	nonsynonymous_SNV
s8	chr9	122724418	A	G	exonic	OR1L4	synonymous_SNV
s8	chr9	128153053	CA	-	intronic	LCN2	.
s8	chr9	138175895	C	T	ncRNA_exonic	TUBBP5	.
s8	chr10	73675610	C	T	exonic	AGAP5	synonymous_SNV
s8	chr10	79559428	G	A	exonic	SFTPA2	nonsynonymous_SNV
s8	chr10	79611881	T	C	exonic	SFTPA1	nonsynonymous_SNV
s8	chr10	80233739	C	A	intergenic	LINC00857_LOC100130698	.
s8	chr10	133625456	C	-	exonic	FRG2B	frameshift_deletion
s8	chr11	1244642	G	A	exonic	MUC5B	nonsynonymous_SNV
s8	chr11	4587032	A	C	exonic	OR52I2	nonsynonymous_SNV
s8	chr11	18247969	T	C	exonic	SAA2_SAA2-SAA4	nonsynonymous_SNV
s8	chr11	124016010	G	A	exonic	OR10G4	nonsynonymous_SNV
s8	chr11	124023021	G	C	exonic	OR10G9	nonsynonymous_SNV
s8	chr11	124023022	A	G	exonic	OR10G9	nonsynonymous_SNV
s8	chr11	124023084	A	C	exonic	OR10G9	synonymous_SNV
s8	chr12	9421465	G	-	ncRNA_exonic	DDX12P	.
s8	chr12	9425638	T	A	ncRNA_intronic	DDX12P	.
s8	chr12	9429017	T	C	ncRNA_intronic	DDX12P	.
s8	chr12	31091731	C	T	exonic	DDX11	nonsynonymous_SNV
s8	chr12	31091769	C	T	exonic	DDX11	synonymous_SNV
s8	chr12	31091884	C	-	intronic	DDX11	.
s8	chr12	52305249	G	A	exonic	KRT86	nonsynonymous_SNV
s8	chr12	52305260	C	G	exonic	KRT86	synonymous_SNV
s8	chr13	18177976	A	T	intergenic	NONE_FAM230C	.
s8	chr13	18177985	-	A	intergenic	NONE_FAM230C	.
s8	chr14	18601543	C	A	exonic	OR11H12	nonsynonymous_SNV

s8	chr14	18999460	A	G	UTR3	POTEM	.
s8	chr14	19402323	G	A	downstream	POTEG	.
s8	chr14	19488293	G	T	intergenic	POTEG_OR11H2	.
s8	chr15	20261847	A	G	intergenic	NONE_CHEK2P2	.
s8	chr15	101753899	A	G	intergenic	LOC100128108_OR4F6	.
s8	chr15	101760080	G	A	intergenic	LOC100128108_OR4F6	.
s8	chr15	101761203	T	C	intergenic	LOC100128108_OR4F6	.
s8	chr16	18585973	C	A	ncRNA_intronic	ABCC6P1	.
s8	chr16	28723438	G	A	intronic	EIF3C_EIF3CL	.
s8	chr16	33741748	G	A	intergenic	LOC390705_ENPP7P13	.
s8	chr16	33741835	C	A	intergenic	LOC390705_ENPP7P13	.
s8	chr16	70131836	C	T	intronic	PDPR	.
s8	chr16	70177476	C	T	exonic	CLEC18C	nonsynonymous_SNV
s8	chr16	74409592	C	T	exonic	CLEC18B	nonsynonymous_SNV
s8	chr16	74409770	T	C	intronic	CLEC18B	.
s8	chr16	90095783	G	A	intergenic	PRDM7_FAM157C	.
s8	chr17	15738226	G	C	intronic	TBC1D26	.
s8	chr17	18492905	-	AG	intronic	LGALS9C	.
s8	chr17	18778850	T	C	exonic	FBXW10	nonsynonymous_SNV
s8	chr17	20589700	C	A	intergenic	CDRT15L2_LINC02088	.
s8	chr17	21415458	G	A	exonic	KCNJ12	nonsynonymous_SNV
s8	chr17	21415461	G	A	exonic	KCNJ12	nonsynonymous_SNV
s8	chr17	21415585	G	C	exonic	KCNJ12	synonymous_SNV
s8	chr17	21415960	C	T	exonic	KCNJ12	synonymous_SNV
s8	chr17	21703029	C	G	exonic	KCNJ18	synonymous_SNV
s8	chr17	21703050	G	A	exonic	KCNJ18	synonymous_SNV
s8	chr17	21703211	C	A	exonic	KCNJ18	nonsynonymous_SNV
s8	chr17	38297226	T	G	exonic	MRPL45	nonsynonymous_SNV
s8	chr18	46969231	C	A	intronic	KATNAL2	.
s8	chr19	9980619	CA	-	intronic	COL5A3	.
s8	chr19	35011449	CT	-	intronic	GRAMD1A	.
s8	chr19	39877736	A	C	exonic	FCGBP	synonymous_SNV
s8	chr19	39894438	T	C	exonic	FCGBP	nonsynonymous_SNV
s8	chr19	40850019	T	C	intronic	CYP2A6	.
s8	chr19	43355655	T	C	intronic	CD177	.
s8	chr19	49044175	A	G	UTR5	CGB5	.
s8	chr19	49044189	G	C	UTR5	CGB5	.
s8	chr19	49958551	G	T	exonic	SIGLEC11	synonymous_SNV
s8	chr19	49969964	T	C	exonic	SIGLEC16	nonsynonymous_SNV
s8	chr19	53375790	T	C	exonic	ZNF525	synonymous_SNV
s8	chr19	53666561	G	A	upstream	MIR512-1_MIR512-2	.
s8	chr20	29079212	A	G	intergenic	FRG1CP_FRG1DP	.

s8	chr20	29415770	T	C	ncRNA_intronic	FRG2EP	.
s8	chr20	29415781	T	G	ncRNA_intronic	FRG2EP	.
s8	chr20	29415796	A	C	ncRNA_intronic	FRG2EP	.
s8	chr20	30388480	G	A	ncRNA_exonic	FRG1BP	.
s8	chr20	30815433	C	T	intergenic	FRG1BP_DEFB115	.
s8	chr21	9068586	A	G	ncRNA_exonic	TEKT4P2	.
s8	chr21	9068589	G	A	ncRNA_exonic	TEKT4P2	.
s8	chr21	10397827	-	T	intergenic	LINC01667_BAGE	.
s8	chr21	10462834	A	G	splicing	BAGE2_BAGE3_BAGE4_BAGE5	.
s8	chr21	10462836	C	T	exonic	BAGE2_BAGE3	stopgain
s8	chr21	10462861	G	A	exonic	BAGE2_BAGE3	nonsynonymous_SNV
s8	chr21	10462865	G	A	exonic	BAGE2_BAGE3	synonymous_SNV
s8	chr21	10462873	G	A	exonic	BAGE2_BAGE3	nonsynonymous_SNV
s8	chr21	10462962	G	T	UTR3	BAGE2_BAGE3_BAGE4_BAGE5	.
s8	chr21	10473245	G	A	UTR3	BAGE2_BAGE3_BAGE4_BAGE5	.
s8	chr21	10473490	A	G	UTR3	BAGE2_BAGE3_BAGE4_BAGE5	.
s8	chr21	44551226	G	A	exonic	KRTAP10-2	nonsynonymous_SNV
s8	chr21	44573789	A	G	exonic	KRTAP10-4	nonsynonymous_SNV
s8	chr21	44592464	G	A	exonic	KRTAP10-6	synonymous_SNV
s8	chr21	44592470	G	A	exonic	KRTAP10-6	synonymous_SNV
s8	chr22	10742085	C	G	intergenic	NONE_LOC102723780	.
s8	chr22	10752820	C	T	intergenic	NONE_LOC102723780	.
s8	chr22	18535978	A	T	ncRNA_intronic	PI4KAP1	.
s8	chr22	18536633	T	C	ncRNA_intronic	PI4KAP1	.
s8	chr22	50530398	AG	-	upstream_downstream	TYMP_ODF3B	.
s8	chr1_KI270711v1_random	7945	T	A	intergenic	NONE_NONE	.
s8	chr1_KI270711v1_random	8083	G	A	intergenic	NONE_NONE	.
s8	chr1_KI270711v1_random	8545	C	G	intergenic	NONE_NONE	.
s8	chr1_KI270711v1_random	8565	T	C	intergenic	NONE_NONE	.
s8	chr1_KI270711v1_random	8579	T	A	intergenic	NONE_NONE	.
s8	chr1_KI270711v1_random	9142	C	A	intergenic	NONE_NONE	.
s8	chr1_KI270711v1_random	9143	C	T	intergenic	NONE_NONE	.
s8	chr1_KI270711v1_random	9782	C	T	intergenic	NONE_NONE	.
s8	chr1_KI270711v1_random	9784	G	A	intergenic	NONE_NONE	.
s8	chr1_KI270711v1_random	9802	G	A	intergenic	NONE_NONE	.
s8	chr1_KI270711v1_random	23064	A	G	intergenic	NONE_NONE	.
s8	chr1_KI270711v1_random	23085	A	C	intergenic	NONE_NONE	.
s8	chr1_KI270711v1_random	23134	G	A	intergenic	NONE_NONE	.
s8	chr1_KI270713v1_random	3164	G	A	intergenic	NONE_LOC102724562	.
s8	chr1_KI270713v1_random	3209	ACAGC	-	upstream	LOC102724562	.
s8	chr1_KI270713v1_random	3236	T	C	upstream	LOC102724562	.
s8	chr1_KI270713v1_random	3495	A	G	upstream	LOC102724562	.

s8	chr1_KI270713v1_random	3816	A	G	upstream	LOC102724562	.
s8	chr1_KI270713v1_random	32494	C	T	ncRNA_exonic	LOC440570	.
s8	chr1_KI270713v1_random	32593	G	A	ncRNA_exonic	LOC440570	.
s8	chr9_KI270719v1_random	164929	G	A	intergenic	NONE_NONE	.
s8	chr9_KI270720v1_random	5136	C	T	intergenic	NONE_NONE	.
s8	chr9_KI270720v1_random	5143	C	T	intergenic	NONE_NONE	.
s8	chr9_KI270720v1_random	5153	T	C	intergenic	NONE_NONE	.
s8	chr9_KI270720v1_random	5162	G	C	intergenic	NONE_NONE	.
s8	chr9_KI270720v1_random	26184	T	C	intergenic	NONE_NONE	.
s8	chr22_KI270733v1_random	169995	C	T	upstream	MIR3687-1_MIR3687-2	.
s8	chrUn_GL000220v1	142182	G	A	intergenic	LOC100507412_MIR6724-4	.
s8	chrUn_KI270744v1	80564	G	A	intergenic	NONE_NONE	.
s8	chrUn_KI270746v1	35656	A	T	intergenic	NONE_NONE	.
s8	chrUn_KI270746v1	35670	C	T	intergenic	NONE_NONE	.
s8	chrUn_KI270746v1	35985	T	A	intergenic	NONE_NONE	.
s8	chrUn_KI270746v1	36144	A	C	intergenic	NONE_NONE	.
s8	chrUn_GL000218v1	40716	C	T	ncRNA_exonic	LOC100233156	.
s8	chr3_KI270779v1_alt	163560	T	G	exonic	MUC4	nonsynonymous_SNV
s8	chr3_KI270779v1_alt	163564	T	C	exonic	MUC4	nonsynonymous_SNV
s8	chr11_KI270902v1_alt	81918	G	T	intronic	MUC6	.
s8	chr11_KI270902v1_alt	81925	G	T	intronic	MUC6	.
s8	chr11_KI270902v1_alt	81936	A	C	intronic	MUC6	.
s8	chr11_KI270902v1_alt	81984	G	A	intronic	MUC6	.
Sample	Chr	Start	Ref	Alt	Func.refGene	Gene.refGene	ExonicFunc.refGene
s9	chr1	1486536	T	C	intronic	ATAD3B	.
s9	chr1	12827315	C	T	exonic	PRAMEF11	nonsynonymous_SNV
s9	chr1	13319482	A	C	exonic	PRAMEF15	nonsynonymous_SNV
s9	chr1	16536138	C	T	upstream	LINC01783	.
s9	chr1	16565625	G	T	intronic	NBPF1	.
s9	chr1	16577252	T	C	intronic	NBPF1	.
s9	chr1	16577259	C	T	intronic	NBPF1	.
s9	chr1	16581396	T	C	intronic	NBPF1	.
s9	chr1	16581539	C	G	intronic	NBPF1	.
s9	chr1	16583468	G	A	intronic	NBPF1	.
s9	chr1	16589116	T	C	intronic	NBPF1	.
s9	chr1	16645595	G	A	ncRNA_exonic	MST1P2	.
s9	chr1	16648250	G	A	ncRNA_exonic	MST1P2	.
s9	chr1	16648263	G	A	ncRNA_exonic	MST1P2	.
s9	chr1	16648285	C	T	ncRNA_exonic	MST1P2	.
s9	chr1	16648291	G	C	ncRNA_exonic	MST1P2	.
s9	chr1	16649698	C	T	ncRNA_exonic	MST1P2	.
s9	chr1	16650155	G	A	ncRNA_exonic	MST1P2	.

s9	chr1	16757393	C	T	exonic	MST1L	nonsynonymous_SNV
s9	chr1	16760804	T	C	exonic	MST1L	synonymous_SNV
s9	chr1	16760810	G	C	exonic	MST1L	synonymous_SNV
s9	chr1	22002726	C	T	intronic	CELA3A	.
s9	chr1	47144445	C	T	exonic	CYP4A22	nonsynonymous_SNV
s9	chr1	146989666	T	G	exonic	NBPF12	nonsynonymous_SNV
s9	chr1	146989687	A	G	exonic	NBPF12	nonsynonymous_SNV
s9	chr1	146989731	G	C	exonic	NBPF12	nonsynonymous_SNV
s9	chr1	146989882	G	A	intronic	NBPF12	.
s9	chr1	149812045	G	A	exonic	HIST2H2BF	synonymous_SNV
s9	chr1	152110008	G	T	exonic	TCHH	nonsynonymous_SNV
s9	chr1	152155756	T	C	exonic	RPTN	nonsynonymous_SNV
s9	chr1	152214232	C	T	exonic	HRNR	nonsynonymous_SNV
s9	chr1	152214433	C	T	exonic	HRNR	nonsynonymous_SNV
s9	chr1	152215143	A	G	exonic	HRNR	synonymous_SNV
s9	chr1	152215642	C	T	exonic	HRNR	nonsynonymous_SNV
s9	chr1	152215843	C	T	exonic	HRNR	nonsynonymous_SNV
s9	chr1	152216371	A	G	exonic	HRNR	nonsynonymous_SNV
s9	chr1	154322023	G	A	exonic	AQP10	nonsynonymous_SNV
s9	chr1	154607873	AC	-	intronic	ADAR	.
s9	chr1	248061187	A	G	exonic	OR2L3	nonsynonymous_SNV
s9	chr1	248061267	G	T	exonic	OR2L3	nonsynonymous_SNV
s9	chr1	248061293	C	T	exonic	OR2L3	synonymous_SNV
s9	chr2	90022153	G	A	intergenic	LOC101927050_LOC654342	.
s9	chr2	94199359	A	C	ncRNA_exonic	BMS1P14	.
s9	chr2	95938785	T	G	intronic	ANKRD36C	.
s9	chr2	95945265	A	T	intronic	ANKRD36C	.
s9	chr2	112390163	C	T	exonic	RGPD5_RGPD8	nonsynonymous_SNV
s9	chr2	113595705	G	T	ncRNA_exonic	WASH2P	.
s9	chr2	128318365	T	A	exonic	HS6ST1	stopgain
s9	chr2	130075300	G	A	exonic	POTEF	synonymous_SNV
s9	chr2	130656706	A	G	exonic	POTEJ	nonsynonymous_SNV
s9	chr2	131264056	G	T	exonic	POTEE	nonsynonymous_SNV
s9	chr3	75665231	G	T	intronic	FRG2C	.
s9	chr3	75665237	G	T	intronic	FRG2C	.
s9	chr3	75665744	G	A	exonic	FRG2C	synonymous_SNV
s9	chr4	9239150	C	G	intergenic	USP17L15_USP17L17	.
s9	chr4	9243843	C	T	upstream	USP17L17	.
s9	chr4	9248616	T	A	upstream	USP17L11_USP17L18	.
s9	chr4	9268602	G	A	exonic	USP17L20_USP17L22	stopgain
s9	chr5	34191075	T	A	intergenic	C1QTNF3-AMACR_NONE	.
s9	chr5	34192589	A	G	intergenic	C1QTNF3-AMACR_NONE	.

s9	chr5	34193423	A	G	intergenic	C1QTNF3-AMACR_NONE	.
s9	chr5	141123411	T	C	exonic	PCDHB4	synonymous_SNV
s9	chr5	141123606	T	C	exonic	PCDHB4	synonymous_SNV
s9	chr5	141123609	T	C	exonic	PCDHB4	synonymous_SNV
s9	chr5	141201296	A	G	exonic	PCDHB11	nonsynonymous_SNV
s9	chr5	177672139	T	A	ncRNA_exonic	LOC202181	.
s9	chr5	179652523	A	C	intergenic	C5orf60_LOC105377763	.
s9	chr6	27146790	T	C	exonic	HIST1H2BK	synonymous_SNV
s9	chr6	109440150	GT	-	intronic	PPIL6	.
s9	chr6	167179083	G	A	exonic	TCP10L2	nonsynonymous_SNV
s9	chr7	5958978	G	A	intronic	RSPH10B_RSPH10B2	.
s9	chr7	38358446	T	C	ncRNA_intronic	TRG-AS1	.
s9	chr7	100995392	G	T	exonic	MUC12	nonsynonymous_SNV
s9	chr7	100998641	C	G	exonic	MUC12	nonsynonymous_SNV
s9	chr7	100998937	G	A	exonic	MUC12	nonsynonymous_SNV
s9	chr7	100998948	T	G	exonic	MUC12	synonymous_SNV
s9	chr7	101000011	G	A	exonic	MUC12	nonsynonymous_SNV
s9	chr7	101000063	G	T	exonic	MUC12	nonsynonymous_SNV
s9	chr7	101000457	G	A	exonic	MUC12	synonymous_SNV
s9	chr7	101000612	G	A	exonic	MUC12	nonsynonymous_SNV
s9	chr7	101000614	G	A	exonic	MUC12	nonsynonymous_SNV
s9	chr7	101000626	A	G	exonic	MUC12	nonsynonymous_SNV
s9	chr7	101000633	C	T	exonic	MUC12	nonsynonymous_SNV
s9	chr7	101000638	A	G	exonic	MUC12	nonsynonymous_SNV
s9	chr7	102348400	TGGAACGGTATAACTGG	-	exonic	SPDYE6	nonframeshift_deletion
s9	chr7	102554519	G	A	exonic	SPDYE2_SPDYE2B	synonymous_SNV
s9	chr7	102639154	G	T	exonic	UPK3BL1	synonymous_SNV
s9	chr7	152265205	C	G	exonic	KMT2C	nonsynonymous_SNV
s9	chr8	85655689	G	T	upstream	REXO1L2P	.
s9	chr8	85655705	T	G	upstream	REXO1L2P	.
s9	chr8	85655746	G	C	upstream	REXO1L2P	.
s9	chr8	100709528	A	C	exonic	PABPC1	synonymous_SNV
s9	chr9	34834503	G	A	ncRNA_exonic	FAM205BP	.
s9	chr9	62801506	G	T	ncRNA_exonic	LINC01410	.
s9	chr9	62858238	G	A	ncRNA_intronic	LOC403323	.
s9	chr9	62860904	G	A	ncRNA_intronic	LOC403323	.
s9	chr9	65650207	G	A	intergenic	FOXD4L5_CBWD5	.
s9	chr9	65650246	T	C	intergenic	FOXD4L5_CBWD5	.
s9	chr9	96938627	C	T	exonic	NUTM2G	synonymous_SNV
s9	chr9	114323755	C	T	exonic	ORM1	synonymous_SNV
s9	chr9	122724407	T	C	exonic	OR1L4	nonsynonymous_SNV
s9	chr9	122724418	A	G	exonic	OR1L4	synonymous_SNV

s9	chr9	128153053	CA	-	intronic	LCN2	.
s9	chr9	137102312	G	C	intronic	MAN1B1	.
s9	chr9	138175729	A	T	ncRNA_exonic	TUBBP5	.
s9	chr9	138175895	C	T	ncRNA_exonic	TUBBP5	.
s9	chr10	9407586	T	C	intergenic	LOC101928272_LOC101928298	.
s9	chr10	73675610	C	T	exonic	AGAP5	synonymous_SNV
s9	chr10	79559428	G	A	exonic	SFTPA2	nonsynonymous_SNV
s9	chr10	79611798	T	A	intronic	SFTPA1	.
s9	chr10	133625456	C	-	exonic	FRG2B	frameshift_deletion
s9	chr10	133625511	T	C	exonic	FRG2B	nonsynonymous_SNV
s9	chr11	1187040	T	C	exonic	MUC5AC	synonymous_SNV
s9	chr11	1244642	G	A	exonic	MUC5B	nonsynonymous_SNV
s9	chr11	4587032	A	C	exonic	OR52I2	nonsynonymous_SNV
s9	chr11	18247969	T	C	exonic	SAA2_SAA2-SAA4	nonsynonymous_SNV
s9	chr11	64315749	G	C	exonic	ESRRA	nonsynonymous_SNV
s9	chr11	71527399	C	T	exonic	KRTAP5-7	synonymous_SNV
s9	chr12	9421465	G	-	ncRNA_exonic	DDX12P	.
s9	chr12	9425638	T	A	ncRNA_intronic	DDX12P	.
s9	chr12	9429017	T	C	ncRNA_intronic	DDX12P	.
s9	chr12	31091769	C	T	exonic	DDX11	synonymous_SNV
s9	chr12	31091884	C	-	intronic	DDX11	.
s9	chr12	40483243	T	G	exonic	MUC19	unknown
s9	chr12	40483247	A	G	exonic	MUC19	unknown
s9	chr13	18177976	A	T	intergenic	NONE_FAM230C	.
s9	chr13	18177985	-	A	intergenic	NONE_FAM230C	.
s9	chr13	24321255	A	G	exonic	C1QTNF9	synonymous_SNV
s9	chr14	19488293	G	T	intergenic	POTEG_OR11H2	.
s9	chr15	20261847	A	G	intergenic	NONE_CHEK2P2	.
s9	chr15	74071890	C	T	intronic	GOLGA6A	.
s9	chr15	74071891	A	G	intronic	GOLGA6A	.
s9	chr15	101752697	T	C	intergenic	LOC100128108_OR4F6	.
s9	chr15	101753899	A	G	intergenic	LOC100128108_OR4F6	.
s9	chr15	101755290	A	G	intergenic	LOC100128108_OR4F6	.
s9	chr15	101757294	G	A	intergenic	LOC100128108_OR4F6	.
s9	chr15	101760080	G	A	intergenic	LOC100128108_OR4F6	.
s9	chr15	101772004	A	G	intergenic	LOC100128108_OR4F6	.
s9	chr16	28723438	G	A	intronic	EIF3C_EIF3CL	.
s9	chr16	33741748	G	A	intergenic	LOC390705_ENPP7P13	.
s9	chr16	67195891	CAG	-	exonic	E2F4	nonframeshift_deletion
s9	chr16	69954480	C	T	exonic	CLEC18A	synonymous_SNV
s9	chr16	70120577	A	G	exonic	PDPR	nonsynonymous_SNV
s9	chr16	70131836	C	T	intronic	PDPR	.

s9	chr16	74391416	G	A	exonic	NPIPB15	nonsynonymous_SNV
s9	chr16	74409592	C	T	exonic	CLEC18B	nonsynonymous_SNV
s9	chr16	74409652	G	T	intronic	CLEC18B	.
s9	chr16	74409770	T	C	intronic	CLEC18B	.
s9	chr17	1470359	T	G	intronic	MYO1C	.
s9	chr17	15619445	A	T	exonic	CDRT1	nonsynonymous_SNV
s9	chr17	20589700	C	A	intergenic	CDRT15L2_LINC02088	.
s9	chr17	21415458	G	A	exonic	KCNJ12	nonsynonymous_SNV
s9	chr17	21415461	G	A	exonic	KCNJ12	nonsynonymous_SNV
s9	chr17	21415585	G	C	exonic	KCNJ12	synonymous_SNV
s9	chr17	21415960	C	T	exonic	KCNJ12	synonymous_SNV
s9	chr17	21703050	G	A	exonic	KCNJ18	synonymous_SNV
s9	chr18	46969231	C	A	intronic	KATNAL2	.
s9	chr19	9980619	CA	-	intronic	COL5A3	.
s9	chr19	22665021	A	G	exonic	ZNF492	nonsynonymous_SNV
s9	chr19	35011449	CT	-	intronic	GRAMD1A	.
s9	chr19	39877838	G	A	exonic	FCGBP	synonymous_SNV
s9	chr19	40850019	T	C	intronic	CYP2A6	.
s9	chr19	49044175	A	G	UTR5	CGB5	.
s9	chr19	49044189	G	C	UTR5	CGB5	.
s9	chr19	49958551	G	T	exonic	SIGLEC11	synonymous_SNV
s9	chr19	49959383	T	C	exonic	SIGLEC11	nonsynonymous_SNV
s9	chr19	49969964	T	C	exonic	SIGLEC16	nonsynonymous_SNV
s9	chr19	53375790	T	C	exonic	ZNF525	synonymous_SNV
s9	chr19	55772851	A	T	exonic	RFPL4AL1	nonsynonymous_SNV
s9	chr20	29079153	C	A	intergenic	FRG1CP_FRG1DP	.
s9	chr20	29079212	A	G	intergenic	FRG1CP_FRG1DP	.
s9	chr20	29415781	T	G	ncRNA_intronic	FRG2EP	.
s9	chr20	29415796	A	C	ncRNA_intronic	FRG2EP	.
s9	chr20	29415836	G	A	ncRNA_intronic	FRG2EP	.
s9	chr21	9068586	A	G	ncRNA_exonic	TEKT4P2	.
s9	chr21	9068589	G	A	ncRNA_exonic	TEKT4P2	.
s9	chr21	10397827	-	T	intergenic	LINC01667_BAGE	.
s9	chr21	10454230	A	T	exonic	BAGE2_BAGE3	synonymous_SNV
s9	chr21	10462834	A	G	splicing	BAGE2_BAGE3_BAGE4_BAGE5	.
s9	chr21	10462836	C	T	exonic	BAGE2_BAGE3	stopgain
s9	chr21	10462861	G	A	exonic	BAGE2_BAGE3	nonsynonymous_SNV
s9	chr21	10462865	G	A	exonic	BAGE2_BAGE3	synonymous_SNV
s9	chr21	10462873	G	A	exonic	BAGE2_BAGE3	nonsynonymous_SNV
s9	chr21	10463002	C	T	UTR3	BAGE2_BAGE3_BAGE4_BAGE5	.
s9	chr21	10473245	G	A	UTR3	BAGE2_BAGE3_BAGE4_BAGE5	.
s9	chr21	10473366	A	T	UTR3	BAGE2_BAGE3_BAGE4_BAGE5	.

s9	chr21	10473490	A	G	UTR3	BAGE2_BAGE3_BAGE4_BAGE5	.
s9	chr21	10473500	A	G	UTR3	BAGE2_BAGE3_BAGE4_BAGE5	.
s9	chr21	34670179	T	G	exonic	CLIC6	nonsynonymous_SNV
s9	chr22	10742085	C	G	intergenic	NONE_LOC102723780	.
s9	chr22	15690342	TCTGCTATGAAGACACT(	-	exonic	POTEH	nonframeshift_deletion
s9	chr22	18492273	G	A	ncRNA_exonic	FAM230A	.
s9	chr22	18535978	A	T	ncRNA_intronic	PI4KAP1	.
s9	chr22	18536633	T	C	ncRNA_intronic	PI4KAP1	.
s9	chr22	18608233	-	CAC	exonic	RIMBP3	nonframeshift_insertion
s9	chr22	18608251	G	A	exonic	RIMBP3	nonsynonymous_SNV
s9	chr22	21309195	TGGCGATGCCCTGGGC(	-	ncRNA_exonic	LOC100996335	.
s9	chr22	37724422	T	C	exonic	TRIOBP	synonymous_SNV
s9	chr22	50530398	AG	-	upstream_downstream	TYMP_ODF3B	.
s9	chr1_KI270711v1_random	7945	T	A	intergenic	NONE_NONE	.
s9	chr1_KI270711v1_random	8083	G	A	intergenic	NONE_NONE	.
s9	chr1_KI270711v1_random	8421	T	C	intergenic	NONE_NONE	.
s9	chr1_KI270711v1_random	8545	C	G	intergenic	NONE_NONE	.
s9	chr1_KI270711v1_random	8565	T	C	intergenic	NONE_NONE	.
s9	chr1_KI270711v1_random	8579	T	A	intergenic	NONE_NONE	.
s9	chr1_KI270711v1_random	9142	C	A	intergenic	NONE_NONE	.
s9	chr1_KI270711v1_random	9143	C	T	intergenic	NONE_NONE	.
s9	chr1_KI270711v1_random	9802	G	A	intergenic	NONE_NONE	.
s9	chr1_KI270711v1_random	23064	A	G	intergenic	NONE_NONE	.
s9	chr1_KI270711v1_random	23085	A	C	intergenic	NONE_NONE	.
s9	chr1_KI270711v1_random	26413	T	C	intergenic	NONE_NONE	.
s9	chr1_KI270713v1_random	3816	A	G	upstream	LOC102724562	.
s9	chr1_KI270713v1_random	4608	G	C	ncRNA_exonic	LOC102724562	.
s9	chr1_KI270713v1_random	32494	C	T	ncRNA_exonic	LOC440570	.
s9	chr9_KI270719v1_random	164929	G	A	intergenic	NONE_NONE	.
s9	chr9_KI270720v1_random	26078	G	A	intergenic	NONE_NONE	.
s9	chr9_KI270720v1_random	26184	T	C	intergenic	NONE_NONE	.
s9	chr14_GL000194v1_random	53745	C	T	ncRNA_exonic	MAFIP	.
s9	chr16_KI270728v1_random	1450885	A	G	intergenic	ENPP7P13_NONE	.
s9	chr22_KI270733v1_random	169995	C	T	upstream	MIR3687-1_MIR3687-2	.
s9	chrUn_KI270744v1	80564	G	A	intergenic	NONE_NONE	.
s9	chrUn_KI270746v1	35656	A	T	intergenic	NONE_NONE	.
s9	chrUn_KI270746v1	35670	C	T	intergenic	NONE_NONE	.
s9	chrUn_KI270746v1	35985	T	A	intergenic	NONE_NONE	.
s9	chrUn_KI270746v1	36144	A	C	intergenic	NONE_NONE	.
s9	chrUn_KI270746v1	40552	T	C	intergenic	NONE_NONE	.
s9	chrUn_GL000218v1	40556	G	A	ncRNA_intronic	LOC100233156	.
s9	chrUn_GL000218v1	40716	C	T	ncRNA_exonic	LOC100233156	.

s9	chr11_KI270902v1_alt	81874	G	A	intronic	MUC6	.
s9	chr11_KI270902v1_alt	81885	C	T	intronic	MUC6	.
s9	chr11_KI270902v1_alt	81892	C	G	intronic	MUC6	.
s9	chr11_KI270902v1_alt	81896	G	C	intronic	MUC6	.
s9	chr11_KI270902v1_alt	81918	G	T	intronic	MUC6	.
s9	chr3_KI270935v1_alt	154208	A	G	exonic	MUC4	
